# Multiple oxidation states of uranium stabilized by an *O*,*N*,*O*-ligand

**DOI:** 10.1039/d5sc04158a

**Published:** 2025-11-17

**Authors:** Gabriel J. Juarez, Harris E. Mason, Daniel N. Mangel, Aaron M. Tondreau, Jonathan L. Sessler

**Affiliations:** a Department of Chemistry, The University of Texas at Austin 105 East 24th Street, Stop A5300 Austin Texas 78712-1224 USA sessler@cm.utexas.edu; b Los Alamos National Laboratory Los Alamos New Mexico 87544 USA tondreau_a@lanl.gov

## Abstract

Elements of the 5f row maintain a wide variety of oxidation states that have been exploited in synthesis, catalysis, and separations. Herein, we describe the complexation of an *O*,*N*,*O* chelator, ExPh, with uranium in the (IV) and (VI) (uranyl) oxidation states. These two uranium complexes, U(iv)ExPh and UO_2_ExPh, respectively, were characterized in the solid state *via* single-crystal X-ray diffraction (SC-XRD) analysis. Electrochemical studies using cyclic voltammetry were employed to investigate the multiple redox events associated with the U(iv) complex. Spectro-electrochemical analysis of U(iv)ExPh provided spectroscopic evidence of a stable U(v) species. Chemical oxidation of U(iv)ExPh allowed isolation of the U(v) complex, U(v)ExPh. All three stable uranium complexes produced in this study were characterized *via* IR, UV-vis and NMR spectroscopies, and micro-spectrophotometry. On the other hand, efforts to reduce U(iv)ExPh to the corresponding U(iii) species or produce this putative complex directly from ExPh failed to yield an isolable product. The stabilization of three formal oxidation states of uranium, coupled with previous lanthanide-row results, paves the way for studies of ExPh and its analogues in minor actinide chemistry.

## Abstract 2

Complexes derived from H_2_ExPh reveal differences in ligand framework between lanthanide and uranium ions. Structures are overlaid on the cyclic voltammogram of U(iv)ExPh.

## Graphical abstract

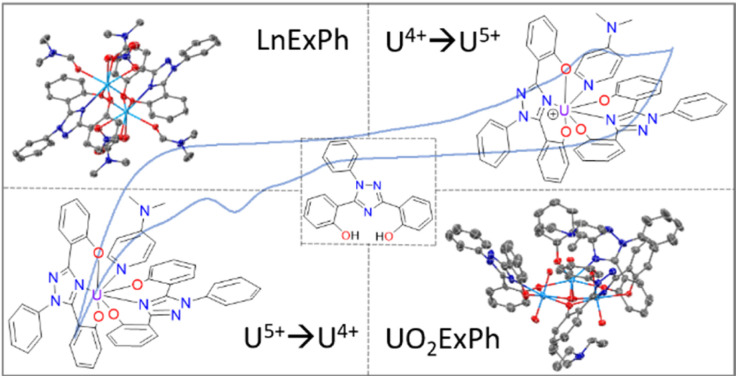

## Introduction

The use of actinide elements is widespread from medicine to industry, power production, and catalysis.^1–7^ Their utility arises not only from prominent radioactivity but also the accessibility of formal oxidation states ranging from (I)–(VII).^8–12^ Nevertheless, radiological and heavy element safety concerns, coupled with the unique bonding of the 5f orbitals, has resulted in less understanding of, and notably fewer, actinide complexes relative to their transition and lanthanide counterparts. Within the actinide row, uranium has been the most studied with molecules containing U(i) through U(vi) being reported.^8,13–19^ Limited, however, are ligands that have the capacity to complex with uranium in multiple oxidation states.^20,21^ Both tris(aryloxide) and tris(amide) complexes have been reported to stabilize U(iii) and U(iv) through a chemical oxidation with copper.^22^ A homoleptic trivalent uranium tetra-amidate complex was also shown to stabilize U(iv) upon chemical oxidation.^23^ Recently, a tris(amido)arene ligand was shown to form complexes with U(ii)–U(vi) in a retained framework.^24^ An expanded porphyrin, the product of a dedicated synthesis, was also found to stabilize both U(iv) and U(vi) complexes.^25^ These advances notwithstanding, there remains a need for simple-to-prepare ligands that display versatility in their uranium coordination chemistry.

Traditional iron-chelators have long been studied as complexants for f-block ions.^26,27^ The similarities in charge density, hard-acceptor, Lewis acidity, and interactions with iron-related biological proteins have provided an incentive for such studies.^28–31^ While polycarboxylic acids, such as diethylenetriaminepentaacetic acid, were originally studied,^26,30,32^ the discovery of biologically produced hydroxamic acid moieties found in bacteria and fungi for iron chelation and transport spearheaded the use of siderophore-type complexants for lanthanide and actinide ion coordination.^32–37^ However, of the ligands in question, few have received FDA approval for use as chelators. Medical treatments for contamination or ingestion of actinide ions constitute a need where chelation of radiotoxic nuclides by biocompatible ligands and controlled excretion could prove beneficial.^30,34,38^ ExJade is an FDA-approved iron-chelator that has yet to be studied for actinide ion coordination. While ExJade derivatives have shown remarkable therapeutic and sensing capabilities,^39,40^ advancing their medicinal utility to encompass radionuclides necessitates investigating their fundamental coordination chemistry with actinide ions. The present study was undertaken as a first step toward achieving this goal.

Previous work in our group established that ExJade analogues, a class of *O*,*N*,*O* chelating agents originally developed for the treatment of iron overload disease,^41–43^ have an ability to complex with lanthanide ions *via* a low-denticity coordination mode.^44^ Upon binding, cluster formation and solid precipitation resulted in the separation of Lu(iii) from the lighter lanthanides with good selectivity. While the later lanthanide complex formed a multi-metal-centered cluster, the early- and middle-row ions formed as 1 : 1 dimers. These binuclear structural motifs stand in stark contrast to other known f-element ion complexes. While studies of phenolic and nitrogen containing chelators, such as salen-derivatives, have provided insights into uranium complexes in the (IV) or (VI) oxidation states, few have probed the stabilization of a series of oxidation states using the same ligand or the more complicated multi-nuclear or multi-ligand speciation that could result from uranium ion complexation.^45–51^ We were thus keen to explore whether ExJade derivatives could (1) stabilize complexes with uranium in various oxidation states, (2) determine the ligand-to-metal stoichiometries, and (3) investigate their associated structural motifs. As detailed below, we have found that a readily accessible ExJade analogue, ExPh, supports complex formation with U(iv)–U(vi). Taken in concert, the resulting products not only define a variety of unique coordination motifs but also showcase the versatility of ExPh as a ligand capable of supporting uranium complexes in a range of formal oxidation states.

## Results and discussion

### Synthesis

**Scheme 1:**
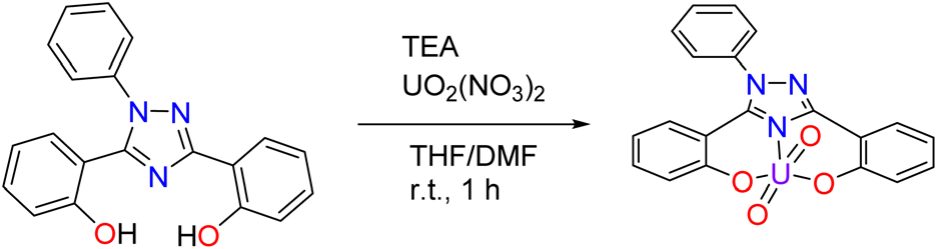
Synthesis of UO_2_ExPh performed under normal atmospheric conditions. The product formed as a trimer with a triethylammonium (HTEA) hydroxo-center. A simplified view of the ligand–uranyl complex is shown here.

Pro-ligand H_2_ExPh was synthesized as described previously.^40^ Initially, a uranyl ExPh complex (UO_2_ExPh) was synthesized using a protocol analogous to that used to prepare the ExJade lanthanide complexes reported earlier.^44^ Briefly, under normal atmospheric conditions, uranyl nitrate was added in stoichiometric fashion to a solution of ExPh in tetrahydrofuran (THF) containing 2.2 equivalents of triethylamine (TEA) as a base (Scheme 1). Precipitation occurred immediately upon mixing. A minimal amount of dimethylformamide (DMF) was added dropwise until full solubility was achieved. Crystallization of UO_2_ExPh occurred directly from the solvent mixture overnight. The isolated yield was 94%.

**Scheme 2:**
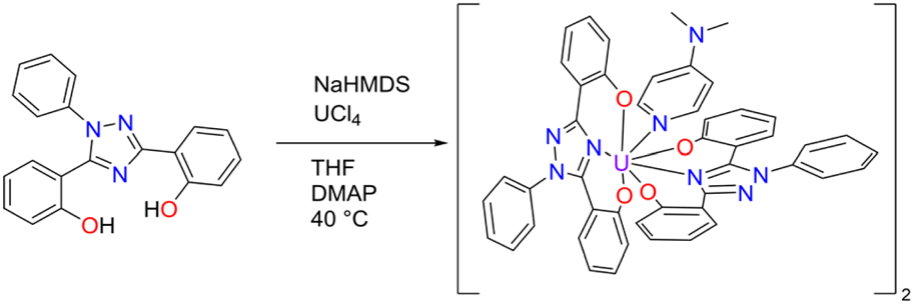
Synthesis of U(iv)ExPh under argon in a dry box. The product formed as a 2 : 1 dimer with 4-dimethylaminopyridine (DMAP) as a co-ligand.

To obtain the corresponding U(iv) complex, UCl_4_ was added to a solution of ExPh in THF in an argon-filled dry box in the presence of 2.2 equivalents of sodium bis(trimethylsilyl)amide (HMDS) used as a base (Scheme 2). When precipitation ceased, the reaction was deemed complete. Attempts at crystallization of the U(iv) product produced powders under varying conditions. Therefore, a secondary ligand was added to aid in crystallization. Specifically, 1 molar equivalent of 4-dimethylaminopyridine (DMAP) was added to the reaction product to serve as a co-ligand. With the addition of this co-ligand, the product slowly solubilized at elevated temperature. Crystallization was then effected *via* the vapor diffusion of *n*-hexane into the resulting solution at room temperature. This gave U(iv)ExPh in 89% yield as a dimeric solid.

**Scheme 3:**
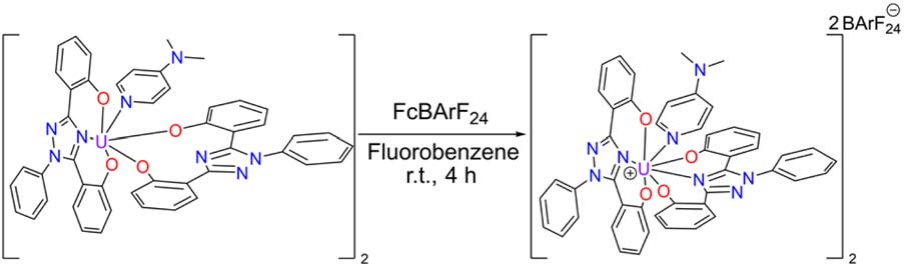
Synthesis of U(v)ExPh under argon in a dry box. The dimeric nature of the U(iv) starting material is retained in the product.

Cyclic voltammetry, *vide infra*, was performed on U(iv)ExPh to assess the redox chemistry of the complex. To obtain a U(v) complex of ExPh, U(iv)ExPh was oxidized by treatment with 1 molar equivalent (1 molar equivalents per metal center) of ferrocenium tetrakis(3,5-bis(trifluoromethyl)phenyl)borate (Fc^+^[BArF_24_]^−^) in fluorobenzene to give U(v)ExPh (Scheme 3). Initially, the optical characteristics of the solution were reminiscent of the ferrocenium salt. However, a darkening of the solution occurred over the course of the reaction yielding a deep brown product.

Next, attempts were made to prepare the lower oxidation state U(iii)-complex. The synthesis was attempted *via* both the stoichiometric incorporation of uranium tris(HMDS) to the pro-ligand H_2_ExPh and the chemical reduction of U(iv)ExPh with KC_8_. Both procedures resulted in a range of color changes as the reaction chemistry progressed. However, observation and analysis of the resulting equilibrium products, termed U(iii)_red_ExPh and U(iii)_HMDS_ExPh for sake of convenience (without implying a successful synthesis), were spectroscopically evocative of the corresponding U(iv) species. On this basis, we conclude that the species in question are either not formed or convert spontaneously to the higher oxidation state U(iv) form. A summary of the uranium chemistry carried out with ExPh is provided in Scheme S6.

### Structural characterization

**Fig. 1:**
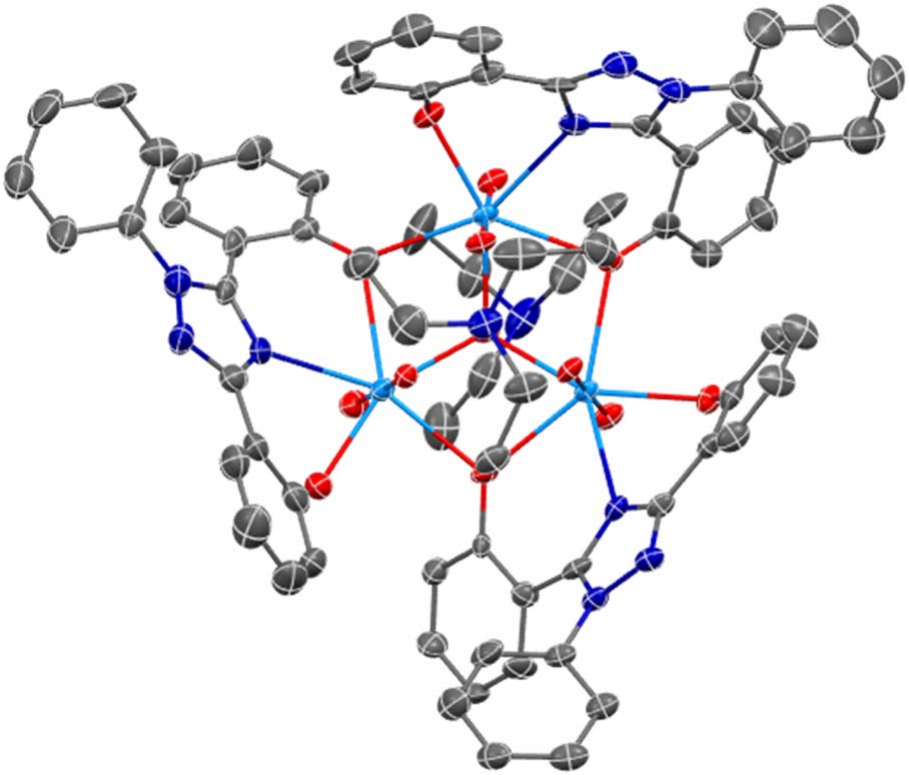
SC-XRD crystal structure of UO_2_ExPh revealing a trimeric, bowl-shaped complex (top-down view). A central hydroxo is contained within the complex that is capped axially by a triethylammonium cation. Ellipsoids are shown at the 50% probability level with hydrogens and solvent molecules omitted for clarity.

Complexes U(iv)ExPh and UO_2_ExPh yielded single crystals suitable for single crystal X-ray diffraction (SC-XRD) analysis. In the case of UO_2_ExPh the resulting structure revealed formation of a bowl-shaped trimeric complex with a single µ_2_-oxide from each ligand bridging two uranium metal centers. A central µ_3_-hydroxo bridge is contained within the trimeric bowl, capped axially by a triethylammonium (HTEA) counter cation (Fig. 1). This uranyl complex stands in contrast to the corresponding early- and middle-row lanthanide structures, which form as 1 : 1 dimeric species. The U–O bond lengths in UO_2_ExPh were found to be 2.315(5), 2.227(5), and 2.270(5) Å for the non-bridging phenolates and 2.458(5) and 2.434(5), 2.474(5) and 2.439(5), and 2.457(5) and 2.469(5) Å for the µ_2_-oxide bridging phenolates. The U–N_triazole_ distances were 2.559(5), 2.542(6), and 2.535(6) Å. The uranium-hydroxo bonds were 2.224(5), 2.263(5), and 2.265(5) Å. Twisting of the phenoxide arms resulted in torsion angles between 8.03 and 42.44°. The bending of the triazole core resulted in U–(N–C–N)_triazole_ torsion angles between 150.15 and 158.71°.

Solution-state ^1^H NMR spectroscopic studies of UO_2_ExPh in *d*_6_-DMSO revealed the loss of the phenol protons and only a subtle shifting of most peaks from what was seen for the pro-ligand H_2_ExPh (Fig. S1 and S2). The three ExPh ligands present in UO_2_ExPh were found to be in similar chemical environments as inferred from the chemical shift and integration values of key proton peaks. The triethylammonium proton peak was observed at 10.68 ppm, although the integration value was less than expected. This could reflect competition and exchange with the *d*_6_-DMSO solvent. Due to solubility limitations, corresponding ^1^H NMR spectral analyses were not performed in other solvents. Solution-state ^13^C NMR spectral analysis proved concordant with the ^1^H NMR studies in that a similar chemical environment was seen for each ExPh unit with non-solvent peaks only within the aromatic region between 110 and 170 ppm.

**Fig. 2:**
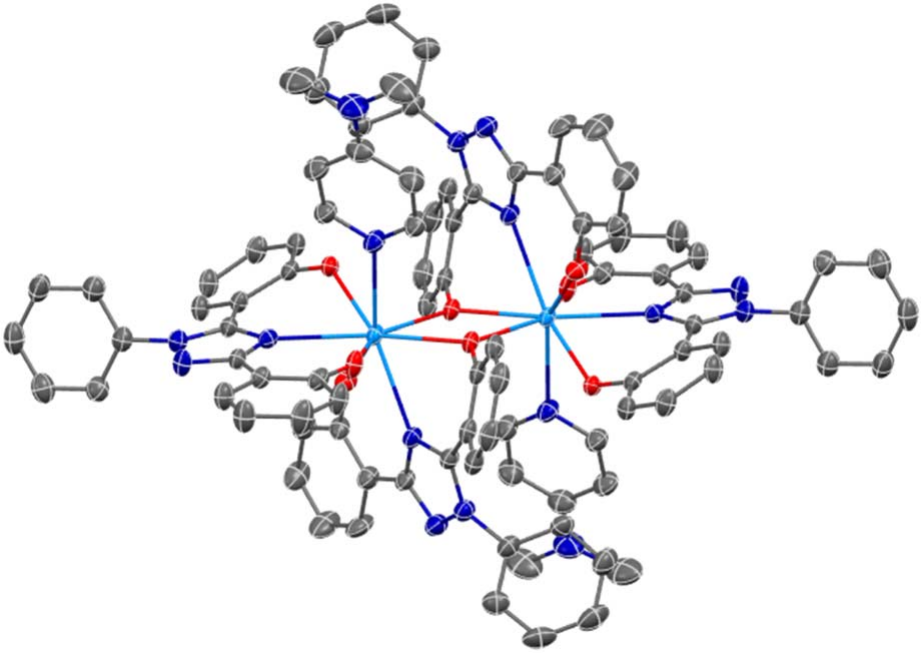
SC-XRD crystal structure of U(iv)ExPh revealing a dimeric complex containing a µ_2_-oxide bridge from one phenolate of each monomeric unit. Ellipsoids are shown at the 50% probability level with hydrogens and solvent molecules omitted for clarity.

An SC-XRD analysis of U(iv)ExPh revealed a notable difference in the coordinating ligand framework compared to UO_2_ExPh. The crystal formed as a 2 : 1 dimer with one ExPh ligand (L1) bridging two uranium centers and one terminal ExPh ligand (L2). One DMAP was coordinated to each uranium center yielding ([L1L2U(iv)][DMAP])_2_·THF (Fig. 2). Again, a stark contrast to the 1 : 1 dimeric lanthanide complexes was noted. The U–O bond lengths were 2.487(2) Å for the bridging phenolate and 2.164(3) Å for the terminal phenolate of ligand L1. The U–N_triazole_ bond length was 2.604(3) Å for L1 *versus* 2.576(3) Å for L2. The U–O bond lengths for L2 were 2.176(3) and 2.276(2) Å. The bridging phenolate of L1 revealed a torsion angle of 39.30° from the plane while the phenolate of L2 had a torsion angle of only 4.99°.

**Fig. 3:**
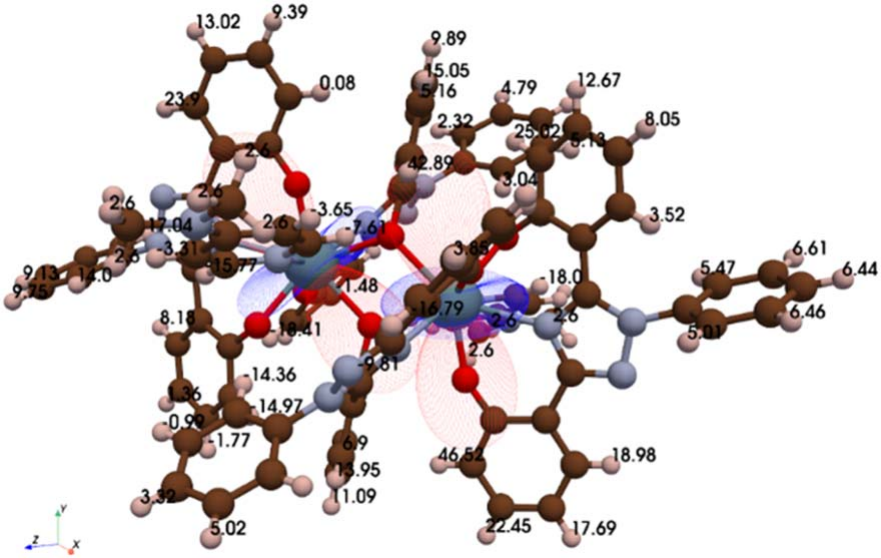
Results of a modelling study wherein the Δ*χ* U(iv) tensors are overlaid on the molecular structure. The numbers correspond to the individual calculated chemical shifts for each ^1^H spectral signal (referenced to TMS). The red and blue lobes represent the positive and negative portions of the Δ*χ* tensor, respectively.

Solution-state ^1^H NMR spectral studies revealed a large paramagnetic shift, between 44.1 and −17.9 ppm, ascribed to pseudo-contact with the coordinated U(iv) metal centers (Fig. S3). Unlike the uranyl complex, each proton in the 2 : 1 structure resulted in a separate peak. To identify the protons within the NMR spectrum, axial and radial *χ* magnetic susceptibility tensors were determined using a pseudo-contact shift (PCS) python algorithm (Fig. 3).^52^ The *χ* tensors, structural distances obtained from the crystal data, and pro-ligand ^1^H NMR chemical shifts were then used to predict the paramagnetic chemical shifts (*cf.* SI). For comparison, the magnetic susceptibility of U(iv)ExPh was measured on a magnetic susceptibility balance and found to match well with the predicted value (*χ*_iso,atomic_ = 4.34 *versus* 4.33 × 10^−32^ m^3^, respectively). The predictive model was used as a starting point for NMR spectral peak labelling (Table S1). ^13^C NMR spectral analyses were also performed; however no usable data could be obtained due to significant line broadening (Fig. S4).

Efforts to obtain single crystals of the proposed di-cationic U(v) dimer, U(v)ExPh, proved unsuccessful. As a result, solid-state structural data is limited for this complex. However, a ^1^H NMR spectral analysis (*d*_8_-THF) revealed significant changes in the chemical shifts of the signals relative to U(iv)ExPh (Fig. S5). The DMAP methyl peaks were still clearly identifiable along with the [BArF_24_]^−^ counter anion peaks. As opposed to the U(iv) complex with large paramagnetic shifts, the ^1^H NMR spectrum of U(v)ExPh was characterized by chemical shifts within the 17.4 and −0.91 ppm spectral window (Fig. S8). This range is as expected for a U(v) species.^24,53^ Nevertheless, in analogy to the U(iv) complex, each proton was considered to reside in a different magnetic environment. The net result was a complicated spectrum whose chemical shift values could not be assigned completely.

The putative U(iii) complexes prepared through reduction of the U(iv) form and direct use of an HMDS starting material (*vide supra*) were likewise characterized by ^1^H NMR spectroscopy. The reaction product prepared through reduction gave rise to a spectrum characterized by proton shifts that were nearly identical to those seen for U(iv)ExPh (Fig. S6). A few of the peaks were shifted; however, this could reflect the presence of free potassium ions arising as a byproduct of the reduction process. The species referred to as U(iii)_HMDS_ExPh also gave a nearly identical spectrum, except for the clearly identifiable bis(trimethylsilyl) proton peaks (Fig. S7). An overlay of the NMR spectra for U(iv)ExPh and the products of both attempted preparations of a U(iii) form (Fig. S9) highlighted the similarities between the species in question. This correspondence, along with the previous optical spectroscopic findings, leads us to suggest that U(iii) ExPh complexes are unstable, and, if formed, are transformed readily into the corresponding U(iv) species upon attempted isolation. This presumed instability could be the result of disproportionation of the U(iii) centers resulting in generation of the dimeric U(iv) product and a U(ii) anionic pair^54–56^ or alternatively through alcoholysis of the phenol to yield the U(iv) dimeric complex.^57^

### Electrochemical measurements

**Fig. 4:**
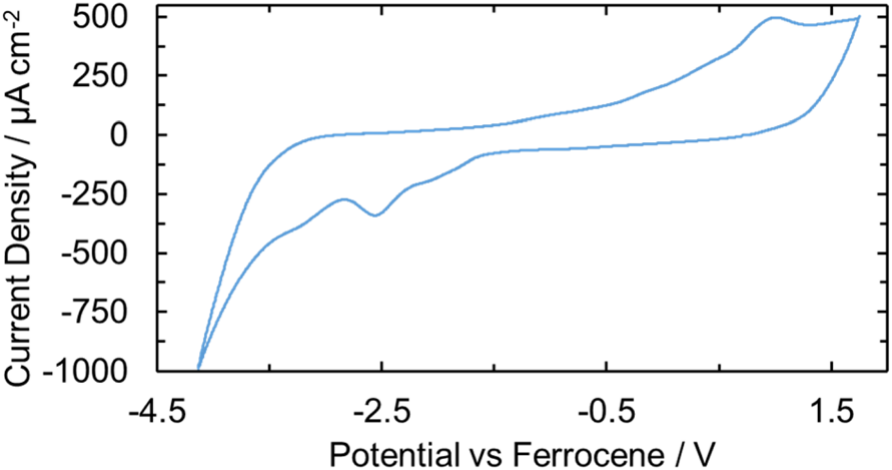
CV of U(iv)ExPh as a 1 mM solution in THF containing 100 mM TBAPF_6_ as the supporting electrolyte at a scan rate of 100 mV s^−1^ performed on a glassy carbon electrode. A large, irreversible redox couple is seen at *E*_pa_ = 1.01 V and *E*_pc_ = −2.55 V. The large peaks are preceded by two smaller and broader peaks.

An electrochemical study was performed on U(iv)ExPh to determine the stability of the lower and higher valent uranium species produced upon formal gain or loss of an electron. The experiments were carried out using THF solutions containing 1 mM of the U(iv) complex and 100 mM supporting electrolyte (TBAPF_6_). The open-circuit potential was initially recorded to determine the equilibrium voltage of the system. Cyclic voltammetry was then performed to assess the accessible oxidation states of the complex. Two irreversible waves were observed for the oxidation, one peak being a smaller pre-wave and the second being a larger irreversible couple (Fig. 4). The pre-wave is thought to reflect a small amount of a monomeric U(iv) complex in solution, which is slightly easier to access electrochemically than the crystallographically characterized 2 : 1 complex. Upon addition of ferrocene, the pre-wave was no longer seen in the oxidative CV scan, while the large anodic peak was characterized by an accelerated onset and substantial broadening (Fig. S29). The ferrocenium reduction wave was also not seen. On the basis of a differential pulse voltammetric analysis (Fig. S30), we conclude that the ferrocene acts as an electron transfer mediator to the U(iv) complex. Thus, following ferrocene oxidation a non-zero steady state current is achieved and only a single oxidation wave is seen. Additional voltammetry experiments were performed in the presence of decamethylferrocene to provide a well-behaved internal reference (Fig. S31). In this case, both the forward and reverse peaks of the iron-reference were observed, along with the pre-waves and oxidation and reduction peaks of the U(iv) complex. Further voltametric analyses were conducted to elucidate the correlation between the oxidation and reduction peaks seen for the U(iv) complex (*cf.* SI, pp S34 and S35).

As a prelude to the Fc^+^[BArF_24_]^−^-based oxidation studies described above, spectro-electrochemical studies were performed on U(iv)ExPh with the goal of characterizing further the product produced upon oxidation. A potential step from −1.0 to 1.5 V was pulsed for 0.5 s. A UV-vis spectrum was taken before and after the potential step. The post-step spectrum revealed the appearance of a broad peak in the visible region between 450 and 650 nm (Fig. S32a). Optical observation of the solution in the cuvette revealed the presence of a deep purple/brown solution shortly after the potential step. Chemical oxidation was then attempted by injecting atmospheric air as a sequence, 0.1 mL aliquots into a cuvette containing 1.5 mL of a 1 mM solution of U(iv)ExPh in THF. The UV-vis spectra were recorded shortly after each injection and after a final 0.5 mL injection of air. The resulting spectrum proved similar to that seen during the spectro-electrochemical oxidation study (Fig. S32b). The physical appearance of the samples also matched. The apparent ease with which U(iv)ExPh underwent both electrochemical and air-based oxidation, led us to consider that a U(v) species could be prepared under preparative conditions using ferrocenium as the chemical oxidant as described above.

In contrast to what was seen under conditions of oxidation, a corresponding spectro-electrochemical study revealed no discernible U(iv)/U(iii) redox events upon reduction. This was taken as further support for the instability of the U(iii) ExPh complex.

### Solution- and solid-state spectroscopy

**Fig. 5:**
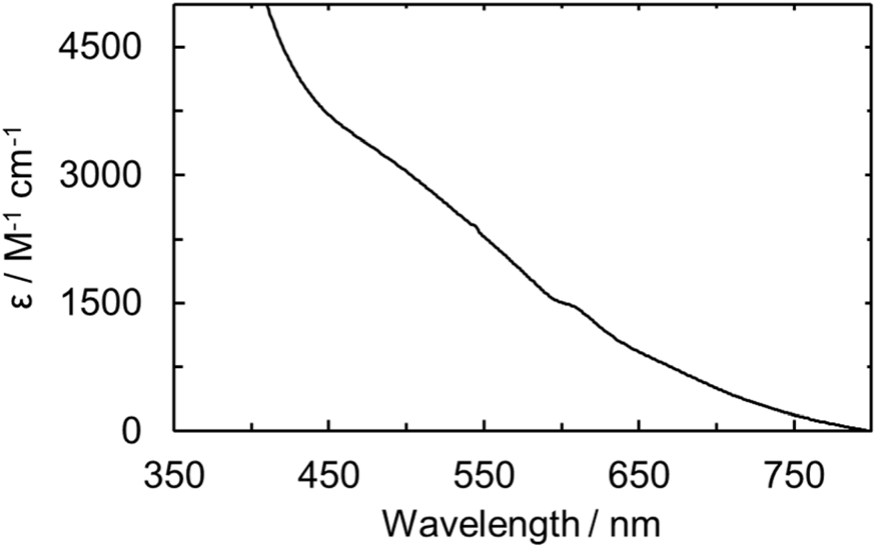
UV-vis of U(v)ExPh measured in fluorobenzene at room temperature. A broad shoulder is observed with maximum at wavelength of 474 nm and a sharper peak at 610 nm.

The UO_2_ExPh, U(iv)ExPh, U(v)ExPh, and the aspirational U(iii)_HMDS_ExPh complexes were further analyzed by UV-vis and IR spectroscopies. In the case of the uranyl complex, the UV-vis spectrum revealed a shoulder at 456 nm with a molar absorptivity of 1420 (±30) M^−1^ cm^−1^ (Fig. S12 and S13). The spectrum for the corresponding U(iv) complex was characterized by the presence of several peaks at 674, 642, 625, and 545 nm ascribed to f–f transitions. An additional broad peak at 562 nm was also seen that was determined to be non-linear in concentration (Fig. S13). The molar absorptivity values for the linear-in-concentration peaks were determined to be 54 (±3), 44 (±1), 33.4 (±0.3), and 8 (±2) M^−1^ cm^−1^, respectively (Fig. S14). A UV-vis spectral analysis of the U(v) complex revealed a broad absorption feature between 450 and 650 nm analogous to that observed in the spectro-electrochemical study. An absorption maximum at 474 nm with a molar absorptivity of 2920 (±450) M^−1^ cm^−1^ was seen (Fig. 5). The deep coloration of U(v)ExPh is ascribed to this broad absorption feature, which has been observed previously in various reported U(v) complexes.^24,58–61^

The putative U(iii) complexes displayed a small peak at 607 nm in the UV-vis spectrum that proved non-linear with concentration, as well as a broad peak at 672 nm with a molar absorptivity of 100 (±30) M^−1^ cm^−1^ (Fig. S17). The similarity of this spectrum and that recorded for U(iv)ExPh was taken as further support for the conclusion that the ExPh ligand does not stabilize a U(iii) center effectively, and that if produced, the low-valent species converts readily to the corresponding U(iv) complex. Solution-state NIR spectra revealed no discernible features between the uranyl, U(iv), and putative U(iii) complexes. The lack of distinction from baseline is ascribed to the poor solubility of the complexes and inability to obtain concentrations necessary for analysis. Solution-state near infrared spectroscopy of the U(v) complex revealed a characteristic 5f^1^ absorption peak. Specifically, a single sharp peak was noted at 1444 nm, consistent with expectations for a U(v) complex (Fig. S16).^24,58,62–64^

**Fig. 6:**
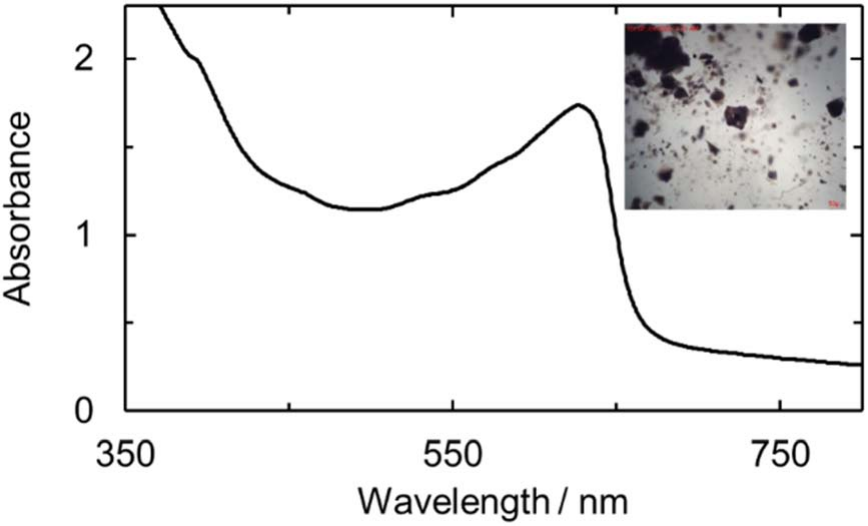
Micro-spectro photogram of U(v)ExPh obtained from crystallized material. A broad shoulder is seen at 394 nm with a sharp peak at 629 nm complementing the solution-state UV-vis spectrum. The inset shows an image of the deep brown/purple plate crystals used for the spectroscopic analysis. Twinning and desolvation is revealed by the several fractures among the crystals.

The purified uranyl, U(iv), and U(v) complexes were studied spectroscopically in the solid state using micro-spectrophotometry (Fig. 6, and S21–S23). Each spectrum revealed absorption features similar in shape and wavelength maximum to the corresponding solution-state spectra, albeit with slight red-shifts.

Solid-state IR data were also obtained for the uranyl, U(iv), U(v) and putative U(iii) complex, (Fig. S18–S20) revealing aromatic C–H and C=C stretches. The triethylammonium and hydroxy groups of UO_2_ExPh showed N–H and O–H stretches in the form of a broad peak around 3400 cm^−1^. UO_2_ExPh, U(iv)ExPh, and the putative U(iii)_HMDS_ExPh complex showed alkyl C–H stretching modes around 2950 cm^−1^ ascribed to their counterions and co-ligands – triethylammonium, DMAP, and HMDS, respectively. A unique [BArF_24_]^−^ C–F stretching mode was also observed for the U(v)ExPh complex at 1110 cm and 670 cm^−1^; these features were not seen for the other complexes.

## Conclusions

In this study we show that the simple-to-prepare ligand, ExPh, recently found effective in lanthanide cation complexation, can stabilize crystallographically characterized complexes with uranium as U(iv) and U(vi) (uranyl). Electrochemical studies of the U(iv) complex, U(iv)ExPh, provided support for the conclusion that oxidation to a stable U(v) complex could be achieved. Consistent with this supposition, treatment of U(iv)ExPh with ferrocenium tetrakis(3,5-bis(trifluoromethyl)phenyl)borate afforded U(v)ExPh. In addition to SC-XRD structural analyses of single crystals of the U(iv) and U(vi) complexes, the three stable products of this study, namely U(iv)ExPh, U(v)ExPh, and UO_2_ExPh, were characterized in the solid state by IR spectroscopy and micro-spectrophotometry, as well as in solution by ^1^H NMR spectroscopy. Efforts were made to prepare the corresponding U(iii) species. However, a combination of electrochemical and chemical studies revealed no sign that a stable species was formed. We thus conclude that ExPh as a ligand system is not able to stabilize a U(iii) metal complex. Nevertheless, the ability to coordinate with uranium cations in three sperate formal oxidation states, namely U(iv), U(v), and U(vi), highlights the efficacy of this *O*,*N*,*O* chelator toward actinide complexation. This is expected to pave the way for future studies involving transuranic ion chelation, as well as in due course biological evaluations of *inter alia* safety and pharmacokinetic parameters.

## Author contributions

This work was conceived and executed at both the Los Alamos National Laboratory (LANL) and The University of Texas at Austin (UTA) by all authors. G. J. J. and D. N. M. conducted the synthetic work. Formal data analysis of PCS studies were performed by H. E. M. Structural data collection and refinement was carried out by A. M. T. Manuscript preparation and revisions were made by G. J. J., A. M. T., and J. L. S.

## Conflicts of interest

There are no conflicts to declare.

## Supplementary Material

SC-017-D5SC04158A-s001

SC-017-D5SC04158A-s002

## Data Availability

Supplementary information: experimental, photographs of compounds, characterization spectra, details regarding the pseudo-contact shift modelling, and crystallographic data (CIF) for all complexes. The code used for pseudo-contact shift determinations will be provided by A. M. T. upon request. See DOI: https://doi.org/10.1039/d5sc04158a. CCDC 2445901 and 2445902 contain the supplementary crystallographic data for this paper.^65*a*,*b*^
