# Supplementary material for: Multiple oxidation states of uranium stabilized by an O,N,O-ligand
Source: Chem Sci. 2025 Nov 17;17(2):1294–300. doi: 10.1039/d5sc04158a (PMC12643114; doi:10.1039/d5sc04158a)
Supplement: SC-017-D5SC04158A-s001 [file SC-017-D5SC04158A-s001.pdf]

Supplementary Information (SI) for Chemical Science.
This journal is © The Royal Society of Chemistry 2025

Multiple oxidation states of uranium stabilized by an *O, N, O*-ligand

Gabriel J. Juarez,[a] Harris E. Mason,[b] Daniel N. Mangel,[a] Aaron M. Tondreau,[b*] and Jonathan L. Sessler[a*]

[a]*Department of Chemistry, The University of Texas at Austin, 105 East 24th Street, Stop A5300, Austin, Texas 78712-1224, USA.*

[b]*Los Alamos National Laboratory, Los Alamos, New Mexico 87544, United States*

Table of Contents

General Considerations S3

Synthetic Protocols S4 – S7

NMR Spectra S8 – S16

NMR Pseudo-Contact Shifts S17 – S19

UV-Vis-nIR Spectra S20 – S23

IR Spectra S24 – S25

Solid-State Images and Spectra S26 – S28

Electrochemical Studies S29 – S36

Crystal CCDC Deposition Numbers and Data Tables S37 – S54

References S55

General Considerations

Materials. The solvent THF was degassed, dried over Na/K overnight, and filtered through dry, neutral alumina, before storage over 4 Å molecular sieves. Fluorobenzene was degassed, filtered through dry, neutral alumina and stored over 4 Å molecular sieves. n-Hexane was degassed, dried over Na/K overnight, filtered through dry, neutral alumina and stored over 4 Å molecular sieves. d_8-THF was purchased from Cambridge Isotope Laboratories. d_6-DMSO was purchased from Alfa Aesar and used as received. All other NMR solvents were degassed and dried over Na/K overnight and filtered through dry, neutral alumina, before storage over 4 Å molecular sieves. **ExPh** was synthesized as previously reported.[1] Uranium(IV) chloride was synthesized according to previous procedures.[2] 4-Dimethylaminopyridine (DMAP) and ferrocenium tetrafluoroborate were purchased from Sigma Aldrich. DMAP was recrystallized before use. Ferrocenium tetrafluoroborate was converted to the tetrakis[3,5-bis(trifluoromethyl)phenyl]borate salt and crystallized before use according to literature procedures.[3] KC_8 was prepared from potassium metal and graphite flakes.

General Considerations. All air- and moisture-sensitive manipulations were carried out using standard Schlenk techniques or in a VAC-ATM Omnilab dry box containing a purified argon atmosphere. ^{1}H and ^{13}C NMR spectra were recorded on a Bruker Avance 400 MHz spectrometer operating at 400.132 and 100.580 MHz, respectively. Samples of air-sensitive compounds for NMR spectral analysis were prepared using a Teflon liner for double-containment and sealed with puddy to prevent atmosphere from seeping inside. UV-Vis-NIR spectra were collected at room temperature on a Cary5000 UV-Vis-NIR spectrophotometer (Agilent Technologies). Infrared spectra were obtained at room temperature on a Bruker Alpha II using the OPUS software version 8.7.31. Electrochemical measurements were carried out at room temperature using an integrating Pine WaveNow Wireless potentiostat from Pine Research Instrumentation Inc. and the AfterMath version 1.6.11275 software. Solid-state absorbance spectra and images were captured on a Craic QDI 2010 UV-Vis/NIR using the Lambdafire software version 1.2.84.8. Magnetic susceptibility measurements were performed on a Sherwood Scientific MSB Auto magnetic susceptibility balance.

Crystallography. Single crystals suitable for X-ray diffraction were coated with Krytox™ in a dry box, placed on a nylon loop and then transferred to the goniometer head of a Bruker AXS D8 Quest diffractometer equipped with a graphite-monochromatized molybdenum Kα X-ray tube (λ = 0.71073 Å) and a CMOS detector, or to a Bruker D8 Quest using a molybdenum Kα X-ray tube (λ = 0.71073 Å) IμS 3.0 Microfocus source X-ray generator.[4] A hemisphere routine was used for data collection and determination of lattice constants. The space group was identified, and the data was processed using the Bruker SAINT+ program and corrected for absorption using SADABS.[5-7] The structures were solved using direct methods (SHELXS), completed by subsequent Fourier synthesis, and refined by full-matrix least-squares procedures.[8,9] The Olex2 software was used as the graphical interface.[10,11] Crystallographic data for all structures is available from the Cambridge Structural Database by quoting the following CCDC numbers: 2445901 and 2445902.

Synthetic Protocols

Synthesis of UO_2ExPh:

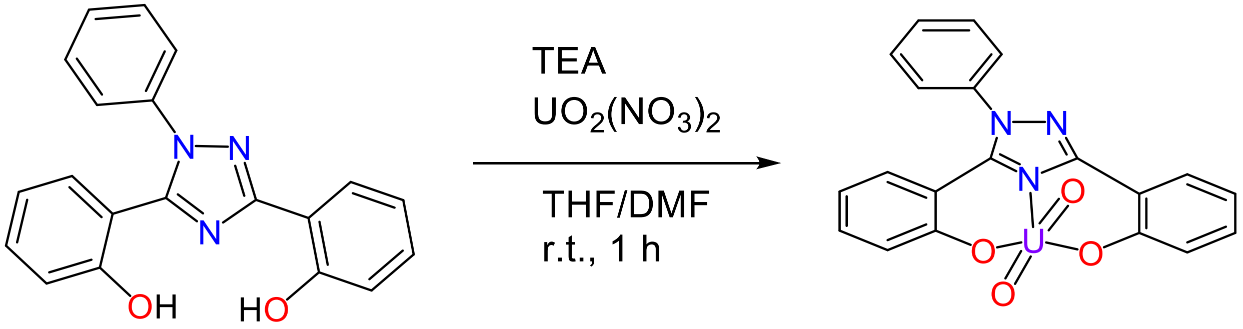

Scheme S1. Schematic showing the complexation reaction used to prepare **UO_2ExPh**.

To a 2-dram vial was added **H_2ExPh** (8.5 mg, 0.026 mmol) dissolved in 0.5 mL of THF. Triethylamine was added (8 µL) and the vial was stirred briefly. To a separate 2-dram vial was added uranyl nitrate (13.0 mg, 0.026 mmol). The ExPh solution was transferred to the uranyl solution, and the reaction immediately produced a precipitate. The solids were dissolved via the dropwise addition of DMF, and the vial was allowed to rest overnight to form orange plate crystals. The solution was decanted, and the crystals dried under reduced pressure (14.8 mg, 94.4%). Analysis of **UO_2ExPh** ($C_{72}H_{71}N_{11}O_{13}U_3$): ^{1}H NMR ($d_6$-DMSO, 400 MHz, 25 °C): δ 0.88 (t, 9H, J = 7.3 Hz, TEA), 2.70 (q, 6H, J = 7.2 Hz, TEA), 6.09 (t, 1H, J = 7.5 Hz), 6.42 (t, 1H, J = 7.4 Hz), 6.65 (t, 1H, J = 7.8 Hz), 6.89 (d, 1H, J = 8.2 Hz), 6.95 (d, 1H, J = 8.3 Hz), 7.13 (t, 1H, J = 7.5 Hz), 7.22 (quint, 2H, J = 7.8 Hz), 7.46 (qui, 4H, J = 7.6 Hz), 7.84 (d, 1H, J = 7.6 Hz), 10.68 (s, TEAH+). ^{13}C{^{1}H} NMR (d_6-DMSO, 100 MHz, 25 °C): δ 9.4 (s, TEA), 30.9 (s, DMF), 35.9 (s, DMF), 45.9 (s, TEA), 113.7 (s), 115.3 (s), 116.5 (s), 117.7 (s), 120.8 (s), 121.2 (s), 164.4 (s), 127.2 (s), 128.6 (s), 130.0 (s), 131.3 (s), 132.4 (s), 138.8 (s), 152.5 (s), 158.1 (s), 162.5 (s), 168.0 (s), 169.8 (s). This complex was further characterized by a single crystal X-ray diffraction analysis.

Synthesis of U(IV)ExPh:

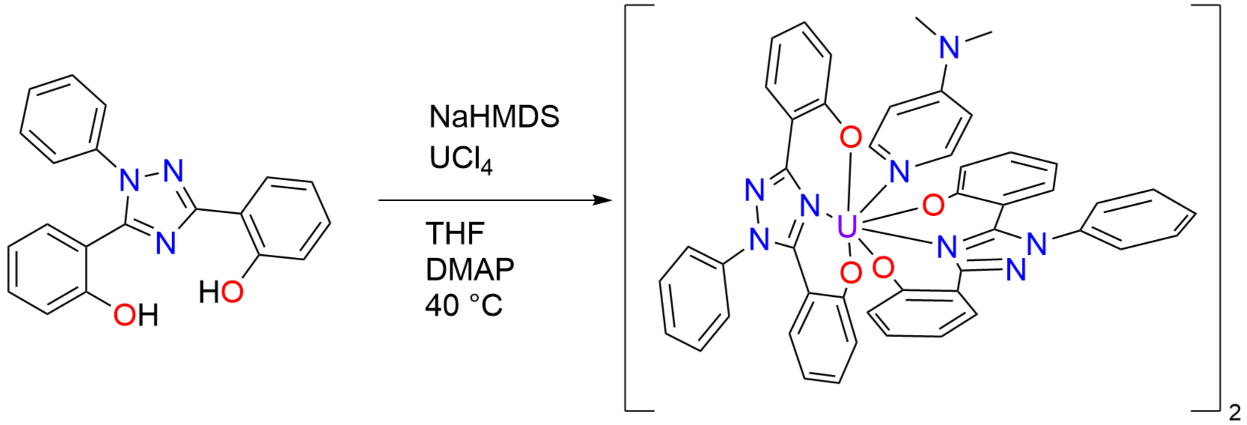

Scheme S2. Schematic showing the complexation reaction used to prepare **U(IV)ExPh**.

In an argon-filled dry box, a dry 20 mL vial was charged with **H_2ExPh** (200 mg, 0.601 mmol) and 5 mL dry THF. Sodium bis(trimethylsilyl)amide was added (222.7 mg, 1.214 mmol), and the solution was stirred for several minutes. Anhydrous uranium tetrachloride was added to the solution (115.3 mg, 0.304 mmol). The reaction was heated to 40 °C for 1 hour with minor precipitation. DMAP was added to the reaction (74.2 mg, 0.607 mmol), and the precipitate slowly dissolved over an additional 4 hours. After this time, the mixture was filtered, and the solvent was removed under reduced pressure. The resulting

pale green powder was dissolved in fluorobenzene and crystallized by vapor diffusion of n-hexane yielding pale green plate crystals (273.4 mg, 88.5%). Analysis of **U(IV)ExPh** ($C_{94}H_{72}N_{16}O_8U_2$): Expected: C = 55.62%, H = 3.58%, N = 11.04%. Measured: C = 55.71%, H = 3.82%, N = 11.16%. ^{1}H NMR (d_8-THF, 400 MHz, 25 °C): δ -17.92 (br), -17.16 (s), -16.59 (s), -9.98 (s), -4.37 (s), -3.78 (s), -1.35 (br), -0.96 (s), -0.45 (s), 0.03 (t, J = 9.0 Hz), 1.13 (d, 4.6 Hz), 2.50 (br, DMAP), 4.35 (br), 4.69 (s), 5.37 (br), 5.67 (br), 6.28 (s), 6.46 (s), 6.61 (s), 7.03 (br), 7.88 (br), 8.21 (s), 10.26 (s), 10.71 (s), 11.97 (br), 13.79 (s), 14.70 (br), 16.59 (br), 19.94 (s), 23.32 (s), 44.07 (br). ^{13}C{^{1}H} NMR (d_8-THF, 100 MHz, 25 °C): δ 119.5 (s), 122.2 (s), 127.8 (s), 129.1 (s). The effective magnetic moment was determined to be 3.15 μ_β; n = 2.31. This complex was further characterized by a single crystal X-ray diffraction analysis.

Synthesis of U(V)ExPh:

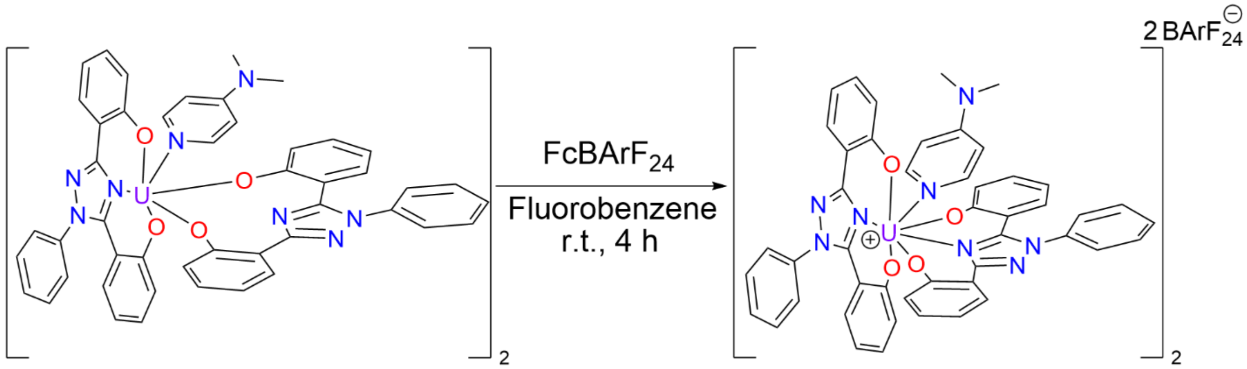

Scheme S3. Schematic showing the oxidation reaction used to prepare **U(V)ExPh**.

In an argon-filled dry box, a 20 mL vial was charged with **U(IV)ExPh** (50 mg, 0.025 mmol) and 1 metal equivalent of ferrocenium $BArF_{24}$ (51.6 mg, 0.049 mmol) in dry fluorobenzene. The reaction was stirred at room temperature for 4 hours yielding a deep purple solution. The solvent was removed under reduced pressure, and the powder was washed with dry n-hexane 3 times to remove ferrocene (orange solution). The final $[BArF_{24}]^-$ product was further purified by crystallization from a layered fluorobenzene/n-hexane two-solvent mixture to yield deep-purple, plate crystals (60.8 mg, 65.7%). Analysis of **U(V)ExPh** ($C_{158}H_{96}N_{16}O_8U_2B_2F_{48}$): Expected: C = 50.52%, H = 2.58%, N = 5.97%. Measured: C = 50.36%, H = 3.05%, N = 5.82%. ^{1}H NMR (d_8-THF, 400 MHz, 25 °C): δ -0.91 (s), -0.19 (s), -0.03 (s), 0.01 (s), 0.07 (s), 0.25 (s), 0.38 (s), 0.73 (t, J = 7.3 Hz), 1.55 (s), 1.88 (s), 2.04 (s), 2.35 (br, DMAP), 3.27 (s), 3.75 (s), 4.12 (s), 4.87 (d, J = 5.9 Hz), 5.85 (s), 6.18 (s), 6.34 (br), 6.60 (br), 6.88 (br), 7.06 (d, J = 8.8 Hz), 7.30 (s), 7.69 (d, J = 7.4 Hz), 7.99 (s), 8.21 (d, 8.3 Hz), 8.83 (s), 12.02 (s), 12.54 (s), 13.48 (br), 16.15 (br), 17.37 (br).

Attempted synthesis of $[U(III)ExPh_2(DMAP)]_2^-[K^+]_2$:

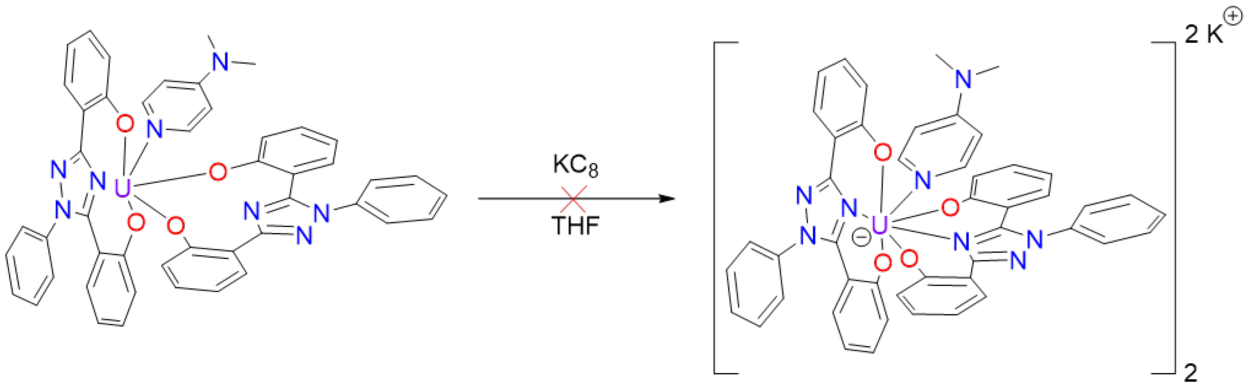

Scheme S4. Attempted synthesis of **$U(III)_{red}$ExPh** via reduction.

In an argon-filled dry box, a 20 mL vial was charged with **U(IV)ExPh** (25 mg, 0.012 mmol) and KC_8 (3.3 mg, 0.025 mmol). The reaction mixture was stirred in 4 mL of dry tetrahydrofuran at room temperature for 4 h. This resulted in a wide variety of color changes – pale green to blue to bright orange, before equilibrating as a pale-yellow solution. The reaction was filtered to remove graphite and concentrated under reduced pressure followed by washing with n-hexane to yield a light-yellow product. Analysis of the material referred to as **$U(III)_{red}$ExPh** ($C_{94}H_{72}N_{16}O_8U_2K_2$) (a designation not meaning to imply a successful synthesis; cf. main text): ^{1}H NMR (d_8-THF, 400 MHz, 25 °C): δ -51.53 (s), -6.09 (s), -3.80 (s), -2.90 (s), -1.60 (s), -0.19 (s), 0.10 (s), 0.25 (s), 1.15 (s), 2.04 (s), 2.92 (br, DMAP), 4.67 (t, J = 6.4 Hz), 5.36 (t, J = 7.8 Hz), 5.63 (t, J = 7.2 Hz), 6.11 (s), 6.43 (br), 6.69 (br), 7.48 (s), 8.00 (br), 9.64 (s), 10.75 (s), 11.39 (s), 12.95 (s), 13.20 (s), 13.88 (s), 14.39 (br), 14.86 (s), 17.59 (br), 18.70 (s), 19.86 (s), 23.34 (s), 35.80 (br).

Attempted synthesis of $[U(III)ExPh(HMDS)]_2$:

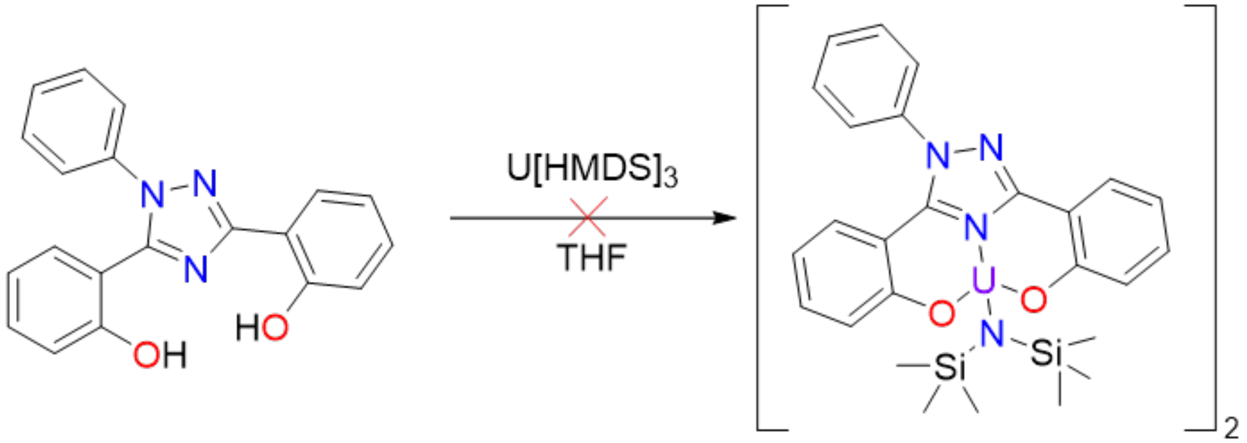

Scheme S5. Schematic view of complexation reaction used for the attempted synthesis of **$U(III)_{HMDS}$ExPh**.

In an argon-filled dry box, a 20 mL vial was charged with **H_2ExPh** (50 mg, 0.152 mmol) and U[tris(bis(trimethylsilyl)amide)] (110 mg, 0.152 mmol). The reaction mixture was stirred in 4 mL of dry

tetrahydrofuran at room temperature for 4 h followed by concentration under reduced pressure. The clear/yellow, crude putative product was washed with n-hexane (3 x 2 mL) and further purified by crystallization from layered fluorobenzene/n-hexane to yield a yellow powder. Analytical data for the material referred to as **U(III)$_{HMDS}$ExPh** ($C_{52}H_{62}N_8O_4U_2Si_4$) (a designation not meaning to imply a successful synthesis; cf. main text): ^{1}H NMR (d_8-THF, 400 MHz, 25 °C): δ -23.43 (s), -11.98 (br), -10.05 (br), -9.76 (br), -7.19 (br), -6.99 (br), -5.33 (s), -4.14 (br), -3.04 (s), -2.56 (s), -2.09 (s) -1.79 (s), -1.26 (s), 1.16 (s), 4.88 (s), 5.57 (s), 6.38 (s), 6.71 (s), 7.30 (br), 7.79 (s), 10.09 (s), 10.87 (s), 12.46 (s), 13.88 (s), 19.85 (s), 23.32 (s).

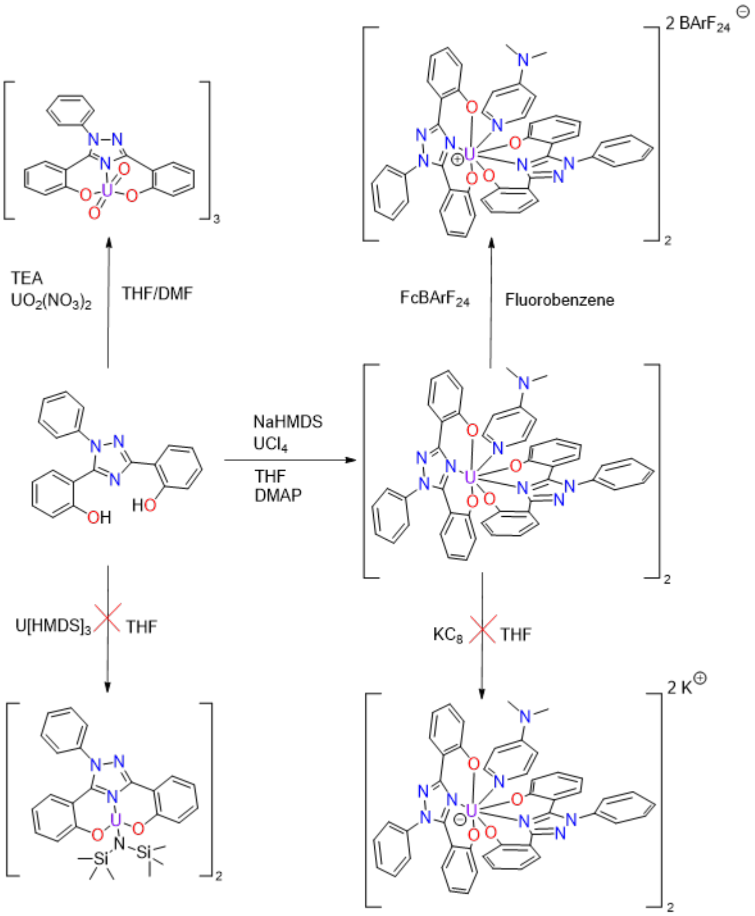

Scheme S6. Synthetic procedures used to prepare various uranium complexes stabilized by **ExPh**. The nature of the product formed and associated coordination chemistry was found to vary as a function of uranium oxidation state with either trimers or dimers being obtained. See main text for details.

NMR Spectra

[$(UO_2ExPh)_3OH]^-[(HTEA)]^+$:

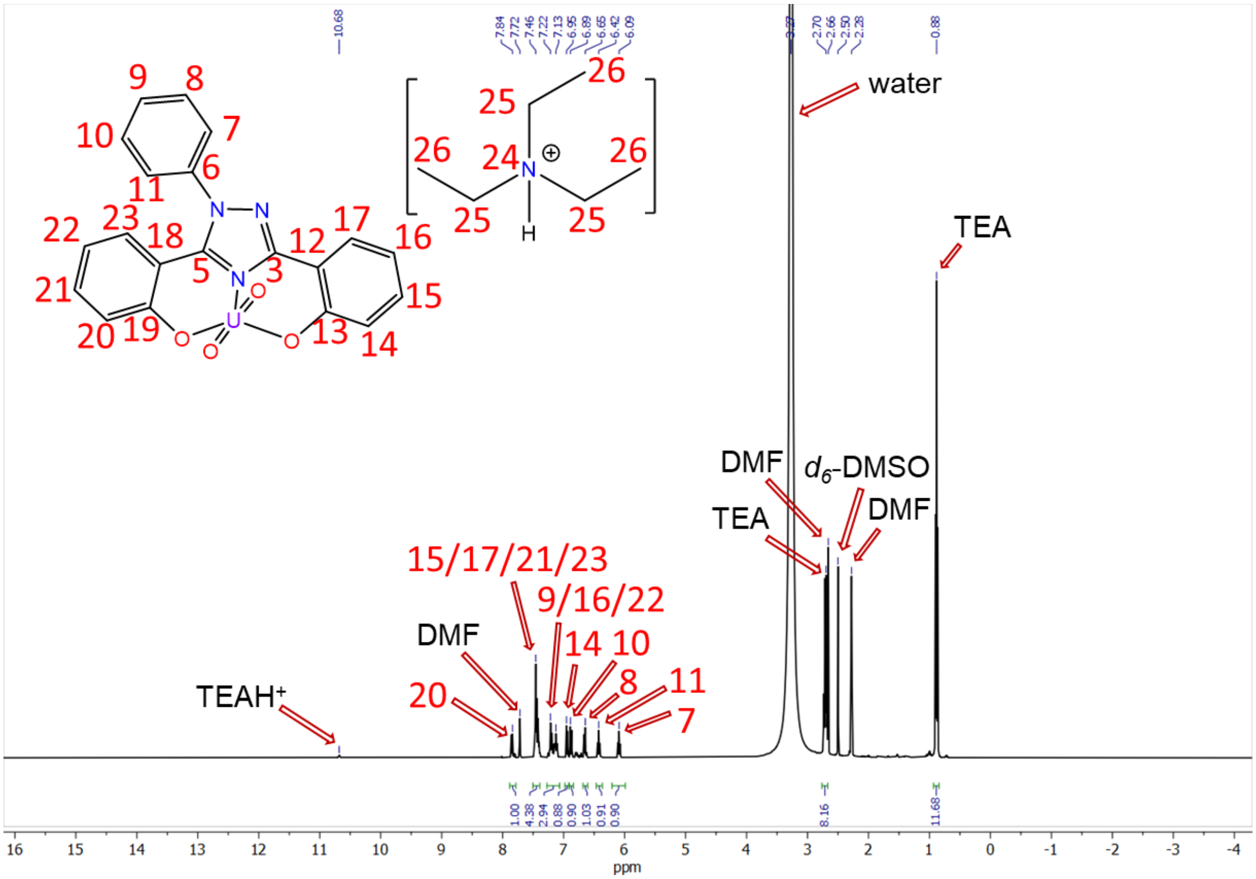

Figure S1. ^{1}H NMR spectrum obtained on a Bruker Avance Neo 400 Nano of uranyl ExPh compound (**UO_2ExPh**) run in d_6-DMSO. All product and residual solvent peaks are labeled.

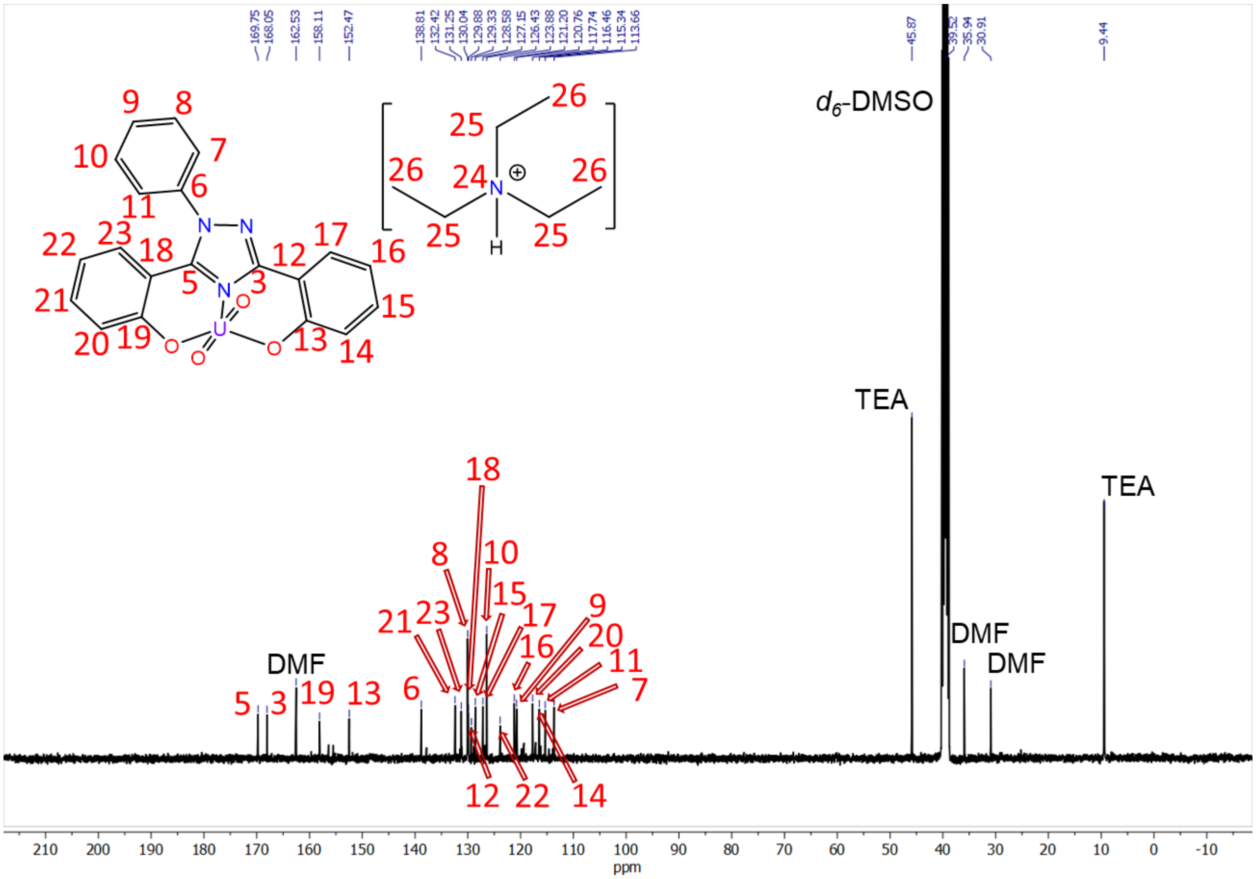

Figure S2. $^{13}C\{^{1}H\}$ NMR spectrum obtained on a Bruker Avance Neo 400 Nano of **UO_2ExPh** recorded in d_6-DMSO. All product and residual solvent peaks are labeled.

[U(IV)ExPh$_2$(DMAP)]$_2$:

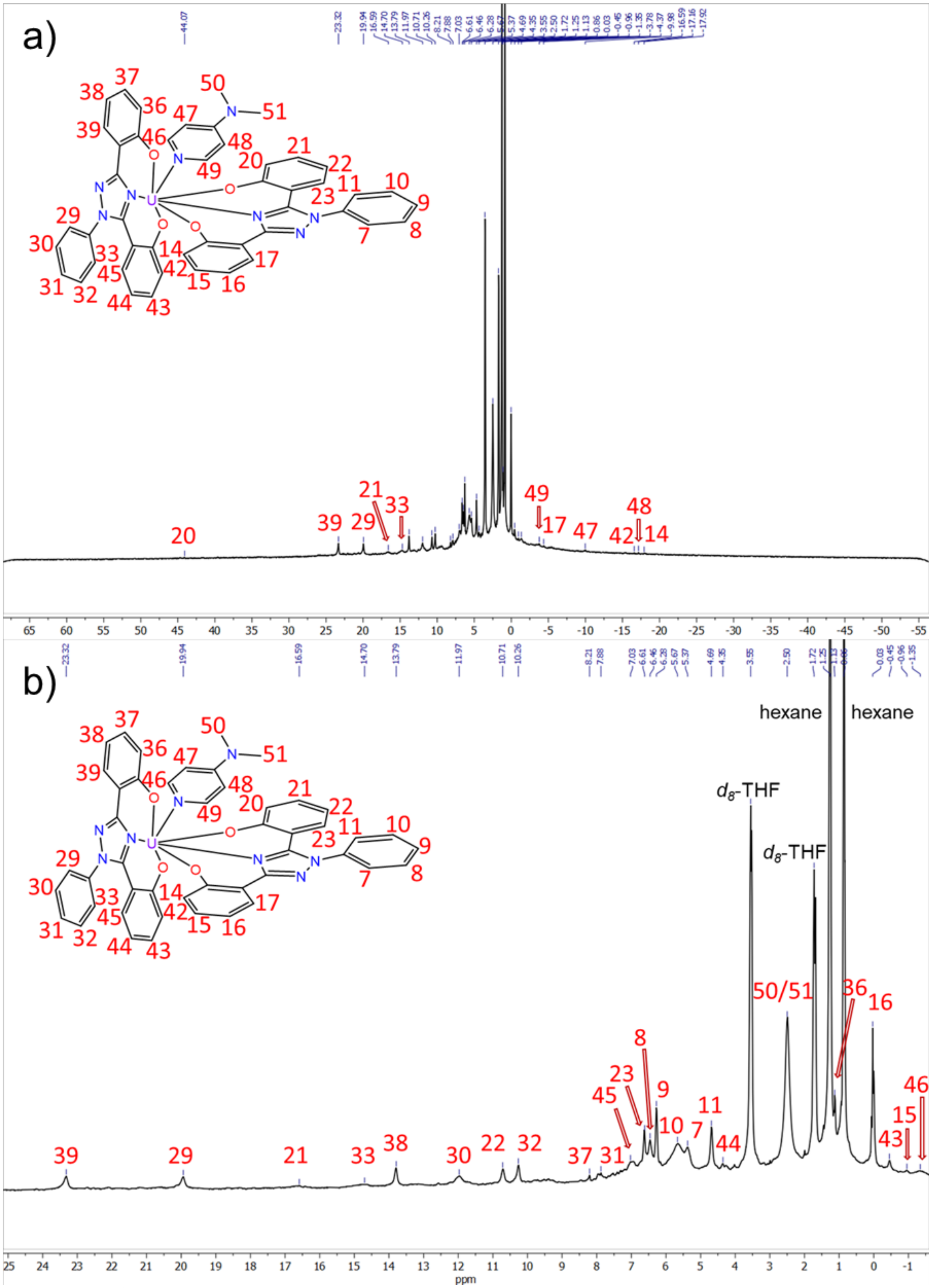

Figure S3. ^{1}H NMR spectrum (full (a) and partial (b)) recorded on a Bruker Avance Neo 400 Nano of **U(IV)ExPh** recorded in d_8-THF. All product and residual solvent peaks are labeled. Pseudo-contact

shift determinations were aided by Paramagpy Python script in conjunction with crystallographic data as described in the main text and detailed further below.

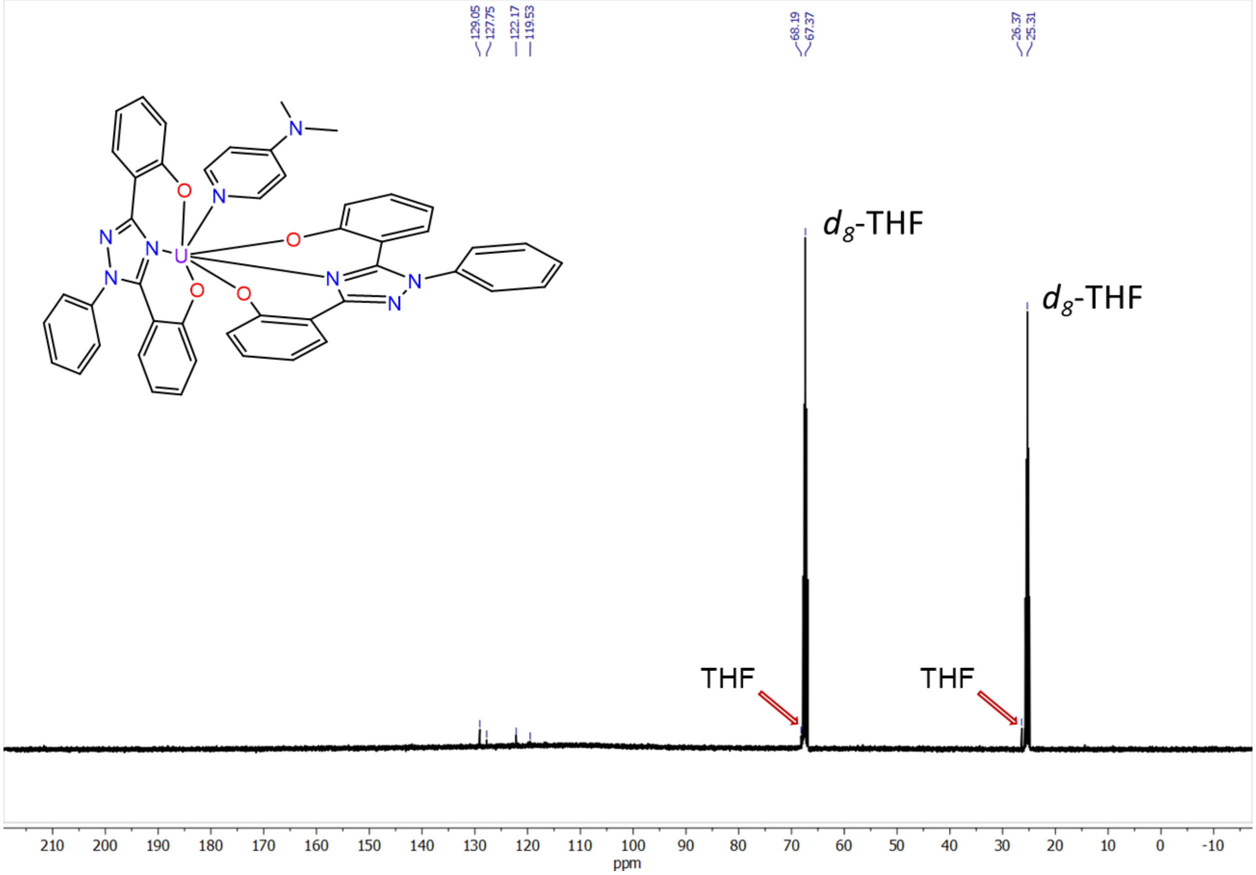

Figure S4. $^{13}C\{^1H\}$ NMR spectrum obtained on a Bruker Avance Neo 400 Nano of **U(IV)ExPh** recorded in d_8-THF. Peaks were indeterminable. The broadened baseline was due to the Teflon liner.

[U(V)ExPh$_2$(DMAP)]$_2^+$[BArF$_{24}$]$_2^-$:

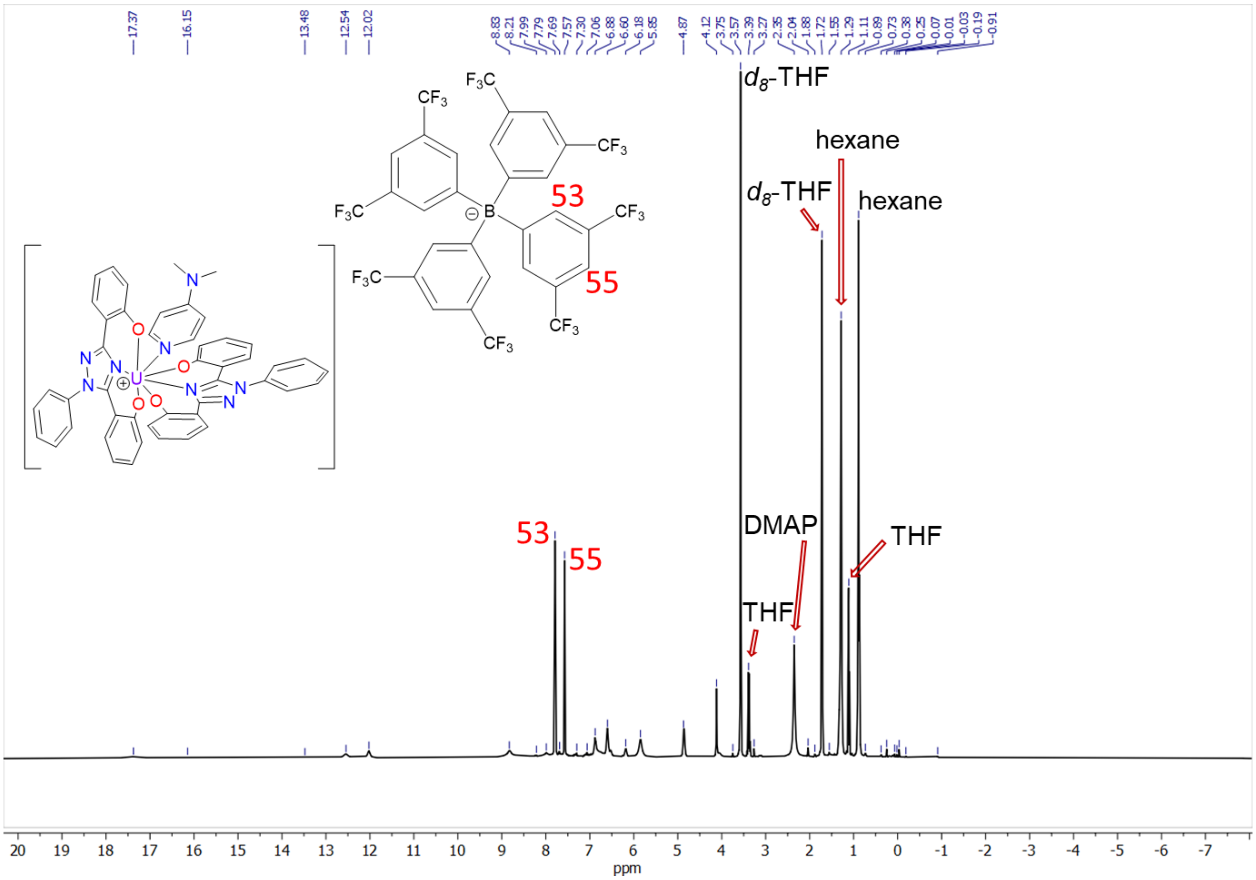

Figure S5. ^{1}H NMR spectrum of **U(V)ExPh** recorded in d_8-THF on a Bruker Avance Neo 400 Nano. DMAP, BArF$_{24}$, and solvent peaks are labeled. Paramagnetic shifting resulted in an increased number of peaks and complexity as compared to the pro-ligand and diamagnetic uranium-containing species. Due to a lack of suitable single crystals for XRD, full peak assignments could not be made.

$[U(III)ExPh_2(DMAP)]_2^-[K^+]_2$:

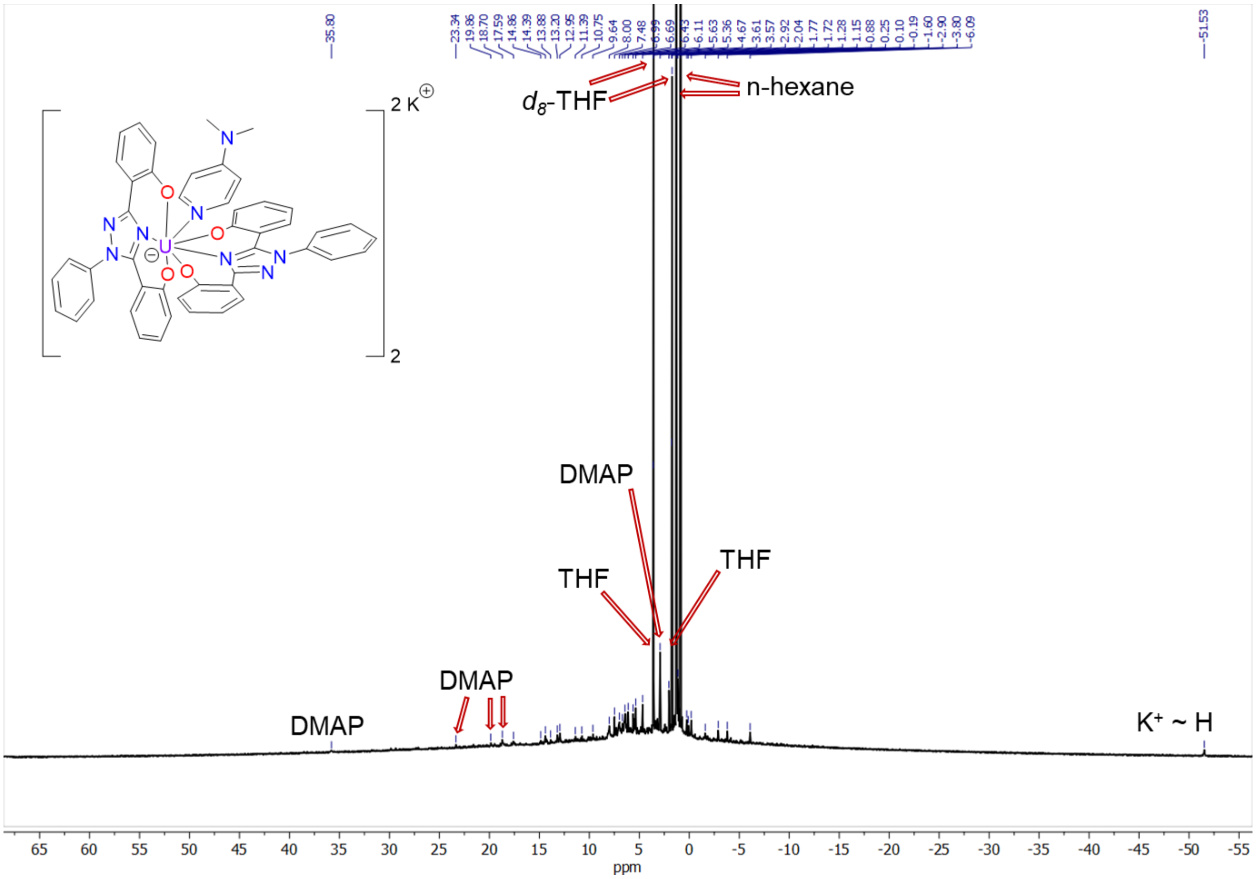

Figure S6. ^{1}H NMR spectrum obtained on a Bruker Avance Neo 400 Nano of the species referred to as **U(III)$_{\mathbf{red}}$ExPh** as recorded in d_8-THF. DMAP and solvent peaks are labeled. A highly negative shifted peak is assigned to a proton that is thought to reside near the counter potassium cation. Paramagnetic shifting resulted in an increased number of peaks and complexity as compared to the pro-ligand and diamagnetic uranium-containing species. No peak assignments corresponding to the product could be made in the present instance.

[U(III)ExPh(HMDS)]$_2$:

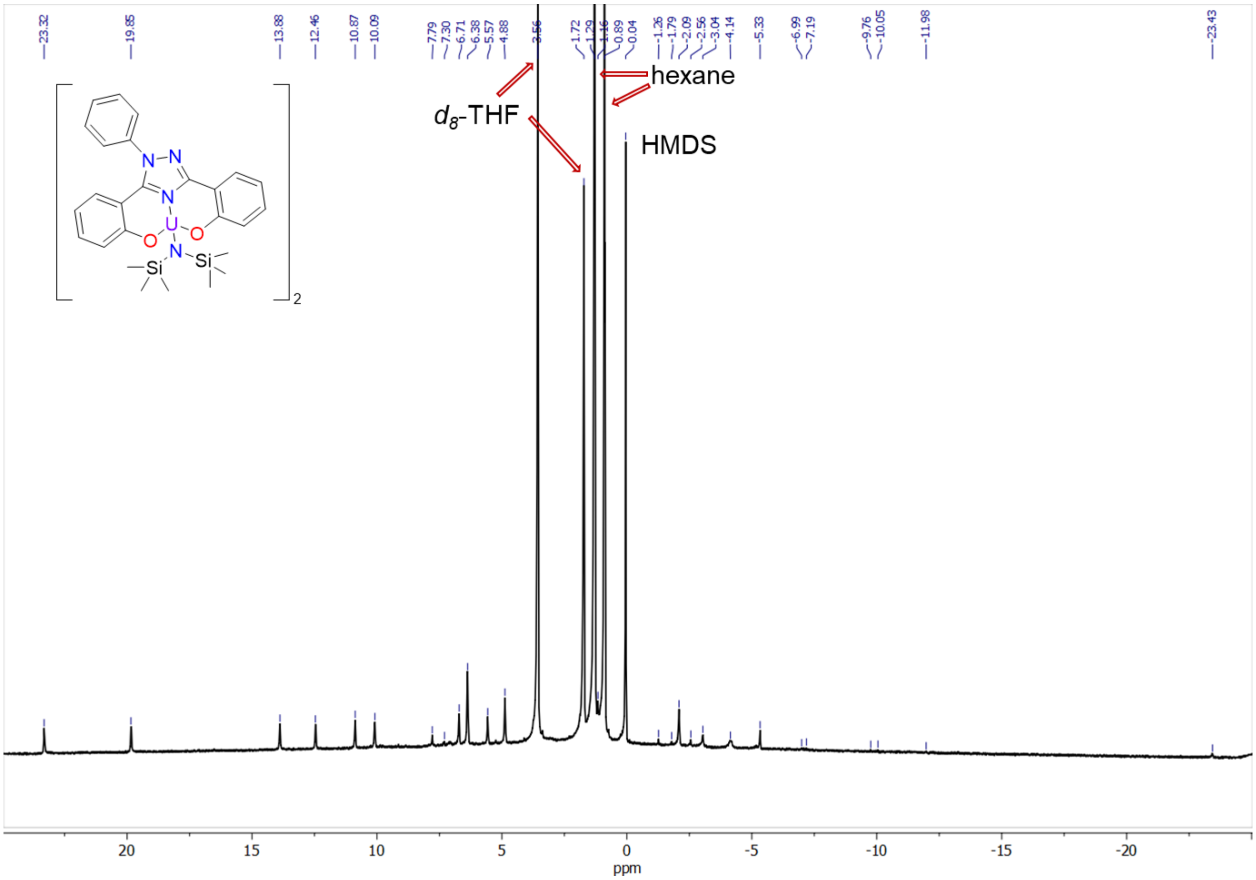

Figure S7. ^{1}H NMR spectrum obtained on a Bruker Avance Neo 400 Nano of the material referred to **U(III)$_{\mathbf{HMDS}}$ExPh** as recorded in d_8-THF. HMDS and solvent peaks labeled. Paramagnetic shifting resulted in an increased number of peaks and complexity as compared to the pro-ligand and diamagnetic uranium-containing species. No peak assignments corresponding to the product could be made in the present instance.

Comparison of the ^{1}H NMR spectral of [U(IV)ExPh$_2$(DMAP)]$_2$ and [U(V)ExPh$_2$(DMAP)]$_2^+$[BArF$_{24}$]$_2^-$:

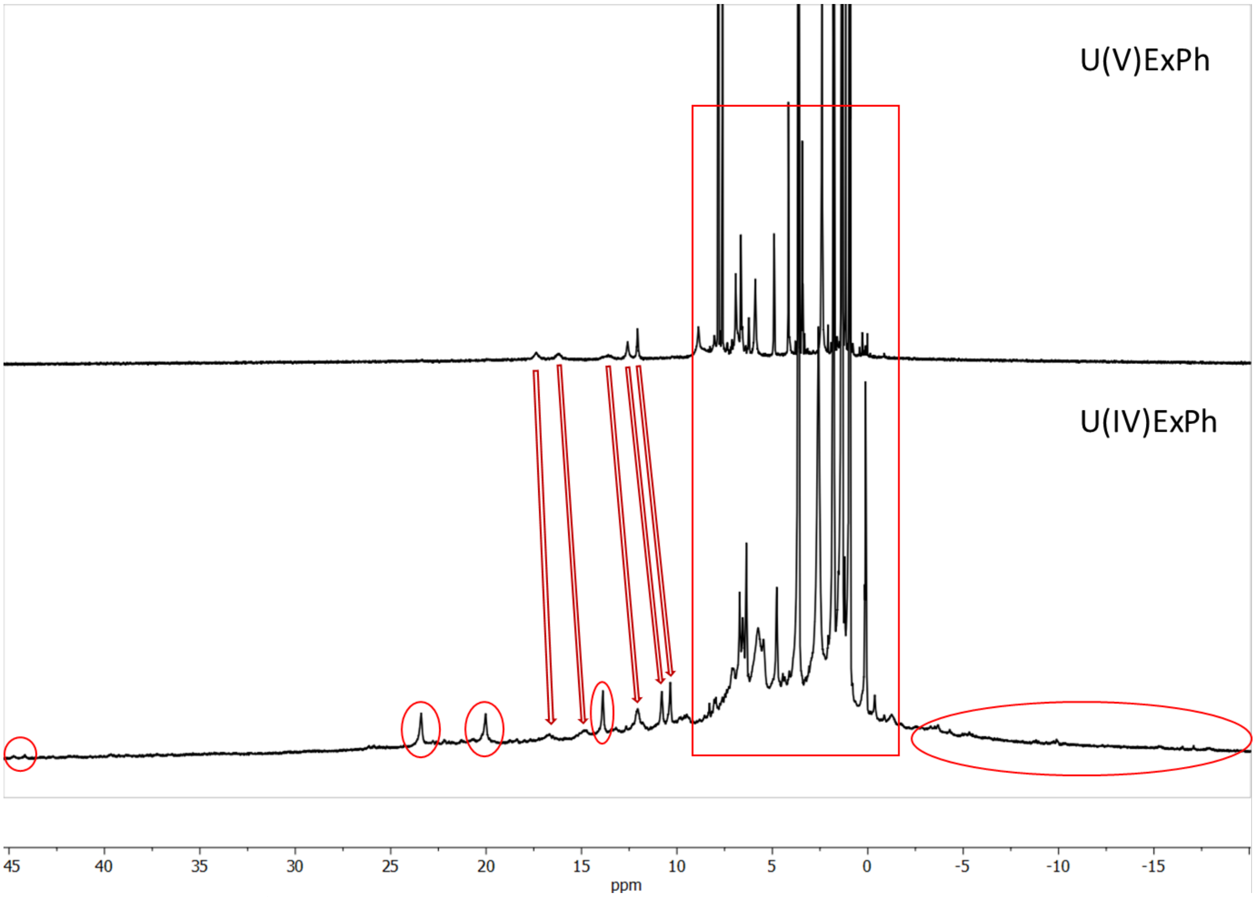

Figure S8. Stacked ^{1}H NMR spectra of **U(V)ExPh** (top) and **U(IV)ExPh** (bottom) obtained on a Bruker Avance Neo 400 Nano as recorded in *d*$_8$-THF.

The U(IV) complex showed more peaks at higher chemical shifts (red circles) owing to the increase in paramagnetism of U(IV), while the protons distant from the uranium centers mostly overlap in the aromatic region (red box). Some peaks showed a slight downfield shift in the case of the U(V) species as compared to **U(IV)ExPh**; this is ascribed to deshielding of the ligand by U(V) as compared to U(IV).

Comparison of the ^{1}H NMR spectral of [U(III)ExPh(HMDS)]$_2$, [U(IV)ExPh$_2$(DMAP)]$_2$, and[U(III)ExPh$_2$(DMAP)]$_2^-$[K$^+$]$_2$:

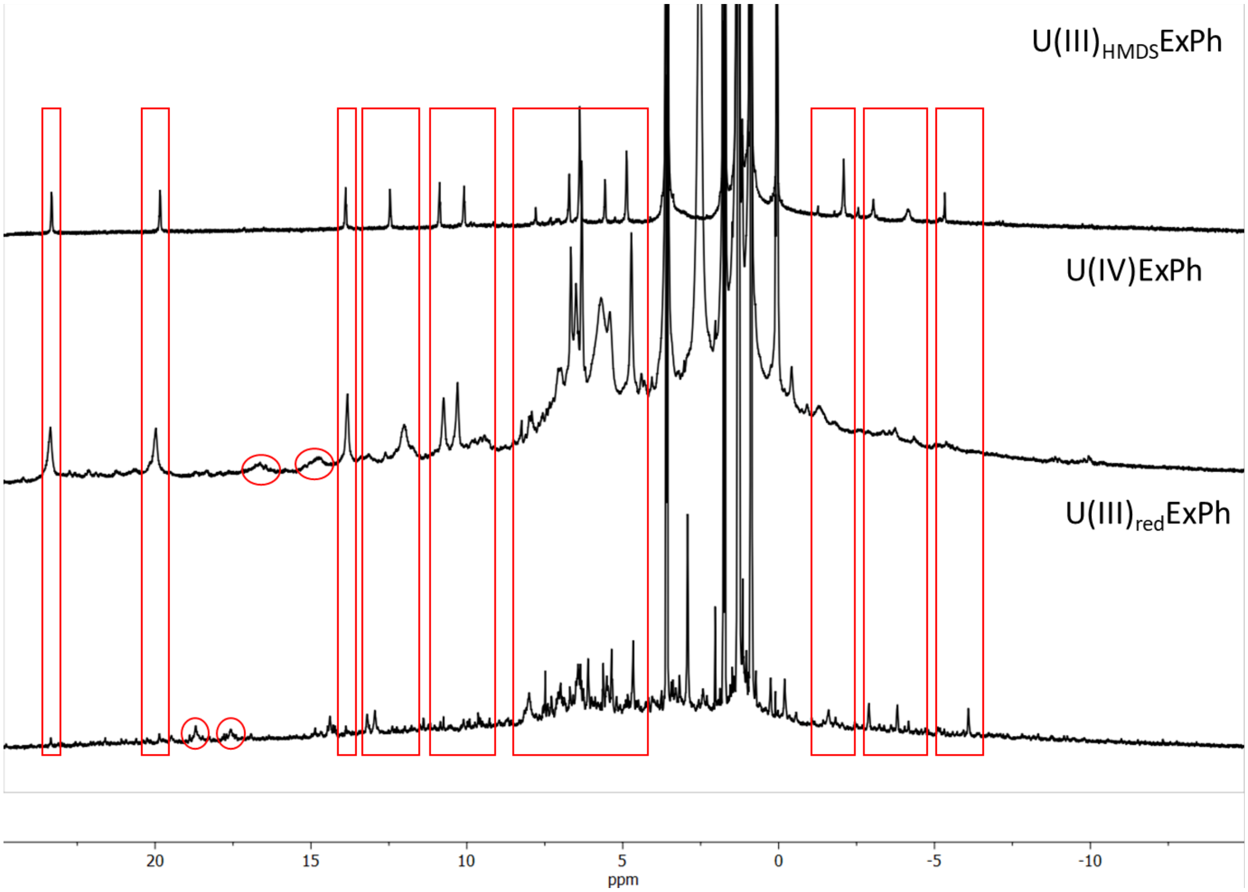

Figure S9. Stacked ^{1}H NMR spectra of **U(III)$_{\mathbf{HMDS}}$ExPh** (top), **U(IV)ExPh** (middle), and the species referred to as **U(III)$_{\mathbf{red}}$ExPh** (bottom) obtained on a Bruker Avance Neo 400 Nano as recorded in d_8-THF.

^{1}H NMR spectral studies provided additional support for the conclusion that the putative U(III) complex, if formed, was unstable within this ligand system and that conversion to a U(IV) species was occurring. The U(IV) complex showed nearly identical peaks within and without the typical aromatic region (red boxes). Some peaks showed a slight downfield shift from the known U(IV) (middle) complex upon preparation of the putative **U(III)$_{\mathbf{red}}$ExPh** complex (bottom); likely, this is due to the presence of nearby counter ions.

NMR Pseudo-Contact Shifts

In the DMAP adduct of **[U(IV)ExPh**, the ^{1}H signals experience large chemical shifts due to the presence of the two paramagnetic U(IV) metal centers. We assume that direct bonding Fermi Contact interactions are negligible and only through-space pseudo-contact chemical shifts (PCS) are present. Two distinct challenges exist that complicate peak assignments in this dimeric system. First, a list of assigned peaks from an isostructural diamagnetic complex does not exist which are typically required to initially calculate the PCS.[12,13] Second, as evidenced from the hexavalent uranyl complex, the aromatic ligands produce methine peaks that occur within a narrow chemical shift range of 6 to 8 ppm. Thus, little contrast exists to assign the diamagnetic shifts. Therefore, we designed a self-consistent fitting procedure that used only the chemical shifts of the paramagnetic complex and the crystal structure metric parameters.

The PCS is defined as the additional chemical shift imparted by through-space (dipolar) interactions with the anisotropic portion of the magnetic susceptibility tensor (**χ**) generated by the unpaired electrons of paramagnetic metal centers. This interaction depends on both the distance and relative orientation of the nuclear spin with respect to **χ**. The shielding tensor (**σ**) resulting from a single paramagnetic metal site is calculated as:[13]

$$\sigma = \frac{1}{4\pi r^5}\begin{bmatrix}(3x^2 - r^2) & 3xy & 3xz \\ 3xy & (3y^2 - r^2) & 3yz \\ 3xz & 3yz & (3z^2 - r^2)\end{bmatrix} \cdot \begin{bmatrix}\chi_{xx} & \chi_{xy} & \chi_{xz} \\ \chi_{xy} & \chi_{yy} & \chi_{yz} \\ \chi_{xz} & \chi_{yz} & \chi_{zz}\end{bmatrix}$$

Where r is the distance from the metal center and x, y, and z are the relative position of the nucleus with respect to the metal center. The traces of **σ** and **χ** are the isotropic chemical shift (δ_{iso}) and isotropic magnetic susceptibility (χ_{iso}), respectively. Numerous fitting procedures have been developed to either fit the internuclear distances or the location and orientation of **χ** considering known values of the primary components of the anisotropic magnetic susceptibility tensor (**Δχ**) or prior chemical shift assignments. None of these values are known in this case, so an alternative procedure was developed to simultaneously assign the chemical shifts and determine the **χ**-tensor.

The routines were written in Python 3 and modified previous code implemented in the Paramagpy software.[13] The fitting routine first reads in the U(IV) and ^{1}H positions from the crystal structure. An identical value, from the principal components of the χ-tensor, was generated for both U(IV) sites but with randomly assigned Euler angles defining their relative rotations with respect to the crystal axes. This assignment assumes that these sites have the same magnetic susceptibility but with independent orientations. The principal components of the **χ**-tensor were constrained to be positive valued over the range of [0 to 10^{-32} m^3] with $\chi_{xx} < \chi_{yy} < \chi_{zz}$, and the Euler angles in the range of [0, π] using the same ZYZ convention as Paramagpy. The fitting procedure tracks and calculates the average chemical shift from both U(IV) centers for each ^{1}H site. The calculated and experimental chemical shifts are ranked from smallest to largest and the root mean square error is minimized. Since we have no prior knowledge of diamagnetic resonances, we assumed the diamagnetic chemical shifts for each site to be equivalent to those predicted by the MNova chemical shift prediction software. However, we acknowledge that differences due to the structure and binding of the complex would likely modify these initial signals. To accommodate these discrepancies in the model, the diamagnetic shifts were each perturbed by a random value of ±0.5 ppm at each iteration of the minimization routine. In this model, the methyl protons from the DMAP were excluded since they are well assigned, and rapid rotational averaging of the chemical shift is expected, although not accommodated by the model.

This model produces nine independent variables for the fitting routine and is highly susceptible to converging on local minima depending on the initial values. Therefore, a Basin-hopping routine included in the scipy optimize package was used to find the global minimum. Basin-hopping is a stochastic method that finds the global minimum by iteratively performing local minimization routines with randomly perturbed initial guesses based on prior minimization steps.[14] In practice, the Powell minimization method performed best when the guesses were far from the global minimum. The model was run several times with different initial guesses for the parameters to ensure that the final result was indeed the global minimum (**Figure S10**).

The results for the fitting routine are presented in **Table S1**. We note that χ_{iso} determined from the fitting routine is nearly identical to that derived from the magnetic susceptibility measurements despite the few constraints in the model.

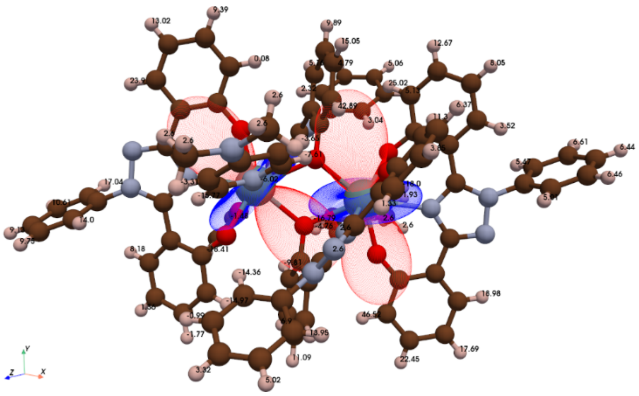

Figure S10. Result of a model that overlays the $\Delta\chi$ U(IV) tensors on the molecular structure with the individual calculated chemical shifts for each ^{1}H site. The red and blue lobes represent the positive and negative portions of the $\Delta\chi$ tensor, respectively.

Table S1. Experimental vs. Calculated Pseudo-Contact Shifts for **U(IV)ExPh**

	DMAP	DMAP	DMAP	DMAP	DMAP	DMAP	DMAP	DMAP	DMAP	DMAP			
**Experimental ppm**	-3.78	-1.35	-17.16	-9.98	2.50	2.50	2.50	2.50	2.50	2.50			
**Calculated / ppm**	-3.65	-3.31	-15.77	-7.61	2.50	2.50	2.50	2.50	2.50	2.50			
	Bridge	Bridge	Bridge	Bridge	Bridge	Bridge	Bridge	Bridge	Bridge	Bridge	Bridge	Bridge	Bridge
**Experimental ppm**	4.69	5.37	5.67	6.46	6.28	-17.92	-0.96	0.03	-4.37	44.07	16.59	10.71	6.61
**Calculated / ppm**	2.32	3.04	4.79	5.13	5.06	-18.41	-2.68	-1.48	-6.02	42.89	15.05	9.89	5.16
	Non-bridge	Non-bridge	Non-bridge	Non-bridge	Non-bridge	Non-bridge	Non-bridge	Non-bridge	Non-bridge	Non-bridge	Non-bridge	Non-bridge	Non-bridge
**Experimental ppm**	14.7	19.94	10.26	11.97	7.88	1.13	8.21	13.79	23.32	-16.59	-0.45	4.35	7.03
**Calculated / ppm**	14.00	17.04	9.75	10.61	9.13	0.08	9.39	13.02	23.90	-14.97	-1.77	1.36	8.18

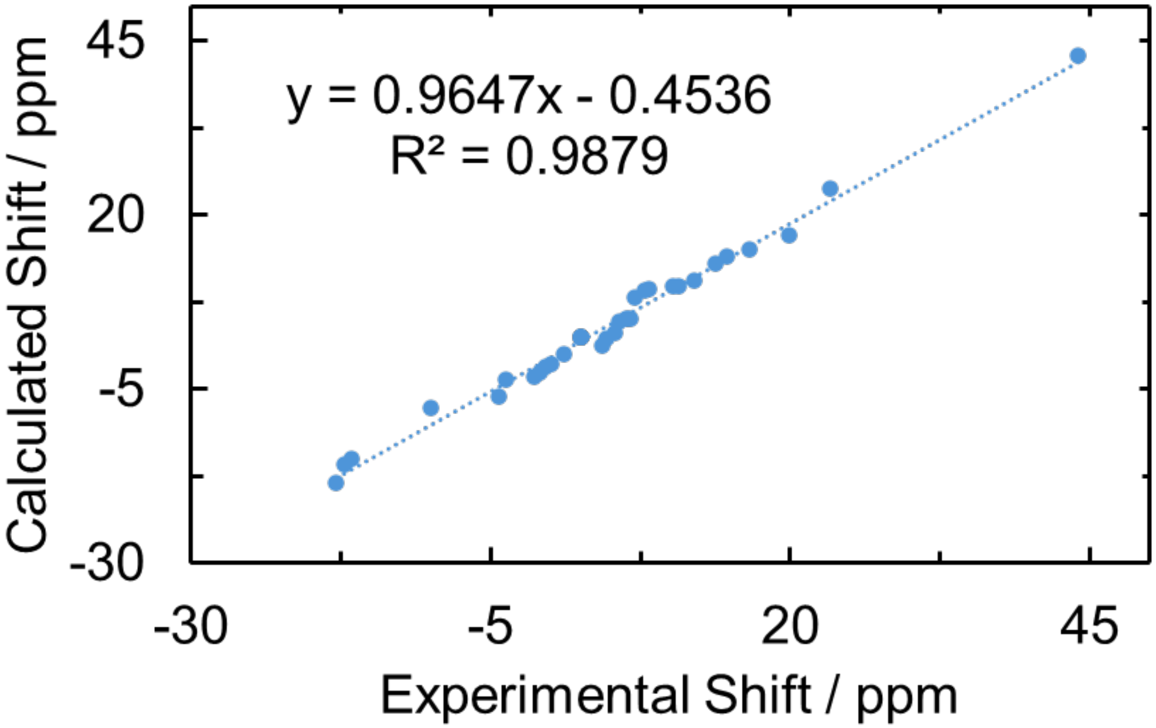

Figure S11. Linear trend of the experimental versus calculated ^{1}H chemical shifts for **U(IV)ExPh**.

UV-Vis Spectra

$[(UO_2ExPh)_3OH]^-[(HTEA)]^+$:

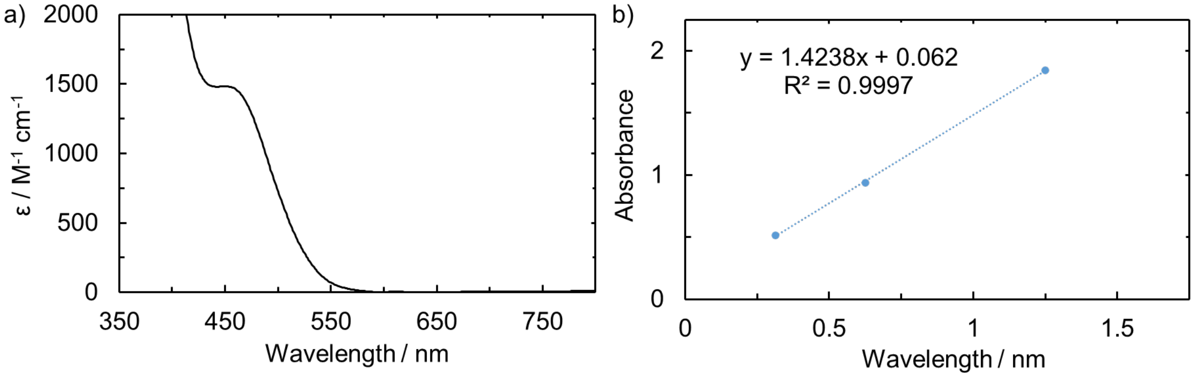

Figure S12. Averaged (n = 3) a) UV-vis spectrum and b) Beer-Lambert plot of **UO_2ExPh** measured in DMSO at room temperature. A shoulder is observed at 456 nm.

[U(IV)ExPh$_2$(DMAP)]$_2$:

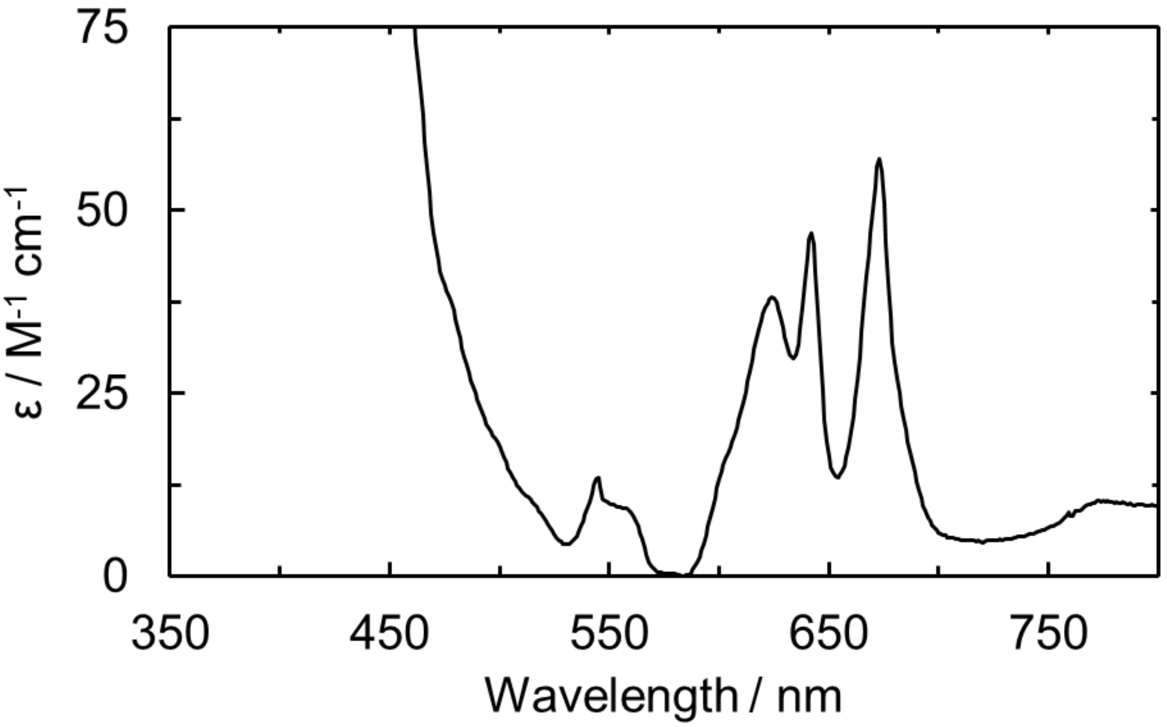

Figure S13. Averaged (n = 3) UV-vis spectrum of **U(IV)ExPh** measured in THF at room temperature. Peaks observed for *f-f* transitions at 674, 642, 625, and 545 nm. The shoulder observed at 562 nm proved non-linear with concentration and is likely below detection limits.

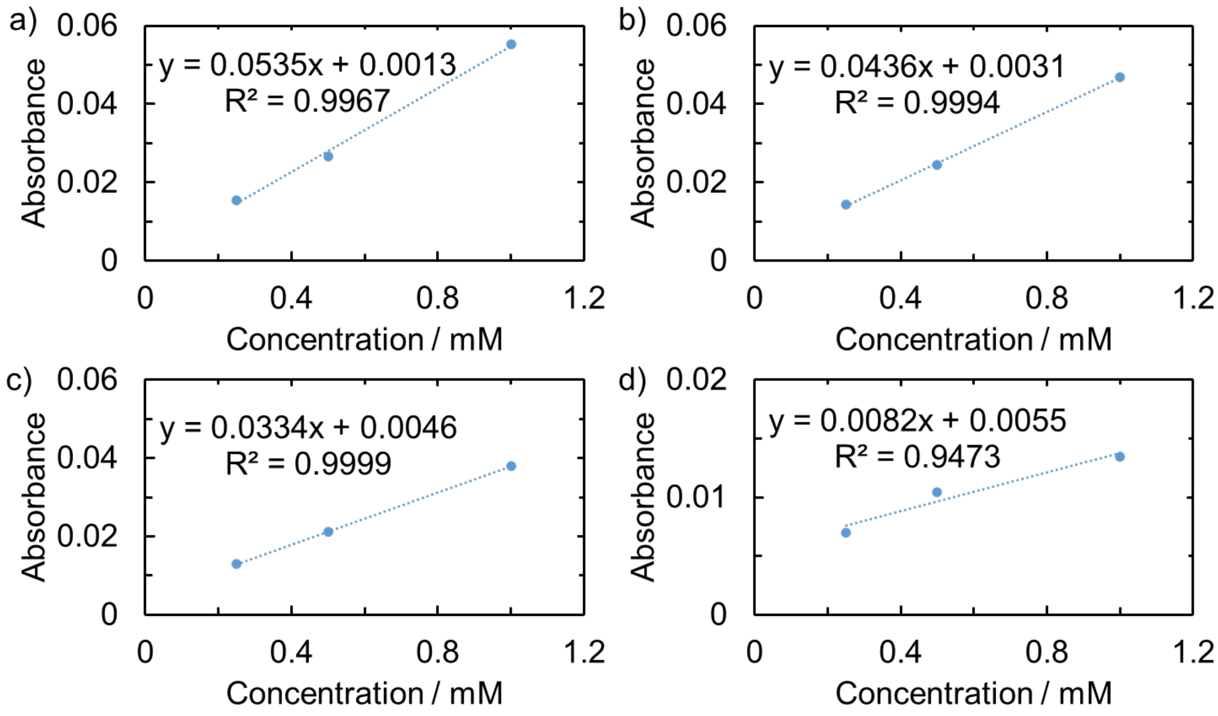

Figure S14. Averaged (n = 3) Beer-Lambert plots for **U(IV)ExPh** recorded in THF at room temperature. Peaks observed for *f-f* transitions at a) 674, b) 642, c) 625 nm, and d) 562 nm.

$[U(V)ExPh_2(DMAP)]_2^{+}[BArF_{24}]_2^{-}$:

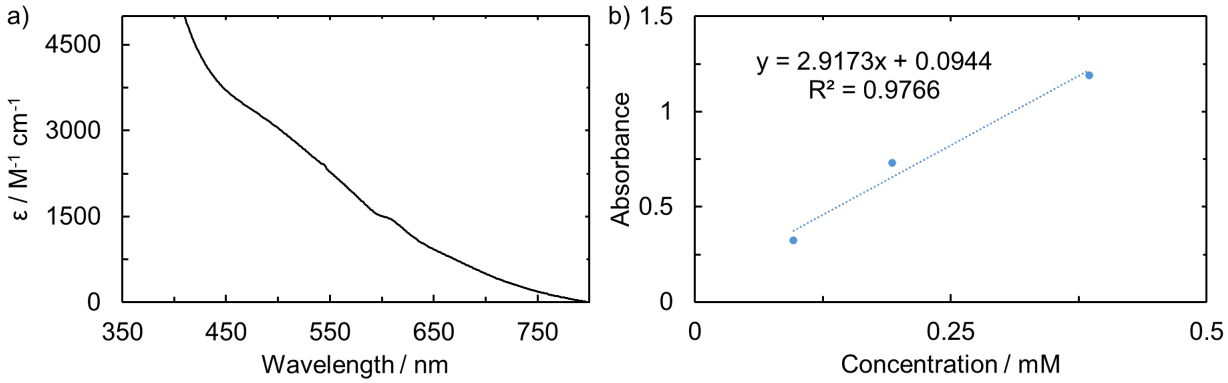

Figure S15. Averaged (n = 3) a) UV-vis spectrum and b) Beer-Lambert plot of **U(V)ExPh** measured in fluorobenzene at room temperature. A shoulder is observed at 474 nm.

Further elucidation of the electronic structure of the U(V) complex was performed using near infrared (NIR) spectroscopy. A sharp transition was noted at 1444 nm consistent with a $5f^1$ system (see main text).

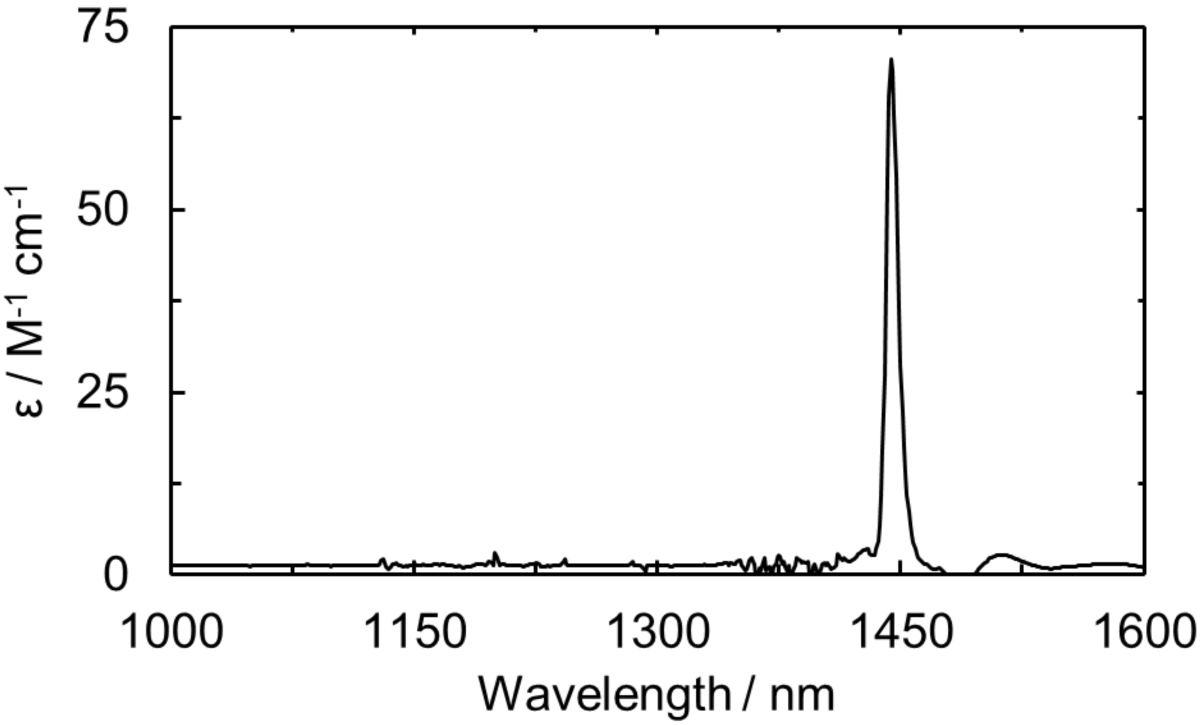

Figure S16. Near infrared spectrum of $[(U(V)ExPh_2DMAP)^{+}]_2[BArF_{24}^{-}]_2$ measured in tetrahydrofuran at room temperature. A sharp transition is seen at 1444 nm.

Material referred to as [U(III)ExPh(HMDS)]$_2$:

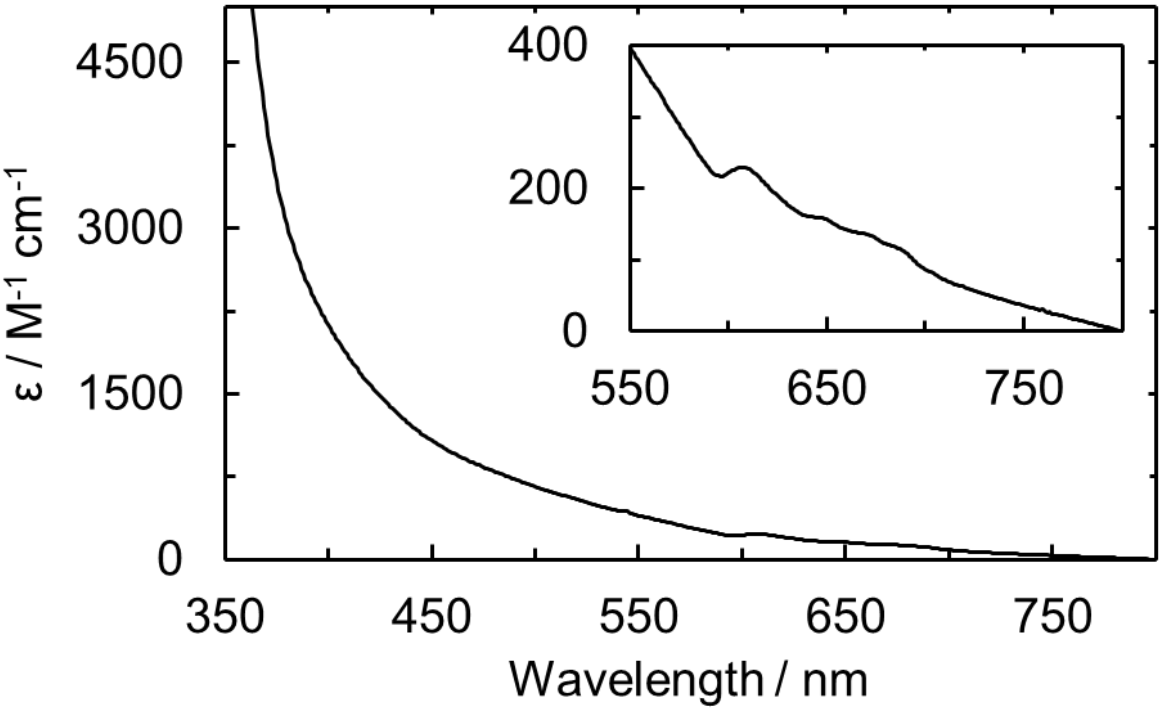

Figure S17. Averaged (n = 3) UV-vis spectrum of the species referred to as **U(III)$_{HMDS}$ExPh** recorded in fluorobenzene at room temperature. Peak and shoulder observed at 607 and 674 nm, respectively, were non-linear in absorbance and likely below detection limits. The in-set shows a zoomed-in region between 550 and 800 nm.

The electronic properties probed through UV-vis spectroscopy provided further support for the conclusion that the putative U(III) complex is unstable and that the metal center converts to the corresponding U(IV) species. Unlike typical U(III) complexes with strong ligand field interactions that give rise to deep colors, the UV-vis spectrum showed minimal absorption peaks; in that respect the spectrum resembles that of the U(IV) analogue.

Infrared Transmittance Spectra

H_2ExPh:

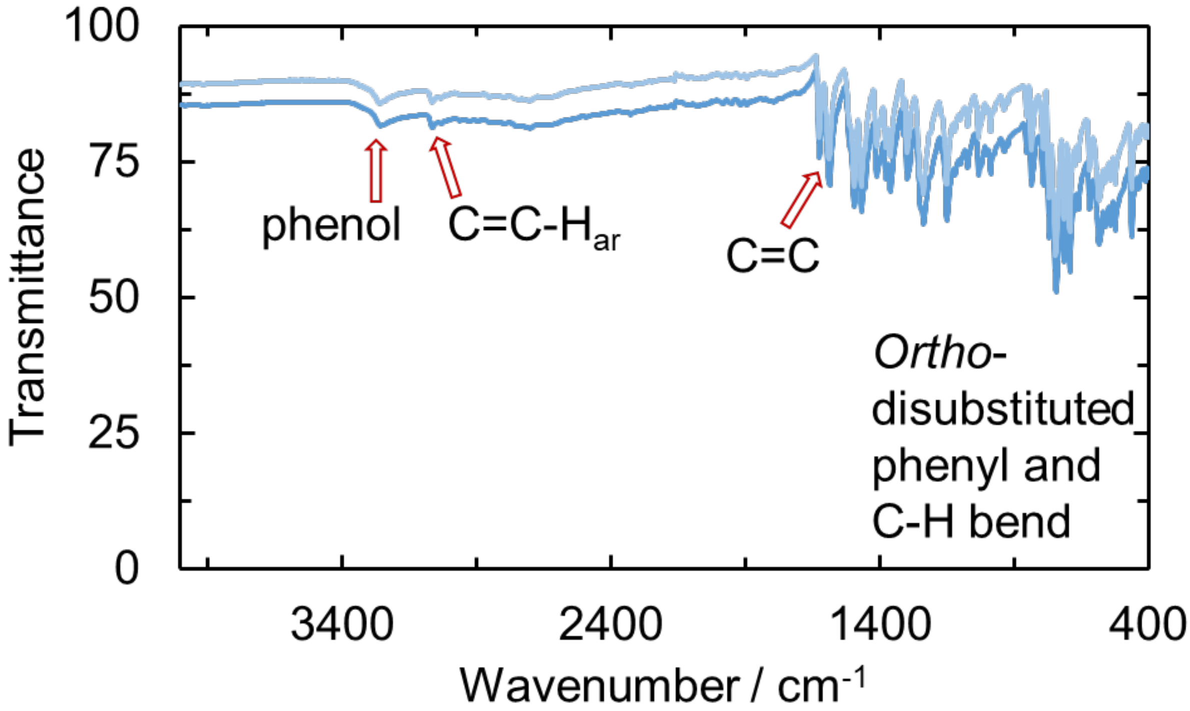

Figure S18. IR transmittance spectra of the pro-ligand **H_2ExPh** obtained from crystalline material. Phenol, aromatic carbon, and C(sp^2)-H peaks were identified. The phenyl vibrations and C-H bending modes lie in the fingerprint region.

U ExPh IR Series:

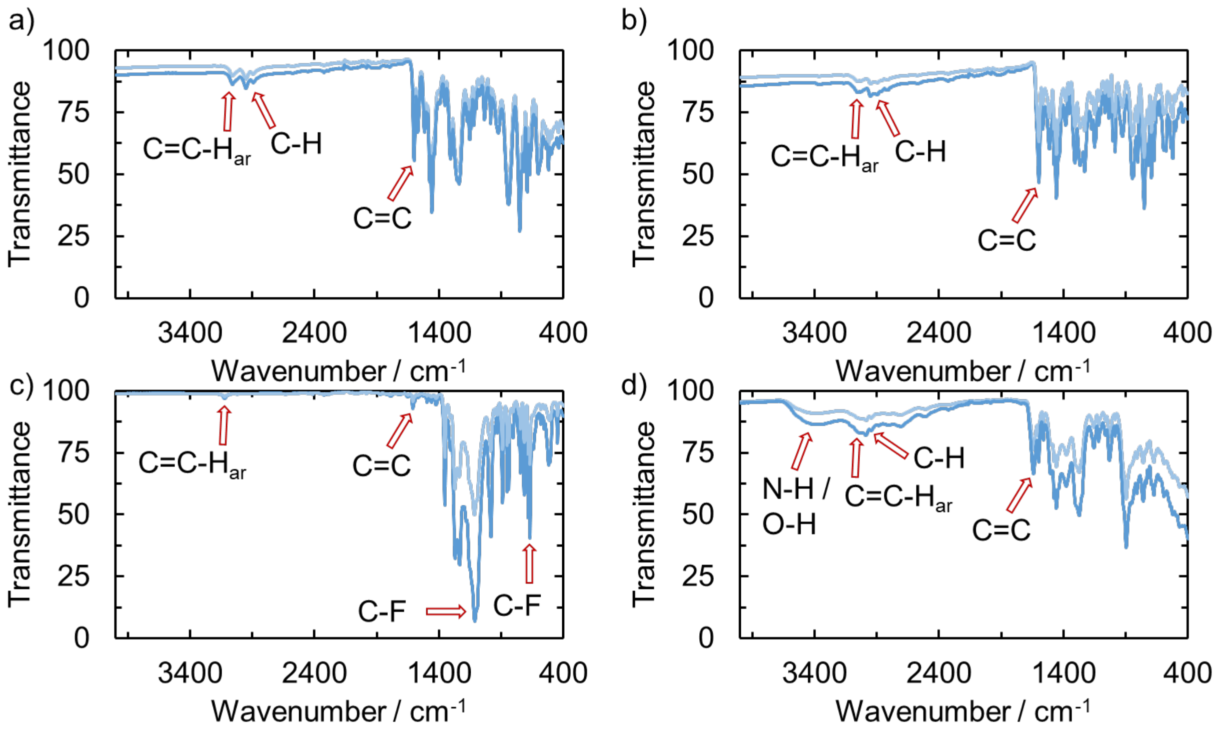

Figure S19. IR transmittance measurements of a) the species referred to as **U(III)$_{\mathbf{HMDS}}$ExPh**, b) **U(IV)ExPh**, c) **U(V)ExPh**, and d) **$\mathbf{UO_2}$ExPh** obtained from crystalline material. Aromatic carbon, C(sp^2)-H, C(sp^3)-H, ammonium, and BArF$_{24}$ peaks are identified where appropriate.

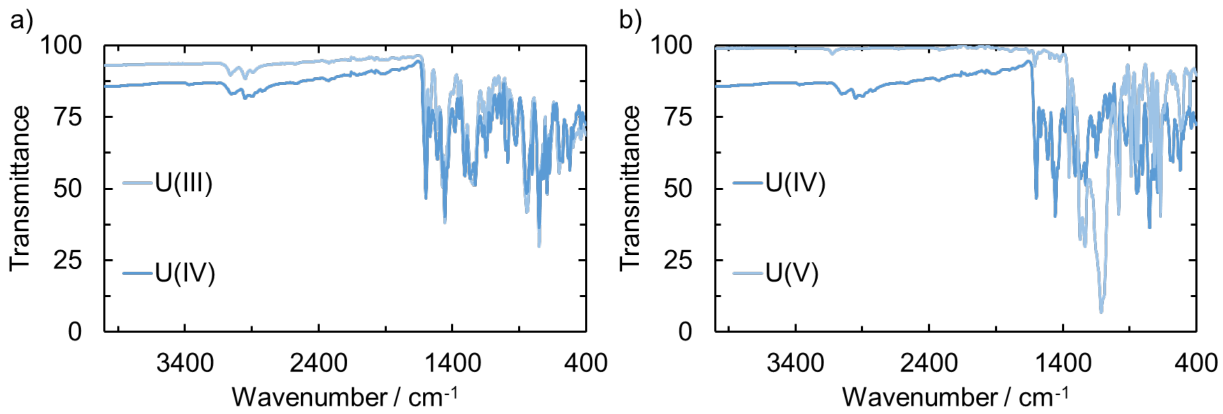

Figure S20. IR transmittance measurements of a) the species referred to as **U(III)$_{\mathbf{HMDS}}$ExPh**, **U(IV)ExPh** and b) **U(IV)ExPh** and **U(V)ExPh** obtained from crystalline material. Overlays highlight the differences in the spectra for the species in question.

The IR transmittance spectra of the putative U(III) and structurally characterized U(IV) complexes provided another data set showcasing the similarities in the species. The HMDS peaks are mostly hidden in the fingerprint region, although slight decreases in transmittance of the putative U(III) species at ~850 cm^{-1} due to Si-N stretching and a decrease at ~450 cm^{-1} due to Si-N breathing are visible. The data highlights the fact that there are no major differences in the complexes. For **U(V)ExPh**, the BArF$_{24}$ peaks are visible at 1110 cm^{-1} and 670 cm^{-1}.

Solid-State Absorbance Spectra

$[(UO_2ExPh)_3OH]^-[(HTEA)]^+$:

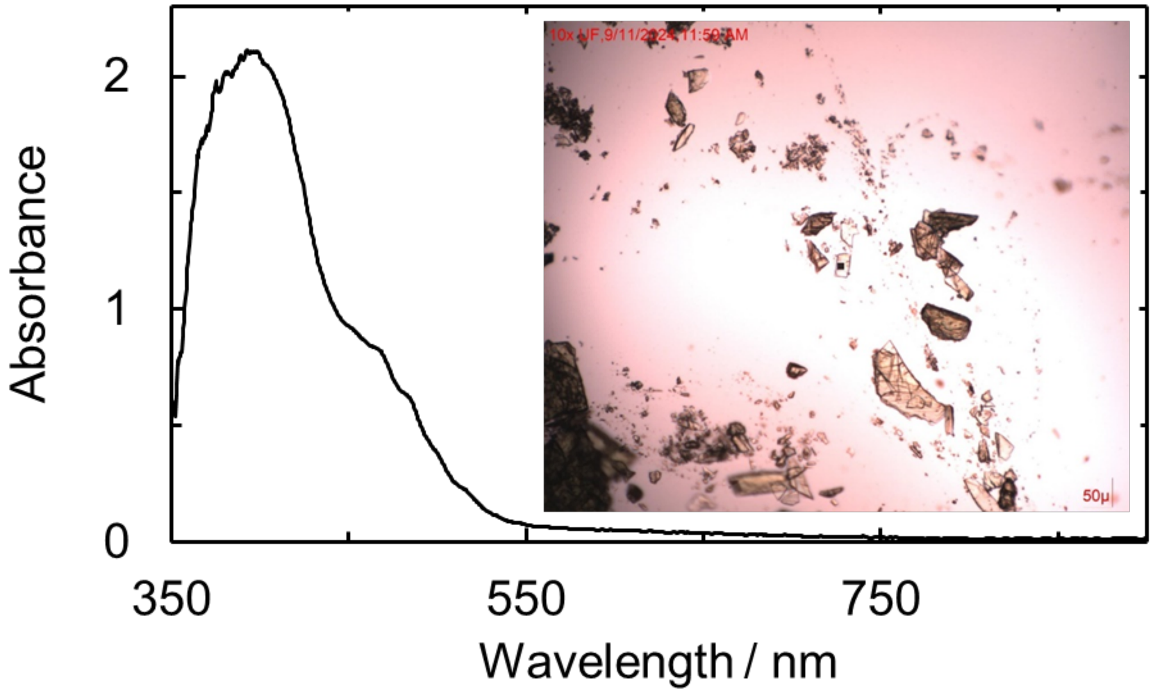

Figure S21. Solid-state absorbance spectrum, and overlaid image, of **UO_2ExPh** crystals obtained on a Craic QDI 2010 UV-Vis/NIR instrument from 350 – 900 nm. The recorded spectrum complemented the peak seen in the solution-state UV-Vis spectral measurements. The crystals of this complex are yellow-orange plates. The image reveals several fractures ascribed to de-solvation.

[U(IV)ExPh$_2$(DMAP)]$_2$:

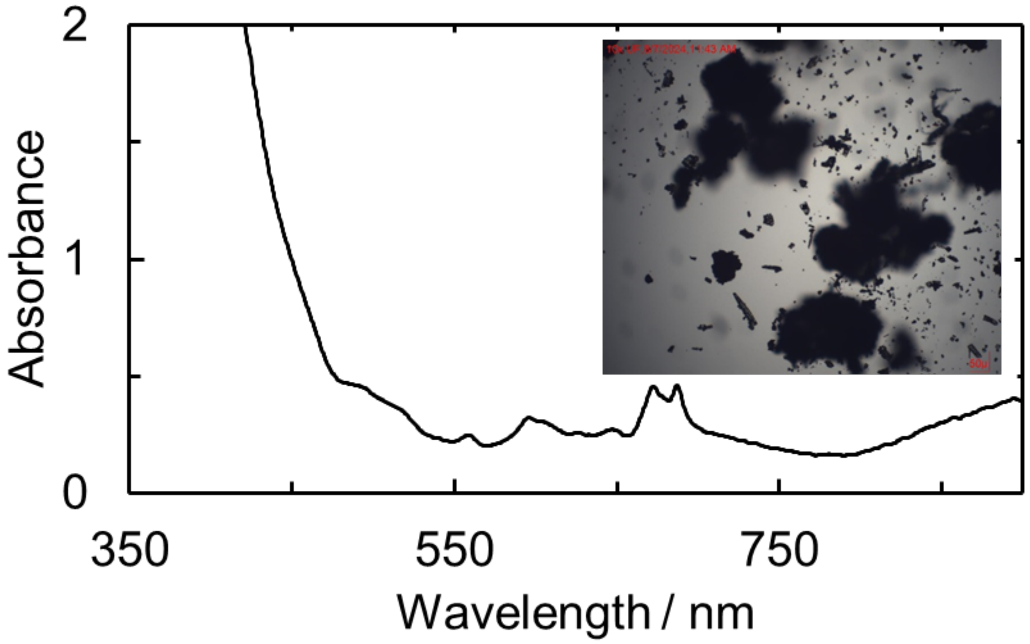

Figure S22. Solid-state absorbance spectrum, and overlaid image, of **U(IV)ExPh** crystals obtained on a Craic QDI 2010 UV-Vis/NIR instrument from 350 – 900 nm. The spectrum of crystals was characterized by peaks complementing those seen in the solution-state UV-Vis measurements.

$[U(V)ExPh_2(DMAP)]_2^+[BArF_{24}]_2^-$:

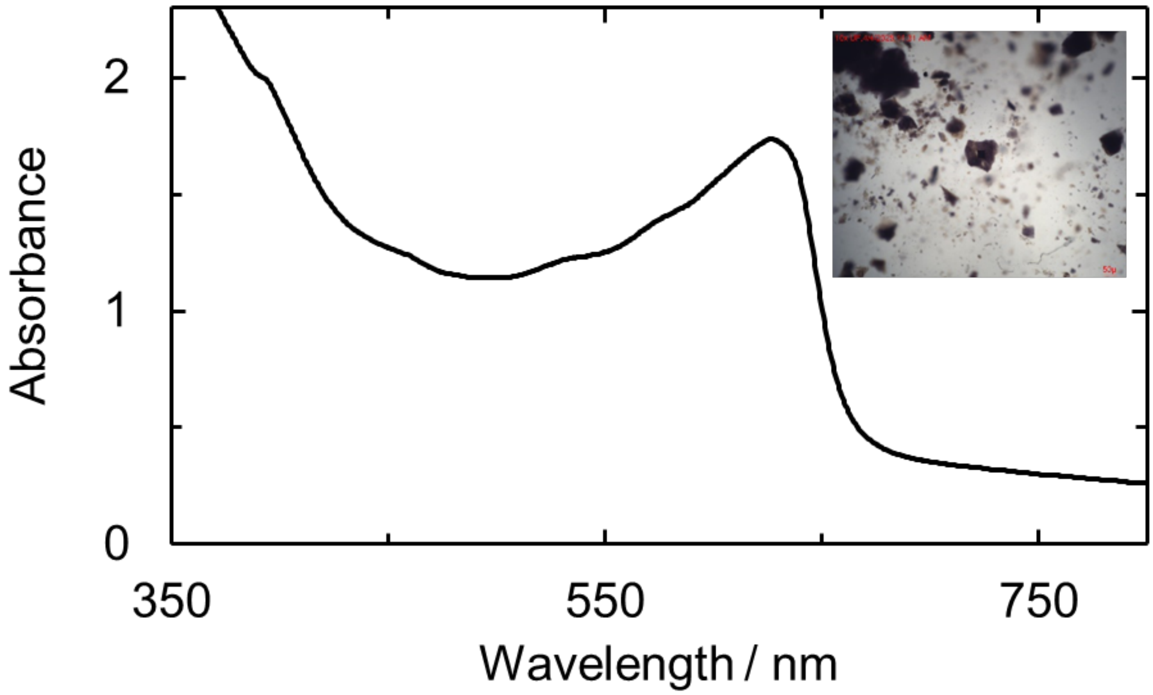

Figure S23. Solid-state absorbance spectrum, and overlaid image, of **U(V)ExPh** crystals recorded on a Craic QDI 2010 UV-Vis/NIR instrument from 350 – 900 nm. The spectrum of the crystals was characterized by broad and minimally red-shifted peaks; however, the recorded spectrum complemented that seen in the solution-state UV-Vis measurements.

Electrochemical Studies

Electrochemical measurements were performed at an analyte concentration of either 1 or 5 mM, depending on solubility, in THF containing 100 mM tetrabutylammonium hexafluorophosphate ($TBAPF_6$) as the supporting electrolyte. A glassy carbon working, Pt wire counter, and silver wire pseudo-reference electrode were used. Due to the insolubility of the U(IV) species, **U(IV)ExPh**, electrochemical measurements were performed immediately upon dissolution. Potential measurements were recorded in reference to the ferrocene/ferrocenium internal standard couple or decamethylferrocene/decamethylferrocenium, where appropriate, and determined through differential pulse voltammetry (DPV). Open-circuit potential (OCP) and cyclic voltammetry (CV) studies were performed initially without ferrocene and subsequently after the addition of ferrocene. Changes in the oxidative scan were noted upon the addition of ferrocene to the system as discussed in the main text. DPV was measured for the system yielding higher resolution of the ferrocene peak.

Electrochemical measurements of the supporting electrolyte, $TBAPF_6$, were made to ensure purity and of the pro-ligand, H_2ExPh, as phenols have a known tendency to undergo electrochemical oxidation. These cyclic voltammogram measurements revealed no redox peaks within the electrochemical window.

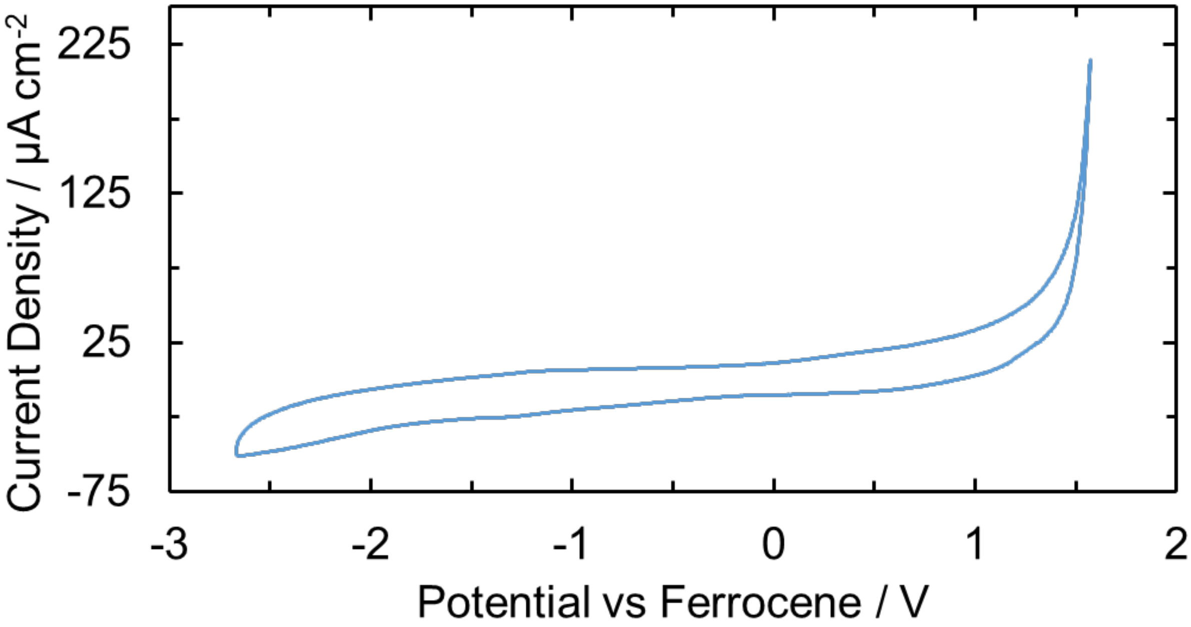

Figure S24. Cyclic voltammogram of $TBAPF_6$ at a concentration of 100 mM in THF at a scan rate of 100 mV/s. No peaks were seen within the electrochemical window.

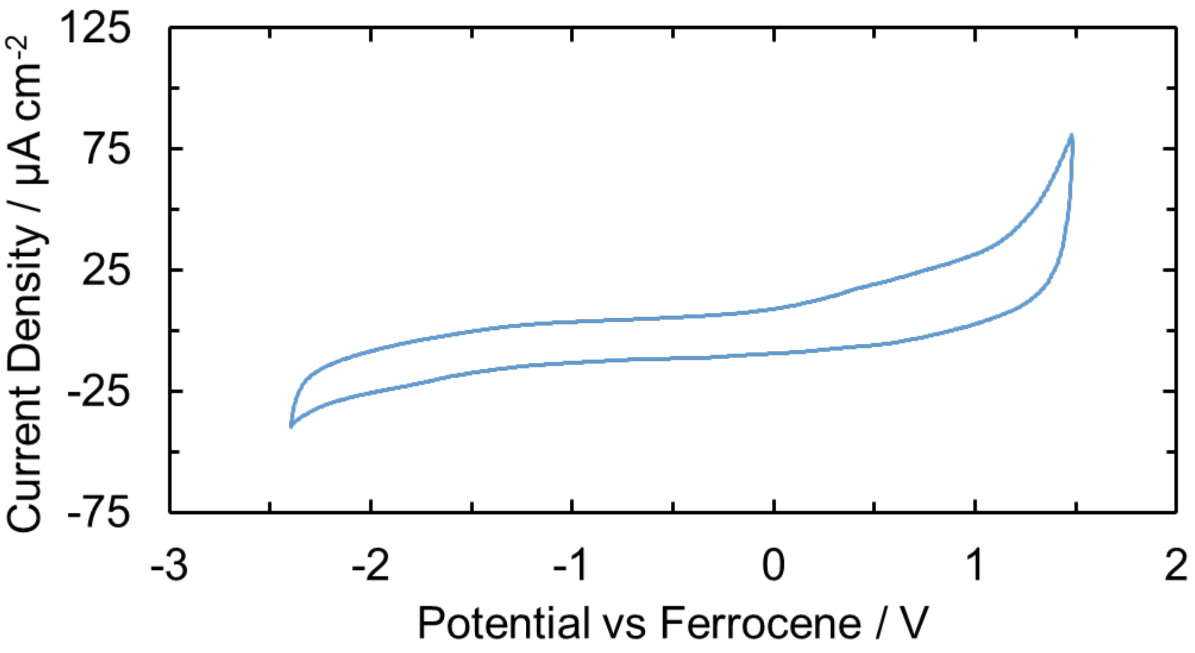

Figure S25. Cyclic voltammogram of H_2ExPh at a concentration of 5 mM in THF containing 100 mM $TBAPF_6$ at a scan rate of 100 mV/s. No peaks were seen within the electrochemical window.

Ferrocene as an internal reference was also added to the electrolyte/pro-ligand solution as a suitable control to ensure appropriate reversibility. No evidence of chemical processes occurring was seen.

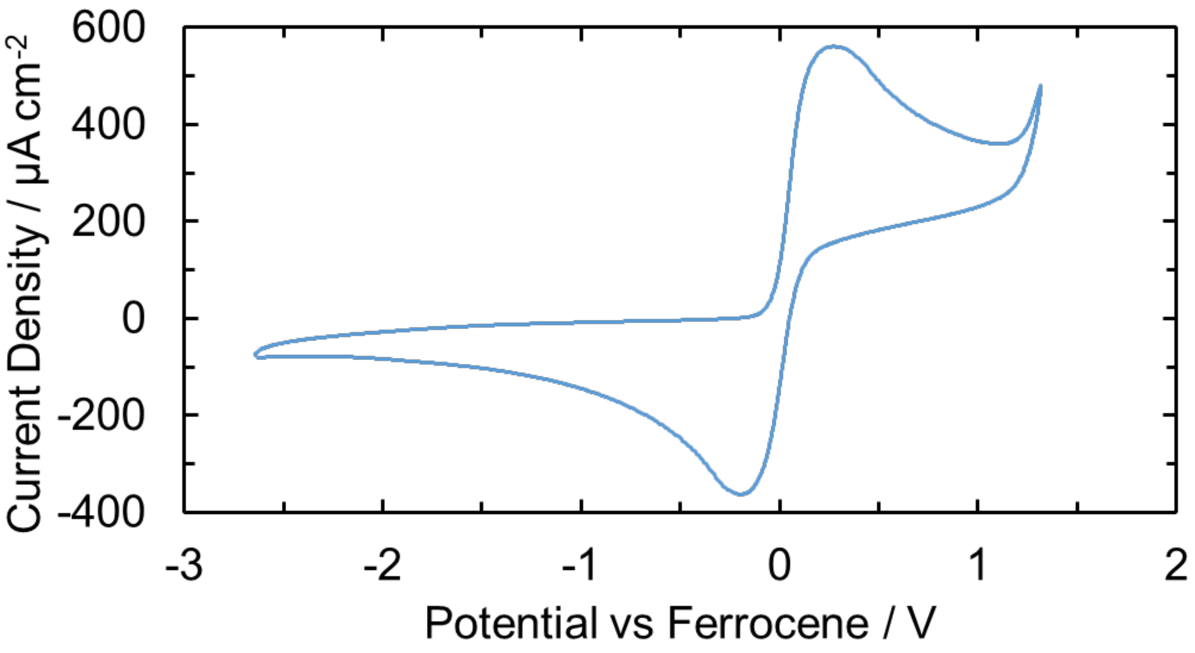

Figure S26. Cyclic voltammogram of H_2ExPh at a concentration of 5 mM in THF containing 5 mM of ferrocene and 100 mM $TBAPF_6$ at a scan rate of 100 mV/s. The forward and reverse peak of ferrocene can be seen.

The OCP value for the U(IV) chelate was a negative potential. This was attributed to the electron donating ability of the two multidentate **ExPh** ligands and a less Lewis acidic metal center (U^{4+} compared to U^{6+}) which cathodically shifted the OCP to a value of ~ -1.2 V.

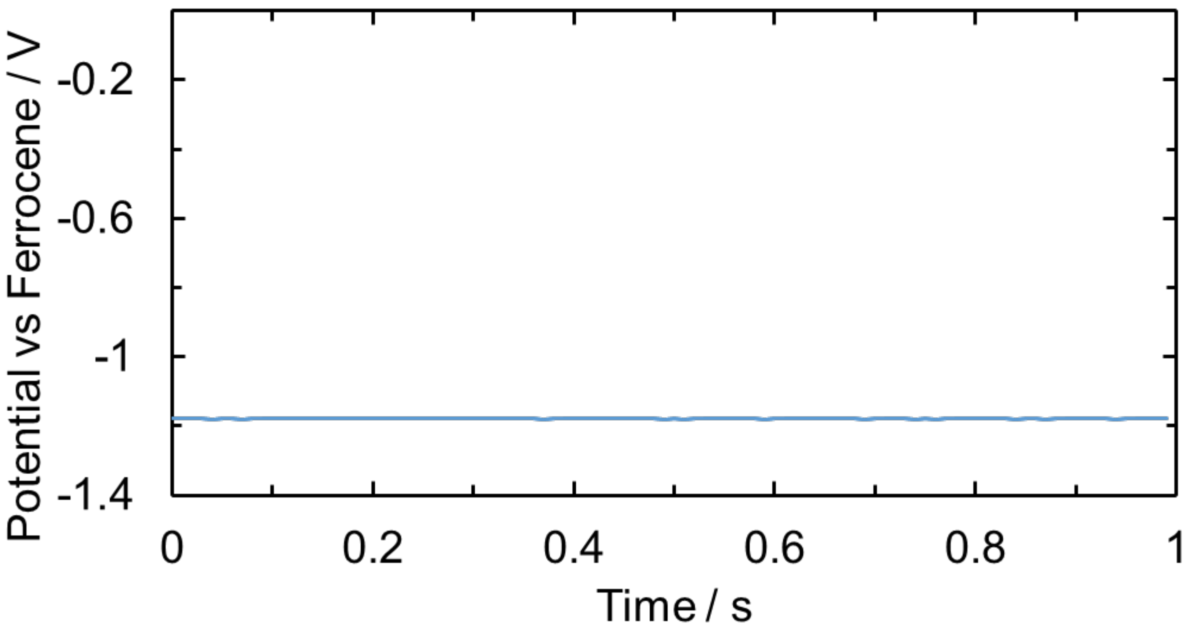

Figure S27. Open circuit potential of **U(IV)ExPh** determined at a concentration of 1 mM in THF containing 100 mM $TBAPF_6$. A quiet time of two seconds was followed by a one second measurement of the potential for the system. Potential measurements were referenced to a Ag wire and are reported with respect to ferrocene.

A full potential window CV was recorded for the synthesized U(IV) complex (**U(IV)ExPh**) resulting in the observation of two irreversible redox events with E_{pa} and E_{pc} separated by 2.4 V and 3.5 V, respectively. The initial oxidative scan shows a small pre-wave followed by a larger oxidation peak. The pre-wave was likely due to a mixed species in solution, *i.e.* monomer versus dimer made possible by the coordination of THF. The addition of ferrocene resulted in an asymmetric broadening of the larger oxidative peak and the disappearance of the pre-wave. A lack of ferrocene reduction was also noted. This result led to the suggestion that a stable U(V) complex could be accessed through oxidation with a ferrocenium salt. See main text for a discussion. A U(IV)/U(III) redox couple was not observed, providing evidence for the lack of a stable U(III) complex.

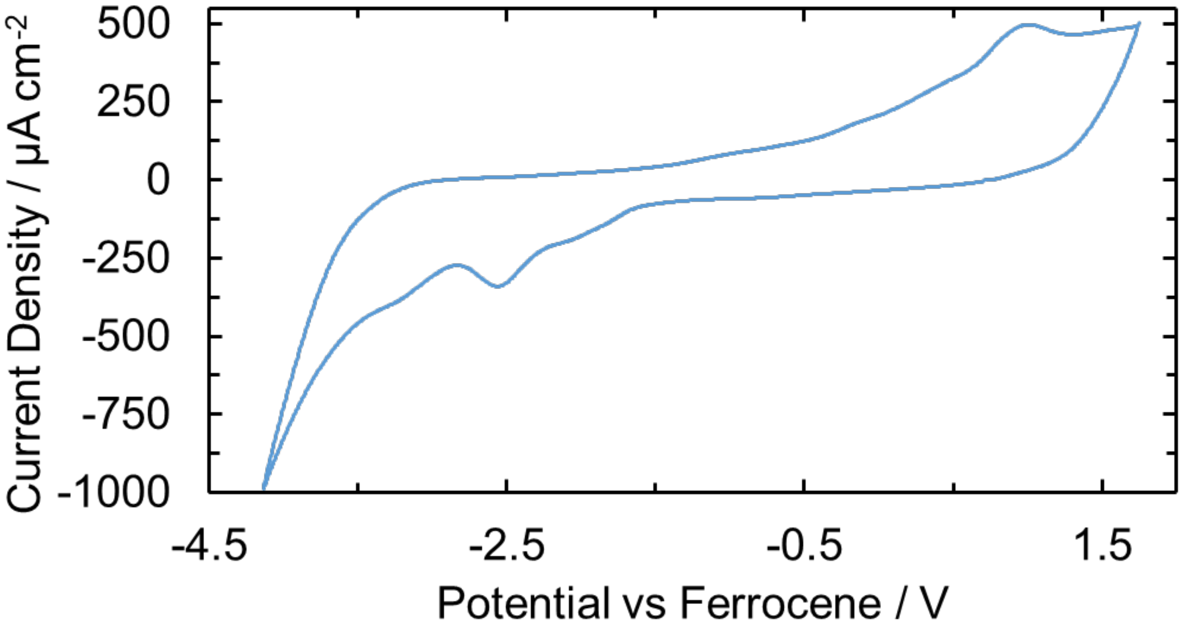

Figure S28. Cyclic voltammogram of **U(IV)ExPh** at a concentration of 1 mM in THF containing 100 mM $TBAPF_6$ at a scan rate of 100 mV/s. An irreversible pre-wave and a larger redox peak were seen ~ 2.4 V and 3.5 V apart, respectively, vs. ferrocene.

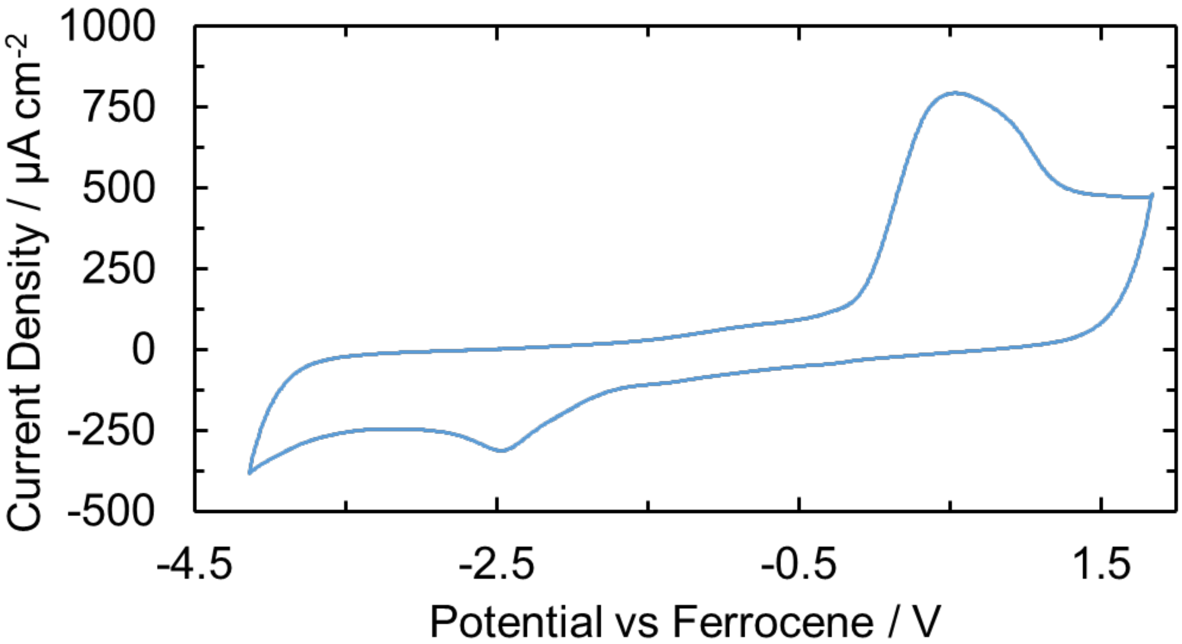

Figure S29. Cyclic voltammogram of **U(IV)ExPh** at a concentration of 1 mM in THF containing 100 mM $TBAPF_6$ and 5 mM ferrocene as an internal reference at a scan rate of 100 mV/s. The irreversible pre-wave was no longer observed, and the oxidative peak was broadened. A lack of ferrocene reduction was noted. See the main text for a discussion.

The above inferences were supported by DPV measurements carried out at a pulse height of 80 mV, a potential increment of 20 mV, a pulse width of 0.2 s, and a pulse period of 0.2 s. The differential pulse voltammogram swept anodically revealed a single oxidation peak. Following the peak, a non-zero steady

state current was observed. This latter observation is rationalized in terms of the ferrocene molecules behaving as an electron transfer mediator to the U(IV) complex as discussed in the main text.

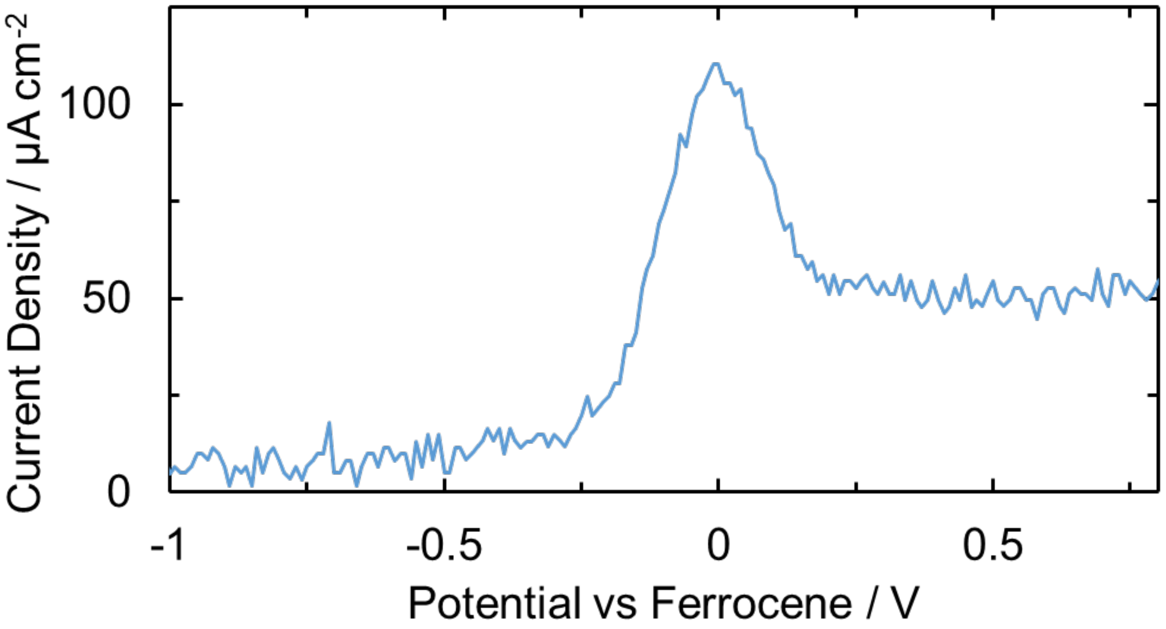

Figure S30. Differential pulse voltammogram of **U(IV)ExPh** at 1 mM concentration in THF containing 100 mM $TBAPF_6$ after the addition of 5 mM ferrocene as a potential reference. A single oxidation peak attributed to the ferrocene was observed. A non-zero steady state was seen after the initial peak, a finding ascribed to an electron transfer process involving the U(IV) species.

The initial inferences drawn from the electrochemical study using ferrocene as an internal standard yielded a promising route toward the chemical oxidation of **U(IV)ExPh**. However, the possibility of an unknown process occurring remained, given the irregular behavior of ferrocene. A non-overlapping electrochemical reference was desired to ensure this was not the case. Further cyclic voltammograms were recorded for the U(IV) complex using decamethylferrocene as an internal standard. These cyclic voltammetry measurements yielded the same pre-wave and peak seen for the U(IV) complex as the less oxidizing decamethylferrocenium peak was far enough removed from the analyte redox potentials. A return wave was noted for the decamethylferrocenium reduction upon the reverse scan.

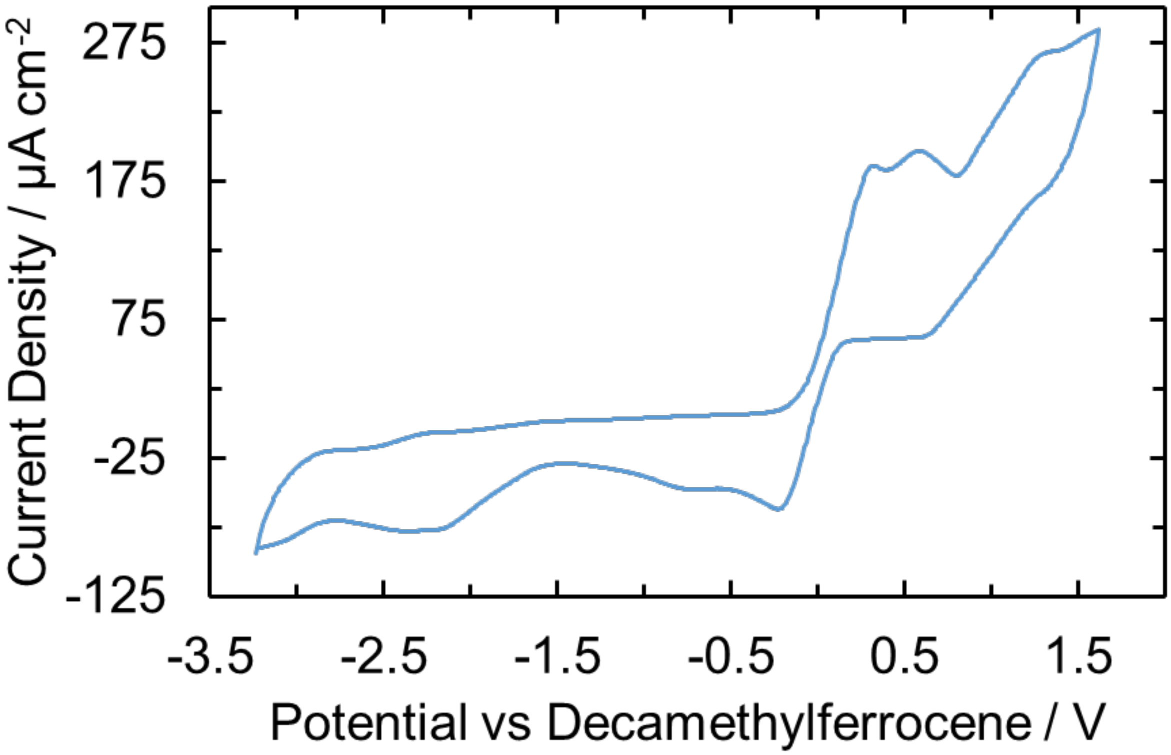

Figure S31. Cyclic voltammogram of **U(IV)ExPh** at a concentration of 1 mM in THF containing 100 mM $TBAPF_6$ and 2 mM decamethylferrocene as an internal reference at a scan rate of 100 mV/s. The irreversible redox peaks of the complex were observed (E_{pa} = 1.33 V, E_{pc} = -2.26 V) while the decamethylferrocene forward and reverse waves remained present.

To elucidate the relationship between the irreversible redox peaks seen for **U(IV)ExPh**, integration was performed on the current versus time plot of both the oxidation and reduction peaks for the CV scans at 100 mV/s. The total calculated charges for the oxidation and reduction peaks were 40.2 μC and 38.9 μC, respectively. The ratio of charges of oxidation to reduction was 0.97. The near-unity ratio of the calculated charges supported the relation between the oxidation and reduction peaks. Further, several cycles were scanned for only the oxidation wave at 100 mV/s (**Figure S32a**). Immediately after, the reduction wave was cycled at 100 mV/s (**Figure S32b**).

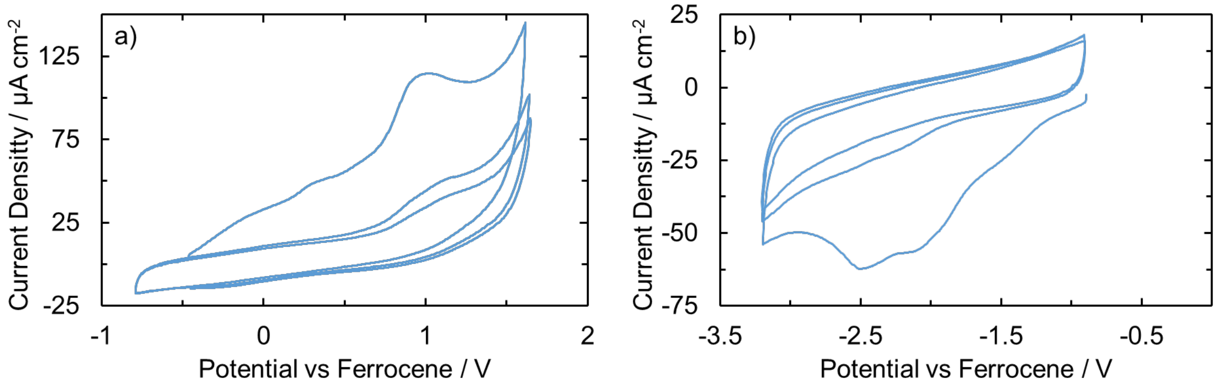

Figure S32. Cyclic voltammograms of **U(IV)ExPh** at a concentration of 1 mM in THF containing 100 mM $TBAPF_6$ at a scan rate of 100 mV/s. Cycling was performed independently for the a) oxidation and b) reduction regions of the full CV.

Integration of the first scan of the reduction peaks after cycling the oxidation region revealed a calculated charge of 60.3 μC. This charge was larger than the reduction wave of the full CV cycle by a factor of nearly 1.5. After two cycles of the reductive region, reductive peaks were no longer observed. This further supported the relation between the oxidation and reduction peaks observed in the CV. While cycling the oxidation region at 100 mV/s increased the charge calculated for the reduction peaks, the oxidation peaks also diminished upon each successive cycle until no distinction between analyte and background current was observed at cycle three. Cycling of the oxidative region was again performed at a scan rate of 25 mV/s to determine if the diminishing currents upon successive cycling was due to slow diffusion (**Figure S33**).

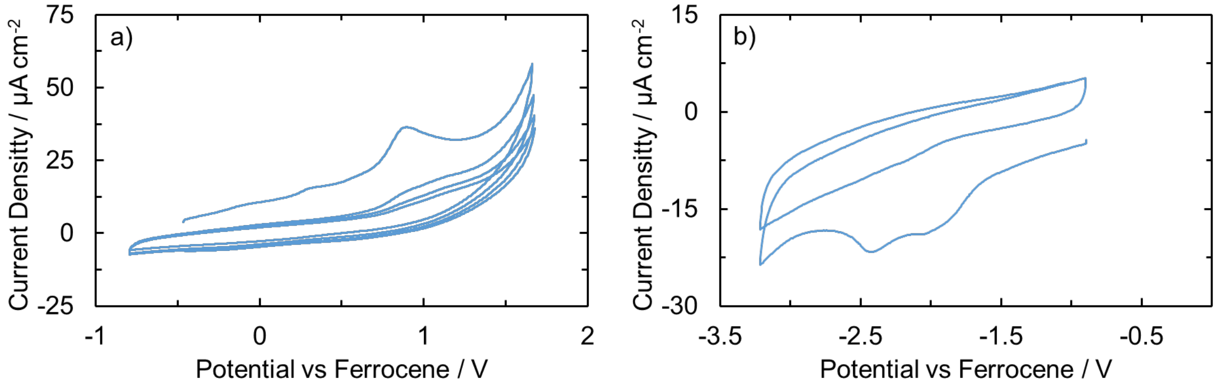

Figure S33. Cyclic voltammograms of **U(IV)ExPh** at a concentration of 1 mM in THF containing 100 mM $TBAPF_6$ at a scan rate of 25 mV/s. Cycling was performed independently for the a) oxidation and b) reduction regions of the full CV.

The same loss in oxidation peak was observed with no distinction between the U(IV) analyte and background by the third cycle of oxidation. Thus, we conclude that oxidation occurs via surface adsorption of **U(IV)ExPh** which fouls the electrochemical surface area. After two consecutive cycles over the oxidative region, active sites on the electrode surface are sufficiently blocked such that no further oxidation occurs. However, upon cycling to the reductive region, the electrochemical surface area is restored. This surface adsorptive process leads to slow electron transfer kinetics giving rise to the relatively large electrochemical irreversibility.

Spectro-Electrochemical Spectroscopy

Noting the possibility of a stable **U(V)ExPh** complex, a spectro-electrochemical study was performed to obtain spectroscopic data on the oxidized species. A 1.5 mL, 1 mM solution of **U(IV)ExPh** in THF containing 100 mM $TBAPF_6$ was used for this study. A gold honeycomb working electrode was inserted into a cuvette with a silver wire pseudo-reference electrode. A potential step from -1.0 V to 1.5 V was applied with initial and post-pulse UV-vis spectra being recorded. Upon re-equilibration, several 0.1 mL injections of atmospheric air were made to the sample followed by a final 0.5 mL injection of air. The broadened peak spanning the visible region from 450 to 650 nm complemented the solution- and solid-state UV-vis spectra recorded for **U(V)ExPh**.

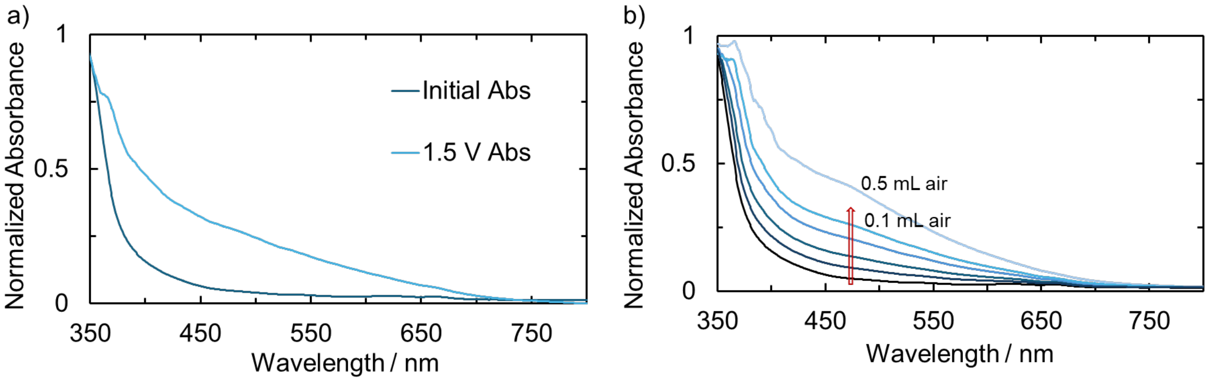

Figure S34. Normalized spectro-electrochemical UV-vis spectra of **U(IV)ExPh** measured in THF. a) Initial absorbance of **U(IV)ExPh** overlaid with the absorbance at a potential of 1.5 V vs. Ag/Ag^+ after 0.5 s. b) Absorbance spectra upon sequential injections of air into the cuvette.

Crystal Tables and Data

Complexes: CCDC Numbers

UExPh_DMAP: 2445901

UO2ExPh: 2445902

U(IV)ExPh:

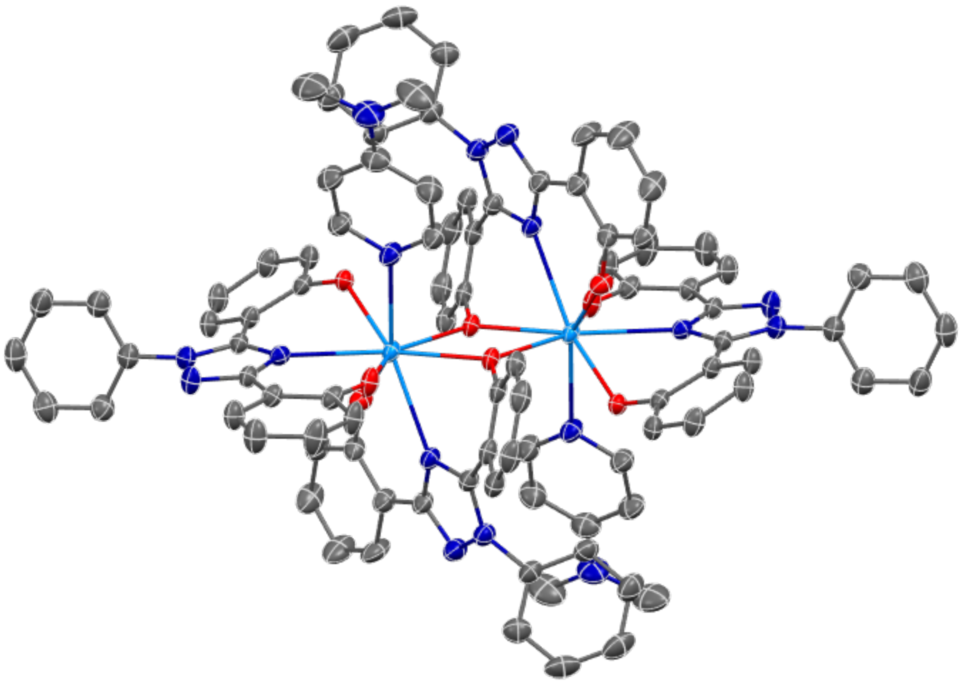

Figure S35. Crystal structure of **U(IV)ExPh** showing the 2:1 dimer with one bridging (L1) and one non-bridging (L2) ExPh ligand per metal center. Ellipsoids are drawn at the 50% probability with hydrogen and solvent molecules omitted for clarity.

Table S2. Crystal data and structure refinement for U(IV)ExPh

**Identification code**	uexph_dmap_a
**Empirical formula**	$C_{57} H_{56} N_8 O_{6.5} U$
**Formula weight**	1195.12
**Temperature/K**	101.0
**Crystal system**	triclinic
**Space group**	P-1
**a/Å**	11.9637(6)
**b/Å**	13.5417(7)
**c/Å**	15.7213(8)
**α/°**	67.8940(10)
**β/°**	88.4530(10)
**γ/°**	88.5700(10)
**Volume/Å^3**	2358.6(2)
**Z**	2
**ρ_{calc}g/cm^3**	1.683
**μ/mm^{-1}**	3.51
**F(000)**	1196.0
**Crystal size/mm^3**	0.10 × 0.15 × 0.15
**Radiation**	MoKα (λ = 0.71073)
**2Θ range for data collection/°**	4.44 to 55.84
**Index ranges**	$-14 \le h \le 14$, $-16 \le k \le 16$, $-19 \le l \le 19$
**Reflections collected**	36508
**Independent reflections**	9602 [R_{int} = 0.0358, R_{sigma} = 0.0357]

**Data/restraints/parameters**	9602/0/ 588
**Goodness-of-fit on F^2**	1.0810
**Final R indexes [I>=2σ (I)]**	R_1 = 0.0297, wR_2 = 0.0687
**Final R indexes [all data]**	R_1 = 0.0379, wR_2 = 0.0726
**Largest diff. peak/hole / e Å^{-3}**	2.5600/-1.2000

Table S3. Bond Lengths for **U(IV)ExPh**

Atom	Atom	Length/Å		Atom	Atom	Length/Å
U1	O2	2.487(2)		C47	H47C	0.981
U1	O1	2.164(3)		U1	O2	2.487(2)
U1	O3	2.176(3)		U1	O1	2.164(3)
U1	O4	2.276(2)		U1	O3	2.176(3)
U1	N1	2.604(3)		U1	O4	2.276(2)
U1	N7	2.612(4)		U1	N1	2.604(3)
U1	N4	2.576(3)		U1	N7	2.612(4)
U1	O2	2.403(3)		U1	N4	2.576(3)
O2	C10	1.350(5)		O2	C10	1.350(5)
O2	U1	2.403(3)		O1	C3	1.327(5)
O1	C3	1.327(5)		O3	C23	1.319(6)
O3	C23	1.319(6)		O4	C34	1.324(4)
O4	C34	1.324(4)		N1	C1	1.374(6)
N1	C1	1.374(6)		N1	C8	1.338(5)
N1	C8	1.338(5)		N2	N3	1.370(6)
N2	N3	1.370(6)		N2	C1	1.328(5)
N2	C1	1.328(5)		N3	C8	1.347(5)
N3	C8	1.347(5)		N3	C15	1.439(5)
N3	C15	1.439(5)		N7	C45	1.347(6)
N7	C45	1.347(6)		N7	C41	1.356(5)
N7	C41	1.356(5)		N4	C21	1.359(6)
N4	C21	1.359(6)		N4	C28	1.344(4)
N4	C28	1.344(4)		C1	C2	1.472(6)
C1	C2	1.472(6)		C2	C3	1.405(5)
C2	C3	1.405(5)		C2	C52	1.398(7)
C2	C52	1.398(7)		C8	C9	1.465(6)
C8	C9	1.465(6)		C3	C4	1.401(6)
C3	C4	1.401(6)		C9	C10	1.411(5)
C9	C10	1.411(5)		C9	C14	1.401(6)
C9	C14	1.401(6)		C10	C11	1.400(7)
C10	C11	1.400(7)		C15	C16	1.372(7)
C15	C16	1.372(7)		C15	C20	1.391(6)
C15	C20	1.391(6)		C45	H45	0.949
C45	H45	0.949		C45	C44	1.374(7)
C45	C44	1.374(7)		N5	N6	1.404(5)
N5	N6	1.404(5)		N5	C21	1.333(5)
N5	C21	1.333(5)		C41	H41	0.95

C41	H41	0.95		C41	C42	1.360(7)
C41	C42	1.360(7)		C23	C22	1.395(5)
C23	C22	1.395(5)		C23	C24	1.415(5)
C23	C24	1.415(5)		C11	H11	0.95
C11	H11	0.95		C11	C12	1.387(6)
C11	C12	1.387(6)		C22	C21	1.475(5)
C22	C21	1.475(5)		C22	C27	1.400(7)
C22	C27	1.400(7)		C16	H16	0.95
C16	H16	0.95		C16	C17	1.390(5)
C16	C17	1.390(5)		C29	C34	1.424(5)
C29	C34	1.424(5)		C29	C30	1.404(6)
C29	C30	1.404(6)		C29	C28	1.473(6)
C29	C28	1.473(6)		C44	H44	0.95
C44	H44	0.95		C44	C43	1.407(6)
C44	C43	1.407(6)		C4	H4	0.95
C4	H4	0.95		C4	C5	1.378(7)
C4	C5	1.378(7)		C12	H12	0.951
C12	H12	0.951		C12	C13	1.376(6)
C12	C13	1.376(6)		N8	C43	1.356(7)
N8	C43	1.356(7)		N8	C46	1.465(7)
N8	C46	1.465(7)		N8	C47	1.449(8)
N8	C47	1.449(8)		N6	C28	1.342(5)
N6	C28	1.342(5)		N6	C35	1.423(5)
N6	C35	1.423(5)		C52	H52	0.95
C52	H52	0.95		C52	C48	1.373(8)
C52	C48	1.373(8)		C43	C42	1.409(7)
C43	C42	1.409(7)		C33	H33	0.95
C33	H33	0.95		C33	C32	1.372(6)
C33	C32	1.372(6)		C33	C34	1.409(6)
C33	C34	1.409(6)		C5	C48	1.386(6)
C5	C48	1.386(6)		C5	H5	0.91(5)
C5	H5	0.91(5)		C42	H42	0.95
C42	H42	0.95		C32	H32	0.95
C32	H32	0.95		C32	C31	1.400(7)
C32	C31	1.400(7)		C14	H14	0.95
C14	H14	0.95		C14	C13	1.386(7)
C14	C13	1.386(7)		C30	H30	0.95
C30	H30	0.95		C30	C31	1.375(6)
C30	C31	1.375(6)		C31	H31	0.95
C31	H31	0.95		C13	H13	0.95
C13	H13	0.95		C24	H24	0.95
C24	H24	0.95		C24	C25	1.372(8)
C24	C25	1.372(8)		C27	H27	0.95
C27	H27	0.95		C27	C26	1.380(6)
C27	C26	1.380(6)		C20	C19	1.389(6)

C20	C19	1.389(6)		C20	H20	0.94(5)
C20	H20	0.94(5)		C39	H39	0.949
C39	H39	0.949		C39	C40	1.385(6)
C39	C40	1.385(6)		C39	C38	1.375(6)
C39	C38	1.375(6)		C48	H48	0.95
C48	H48	0.95		C17	H17	0.949
C17	H17	0.949		C17	C18	1.383(7)
C17	C18	1.383(7)		C40	H40	0.95
C40	H40	0.95		C40	C35	1.385(7)
C40	C35	1.385(7)		C36	H36	0.949
C36	H36	0.949		C36	C35	1.375(6)
C36	C35	1.375(6)		C36	C37	1.388(6)
C36	C37	1.388(6)		C38	H38	0.95
C38	H38	0.95		C38	C37	1.387(7)
C38	C37	1.387(7)		C25	H25	0.951
C25	H25	0.951		C25	C26	1.389(7)
C25	C26	1.389(7)		C26	H26	0.95
C26	H26	0.95		C19	C18	1.380(8)
C19	C18	1.380(8)		C19	H19	0.95(6)
C19	H19	0.95(6)		C18	H18	0.951
C18	H18	0.951		C37	H37	0.95
C37	H37	0.95		C46	H46A	0.979
C46	H46A	0.979		C46	H46B	0.981
C46	H46B	0.981		C46	H46C	0.98
C46	H46C	0.98		C47	H47A	0.98
C47	H47A	0.98		C47	H47B	0.98
C47	H47B	0.98		C47	H47C	0.981

Table S4. Bond Angles for **U(IV)ExPh**

**Atom**	**Atom**	**Atom**	**Angle/°**		**Atom**	**Atom**	**Atom**	**Angle/°**
O2	U1	O1	125.04(9)		O2	U1	O2	64.14(9)
O2	U1	O3	79.29(9)		O2	U1	O1	83.91(9)
O2	U1	O4	133.46(9)		O2	U1	O3	143.43(9)
O2	U1	N1	67.8(1)		O2	U1	O4	82.37(9)
O2	U1	N7	81.7(1)		O2	U1	N1	88.3(1)
O2	U1	N4	147.35(9)		O2	U1	N7	96.6(1)
O2	U1	O2	64.14(9)		O2	U1	N4	148.01(9)
O1	U1	O3	119.5(1)		O2	U1	O1	125.04(9)
O1	U1	O4	78.7(1)		O2	U1	O3	79.29(9)
O1	U1	N1	67.8(1)		O2	U1	O4	133.46(9)
O1	U1	N7	148.9(1)		O2	U1	N1	67.8(1)
O1	U1	N4	77.4(1)		O2	U1	N7	81.7(1)
O1	U1	O2	83.91(9)		O2	U1	N4	147.35(9)
O3	U1	O4	127.1(1)		O1	U1	O3	119.5(1)
O3	U1	N1	77.1(1)		O1	U1	O4	78.7(1)

O3	U1	N7	77.5(1)		O1	U1	N1	67.8(1)
O3	U1	N4	68.4(1)		O1	U1	N7	148.9(1)
O3	U1	O2	143.43(9)		O1	U1	N4	77.4(1)
O4	U1	N1	146.0(1)		O3	U1	O4	127.1(1)
O4	U1	N7	70.6(1)		O3	U1	N1	77.1(1)
O4	U1	N4	68.8(1)		O3	U1	N7	77.5(1)
O4	U1	O2	82.37(9)		O3	U1	N4	68.4(1)
N1	U1	N7	143.2(1)		O4	U1	N1	146.0(1)
N1	U1	N4	107.8(1)		O4	U1	N7	70.6(1)
N1	U1	O2	88.3(1)		O4	U1	N4	68.8(1)
N7	U1	N4	86.8(1)		N1	U1	N7	143.2(1)
N7	U1	O2	96.6(1)		N1	U1	N4	107.8(1)
N4	U1	O2	148.01(9)		N7	U1	N4	86.8(1)
U1	O2	C10	118.9(2)		U1	O2	U1	115.9(1)
U1	O2	U1	115.9(1)		U1	O2	C10	122.7(2)
C10	O2	U1	122.7(2)		U1	O2	C10	118.9(2)
U1	O1	C3	153.9(3)		U1	O1	C3	153.9(3)
U1	O3	C23	152.5(3)		U1	O3	C23	152.5(3)
U1	O4	C34	130.1(2)		U1	O4	C34	130.1(2)
U1	N1	C1	130.5(3)		U1	N1	C1	130.5(3)
U1	N1	C8	125.0(3)		U1	N1	C8	125.0(3)
C1	N1	C8	104.2(3)		C1	N1	C8	104.2(3)
N3	N2	C1	103.1(3)		N3	N2	C1	103.1(3)
N2	N3	C8	110.3(3)		N2	N3	C8	110.3(3)
N2	N3	C15	118.8(3)		N2	N3	C15	118.8(3)
C8	N3	C15	130.1(4)		C8	N3	C15	130.1(4)
U1	N7	C45	125.3(3)		U1	N7	C45	125.3(3)
U1	N7	C41	118.9(3)		U1	N7	C41	118.9(3)
C45	N7	C41	114.7(4)		C45	N7	C41	114.7(4)
U1	N4	C21	130.4(3)		U1	N4	C21	130.4(3)
U1	N4	C28	125.1(2)		U1	N4	C28	125.1(2)
C21	N4	C28	103.9(3)		C21	N4	C28	103.9(3)
N1	C1	N2	113.4(4)		N1	C1	N2	113.4(4)
N1	C1	C2	125.6(4)		N1	C1	C2	125.6(4)
N2	C1	C2	121.0(4)		N2	C1	C2	121.0(4)
C1	C2	C3	120.9(4)		C1	C2	C3	120.9(4)
C1	C2	C52	120.2(4)		C1	C2	C52	120.2(4)
C3	C2	C52	118.8(4)		C3	C2	C52	118.8(4)
N1	C8	N3	109.0(4)		N1	C8	N3	109.0(4)
N1	C8	C9	125.8(4)		N1	C8	C9	125.8(4)
N3	C8	C9	125.1(4)		N3	C8	C9	125.1(4)
O1	C3	C2	120.9(4)		O1	C3	C2	120.9(4)
O1	C3	C4	119.6(4)		O1	C3	C4	119.6(4)
C2	C3	C4	119.5(4)		C2	C3	C4	119.5(4)
C8	C9	C10	119.6(4)		C8	C9	C10	119.6(4)

C8	C9	C14	120.9(4)		C8	C9	C14	120.9(4)
C10	C9	C14	119.5(4)		C10	C9	C14	119.5(4)
O2	C10	C9	120.3(4)		O2	C10	C9	120.3(4)
O2	C10	C11	121.0(4)		O2	C10	C11	121.0(4)
C9	C10	C11	118.7(4)		C9	C10	C11	118.7(4)
N3	C15	C16	119.8(4)		N3	C15	C16	119.8(4)
N3	C15	C20	118.1(4)		N3	C15	C20	118.1(4)
C16	C15	C20	122.1(4)		C16	C15	C20	122.1(4)
N7	C45	H45	117.6		N7	C45	H45	117.6
N7	C45	C44	124.8(5)		N7	C45	C44	124.8(5)
H45	C45	C44	117.6		H45	C45	C44	117.6
N6	N5	C21	101.6(3)		N6	N5	C21	101.6(3)
N7	C41	H41	117.4		N7	C41	H41	117.4
N7	C41	C42	125.0(5)		N7	C41	C42	125.0(5)
H41	C41	C42	117.6		H41	C41	C42	117.6
O3	C23	C22	121.4(4)		O3	C23	C22	121.4(4)
O3	C23	C24	119.8(4)		O3	C23	C24	119.8(4)
C22	C23	C24	118.8(4)		C22	C23	C24	118.8(4)
C10	C11	H11	119.7		C10	C11	H11	119.7
C10	C11	C12	120.7(4)		C10	C11	C12	120.7(4)
H11	C11	C12	119.7		H11	C11	C12	119.7
C23	C22	C21	120.8(4)		C23	C22	C21	120.8(4)
C23	C22	C27	119.4(4)		C23	C22	C27	119.4(4)
C21	C22	C27	119.7(4)		C21	C22	C27	119.7(4)
C15	C16	H16	120.4		C15	C16	H16	120.4
C15	C16	C17	119.2(4)		C15	C16	C17	119.2(4)
H16	C16	C17	120.4		H16	C16	C17	120.4
C34	C29	C30	119.3(4)		C34	C29	C30	119.3(4)
C34	C29	C28	119.8(3)		C34	C29	C28	119.8(3)
C30	C29	C28	120.9(4)		C30	C29	C28	120.9(4)
C45	C44	H44	120.2		C45	C44	H44	120.2
C45	C44	C43	119.6(5)		C45	C44	C43	119.6(5)
H44	C44	C43	120.2		H44	C44	C43	120.2
C3	C4	H4	119.8		C3	C4	H4	119.8
C3	C4	C5	120.4(4)		C3	C4	C5	120.4(4)
H4	C4	C5	119.9		H4	C4	C5	119.9
C11	C12	H12	119.7		C11	C12	H12	119.7
C11	C12	C13	120.6(4)		C11	C12	C13	120.6(4)
H12	C12	C13	119.6		H12	C12	C13	119.6
C43	N8	C46	119.8(5)		C43	N8	C46	119.8(5)
C43	N8	C47	121.2(5)		C43	N8	C47	121.2(5)
C46	N8	C47	118.8(5)		C46	N8	C47	118.8(5)
N5	N6	C28	109.9(3)		N5	N6	C28	109.9(3)
N5	N6	C35	115.9(3)		N5	N6	C35	115.9(3)
C28	N6	C35	134.2(4)		C28	N6	C35	134.2(4)

C2	C52	H52	119.6		C2	C52	H52	119.6
C2	C52	C48	120.9(5)		C2	C52	C48	120.9(5)
H52	C52	C48	119.5		H52	C52	C48	119.5
C44	C43	N8	122.3(5)		C44	C43	N8	122.3(5)
C44	C43	C42	115.9(4)		C44	C43	C42	115.9(4)
N8	C43	C42	121.8(5)		N8	C43	C42	121.8(5)
H33	C33	C32	119.1		H33	C33	C32	119.1
H33	C33	C34	119		H33	C33	C34	119
C32	C33	C34	121.9(4)		C32	C33	C34	121.9(4)
C4	C5	C48	120.0(5)		C4	C5	C48	120.0(5)
C4	C5	H5	116(3)		C4	C5	H5	116(3)
C48	C5	H5	124(4)		C48	C5	H5	124(4)
C41	C42	C43	119.8(5)		C41	C42	C43	119.8(5)
C41	C42	H42	120		C41	C42	H42	120
C43	C42	H42	120.1		C43	C42	H42	120.1
C33	C32	H32	119.8		C33	C32	H32	119.8
C33	C32	C31	120.3(4)		C33	C32	C31	120.3(4)
H32	C32	C31	119.8		H32	C32	C31	119.8
O4	C34	C29	122.6(3)		O4	C34	C29	122.6(3)
O4	C34	C33	119.9(3)		O4	C34	C33	119.9(3)
C29	C34	C33	117.5(3)		C29	C34	C33	117.5(3)
C9	C14	H14	119.7		C9	C14	H14	119.7
C9	C14	C13	120.6(4)		C9	C14	C13	120.6(4)
H14	C14	C13	119.7		H14	C14	C13	119.7
C29	C30	H30	119.2		C29	C30	H30	119.2
C29	C30	C31	121.6(4)		C29	C30	C31	121.6(4)
H30	C30	C31	119.2		H30	C30	C31	119.2
N4	C21	N5	115.0(4)		N4	C21	N5	115.0(4)
N4	C21	C22	126.0(4)		N4	C21	C22	126.0(4)
N5	C21	C22	119.0(4)		N5	C21	C22	119.0(4)
C32	C31	C30	119.2(4)		C32	C31	C30	119.2(4)
C32	C31	H31	120.4		C32	C31	H31	120.4
C30	C31	H31	120.4		C30	C31	H31	120.4
C12	C13	C14	119.9(4)		C12	C13	C14	119.9(4)
C12	C13	H13	120.1		C12	C13	H13	120.1
C14	C13	H13	120		C14	C13	H13	120
N4	C28	C29	125.8(3)		N4	C28	C29	125.8(3)
N4	C28	N6	109.6(3)		N4	C28	N6	109.6(3)
C29	C28	N6	124.6(3)		C29	C28	N6	124.6(3)
C23	C24	H24	119.7		C23	C24	H24	119.7
C23	C24	C25	120.5(4)		C23	C24	C25	120.5(4)
H24	C24	C25	119.8		H24	C24	C25	119.8
C22	C27	H27	119.4		C22	C27	H27	119.4
C22	C27	C26	121.2(4)		C22	C27	C26	121.2(4)
H27	C27	C26	119.4		H27	C27	C26	119.4

C15	C20	C19	117.8(5)		C15	C20	C19	117.8(5)
C15	C20	H20	123(3)		C15	C20	H20	123(3)
C19	C20	H20	119(3)		C19	C20	H20	119(3)
H39	C39	C40	120.2		H39	C39	C40	120.2
H39	C39	C38	120.2		H39	C39	C38	120.2
C40	C39	C38	119.7(5)		C40	C39	C38	119.7(5)
C52	C48	C5	120.3(5)		C52	C48	C5	120.3(5)
C52	C48	H48	119.8		C52	C48	H48	119.8
C5	C48	H48	119.8		C5	C48	H48	119.8
C16	C17	H17	120		C16	C17	H17	120
C16	C17	C18	119.8(5)		C16	C17	C18	119.8(5)
H17	C17	C18	120.1		H17	C17	C18	120.1
C39	C40	H40	120.3		C39	C40	H40	120.3
C39	C40	C35	119.4(5)		C39	C40	C35	119.4(5)
H40	C40	C35	120.3		H40	C40	C35	120.3
H36	C36	C35	120.5		H36	C36	C35	120.5
H36	C36	C37	120.5		H36	C36	C37	120.5
C35	C36	C37	119.0(4)		C35	C36	C37	119.0(4)
N6	C35	C40	119.0(4)		N6	C35	C40	119.0(4)
N6	C35	C36	119.6(4)		N6	C35	C36	119.6(4)
C40	C35	C36	121.4(4)		C40	C35	C36	121.4(4)
C39	C38	H38	119.6		C39	C38	H38	119.6
C39	C38	C37	120.7(5)		C39	C38	C37	120.7(5)
H38	C38	C37	119.6		H38	C38	C37	119.6
C24	C25	H25	119.6		C24	C25	H25	119.6
C24	C25	C26	120.8(5)		C24	C25	C26	120.8(5)
H25	C25	C26	119.7		H25	C25	C26	119.7
C27	C26	C25	119.2(5)		C27	C26	C25	119.2(5)
C27	C26	H26	120.4		C27	C26	H26	120.4
C25	C26	H26	120.4		C25	C26	H26	120.4
C20	C19	C18	121.0(5)		C20	C19	C18	121.0(5)
C20	C19	H19	114(4)		C20	C19	H19	114(4)
C18	C19	H19	125(4)		C18	C19	H19	125(4)
C17	C18	C19	120.1(5)		C17	C18	C19	120.1(5)
C17	C18	H18	120		C17	C18	H18	120
C19	C18	H18	119.9		C19	C18	H18	119.9
C36	C37	C38	119.8(5)		C36	C37	C38	119.8(5)
C36	C37	H37	120.1		C36	C37	H37	120.1
C38	C37	H37	120.1		C38	C37	H37	120.1
N8	C46	H46A	109.5		N8	C46	H46A	109.5
N8	C46	H46B	109.4		N8	C46	H46B	109.4
N8	C46	H46C	109.5		N8	C46	H46C	109.5
H46A	C46	H46B	109.5		H46A	C46	H46B	109.5
H46A	C46	H46C	109.5		H46A	C46	H46C	109.5
H46B	C46	H46C	109.4		H46B	C46	H46C	109.4

N8	C47	H47A	109.5		N8	C47	H47A	109.5
N8	C47	H47B	109.5		N8	C47	H47B	109.5
N8	C47	H47C	109.4		N8	C47	H47C	109.4
H47A	C47	H47B	109.5		H47A	C47	H47B	109.5
H47A	C47	H47C	109.4		H47A	C47	H47C	109.4
H47B	C47	H47C	109.4		H47B	C47	H47C	109.4

UO2ExPh:

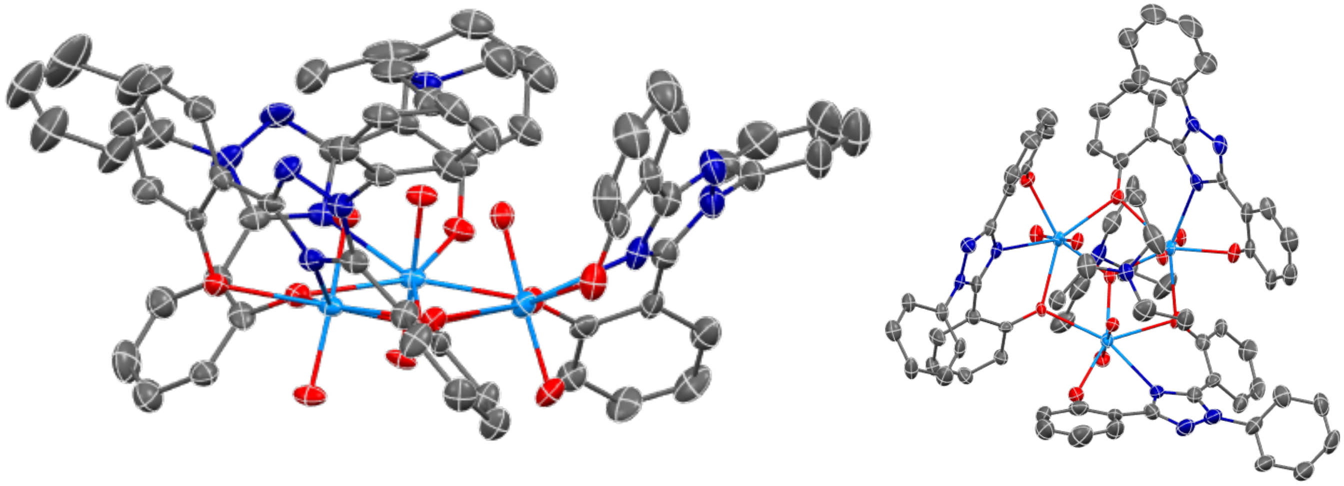

Figure S36. Crystal structure of **UO_2ExPh** showing the 1:1 trimeric bowl shape (side view left; top-down view right). Ellipsoids are drawn at the 50% probability level with hydrogen and solvent molecules omitted for clarity.

Table S5. Crystal data and structure refinement for UO_2ExPh

**Identification code**	data_peetwooneoncee_a
**Empirical formula**	$C_{72}H_{73}N_{11}O_{13}U_3$
**Formula weight**	2014.50
**Temperature/K**	100.00
**Crystal system**	monoclinic
**Space group**	P 1 21/c 1
**a/Å**	16.4420(6)
**b/Å**	17.7005(6)
**c/Å**	26.7887(9)
**α/°**	90
**β/°**	91.5440(10)
**γ/°**	90
**Volume/Å^3**	7793.5(5)
**Z**	4
**ρ_{calc}g/cm^3**	1.717
**μ/mm^{-1}**	6.28
**F(000)**	3848.0
**Crystal size/mm^3**	0.20 × 0.20 × 0.25
**Radiation**	MoKα (λ = 0.71073)
**2Θ range for data collection/°**	5.50 to 56.80
**Index ranges**	$-22 \leq h \leq 20$, $-23 \leq k \leq 23$, $-34 \leq l \leq 35$
**Reflections collected**	64779
**Independent reflections**	19664 [R_{int} = 0.0894, R_{sigma} = 0.1026]
**Data/restraints/parameters**	19664/0/898
**Goodness-of-fit on F^2**	1.023
**Final R indexes [I>=2σ (I)]**	R_1 = 0.0525, wR_2 = 0.1027
**Final R indexes [all data]**	R_1 = 0.0987, wR_2 = 0.1149
**Largest diff. peak/hole / e Å^{-3}**	2.91/-2.65

**Table S6**. Bond Lengths for **UO_2ExPh**						
Atom	Atom	Length		Atom	Atom	Length
U001	O1	2.224(5)		C01M	C01X	1.39(1)
U001	O006	1.778(5)		C01N	C028	1.41(1)
U001	O00A	2.458(5)		C01O	C01S	1.36(1)
U001	O00B	1.799(5)		C01P	H01P	0.95
U001	O00C	2.434(5)		C01Q	H01Q	0.95
U001	O00F	2.315(5)		C01S	H01S	0.949
U001	N3	2.559(5)		C01S	C020	1.36(1)
U002	O004	1.782(5)		C01T	H01T	0.95
U002	O1	2.263(5)		C01T	C02I	1.37(2)
U002	O2	2.434(5)		C01V	C02D	1.37(1)
U002	O008	1.804(5)		C01V	C02J	1.37(1)
U002	O00A	2.474(5)		C01W	C02H	1.38(1)
U002	O00E	2.227(5)		C01W	C02O	1.38(1)
U002	N4	2.542(6)		C01X	H01X	0.95
U003	O1	2.265(5)		C01X	C01Z	1.41(1)
U003	O2	2.469(5)		C01Y	H01Y	0.95
U003	O009	1.774(5)		C01Y	C029	1.37(1)
U003	O00C	2.457(5)		C01Z	H01Z	0.95
U003	O00D	1.783(5)		C020	H020	0.95
U003	O00G	2.270(5)		C021	C02E	1.39(1)
U003	N00M	2.535(6)		C022	H022	0.95
O2	C2	1.354(9)		C024	H024	0.95
O00A	C00R	1.333(9)		C024	C02A	1.37(1)
O00C	C1	1.345(9)		C028	H028	0.95
O00E	C018	1.331(9)		C028	C02G	1.36(1)
O00F	C01J	1.342(9)		C029	H029	0.95
O00G	C014	1.33(1)		C029	C02G	1.40(1)
N3	C011	1.35(1)		C02A	H02A	0.95
N3	C015	1.37(1)		C02B	H02B	0.95
N4	C00S	1.38(1)		C02B	C02D	1.38(1)
N4	C00Y	1.34(1)		C02B	C02M	1.36(2)
N00K	H00K	0.881		C02D	H02D	0.95
N00K	N00L	1.375(9)		C02E	H02E	0.95
N00K	C015	1.32(1)		C02E	C02N	1.37(1)
N00L	C011	1.361(9)		C02G	H02G	0.949
N00L	C01O	1.45(1)		C02H	H02H	0.95
N00M	C013	1.31(1)		C02H	C02Q	1.39(1)
N00M	C01L	1.37(1)		C02I	H02I	0.95
C2	C012	1.40(1)		C02I	C02N	1.37(2)
C2	C016	1.41(1)		C02J	H02J	0.95
N00P	N00X	1.37(1)		C02J	C02L	1.36(2)
N00P	C00Y	1.35(1)		C02L	H02L	0.95

N00P	C01W	1.43(1)		C02L	C02M	1.36(2)
C4	H4	0.95		C02M	H02M	0.95
C4	C00R	1.40(1)		C02N	H02N	0.95
C4	C01D	1.37(1)		C02O	H02O	0.95
C00R	C3	1.42(1)		C02O	C02P	1.40(2)
C00S	N00X	1.35(1)		C02P	H02P	0.95
C00S	C01B	1.45(1)		C02P	C02R	1.35(2)
C1	C00Z	1.40(1)		C02Q	H02Q	0.95
C1	C01R	1.40(1)		C02Q	C02R	1.33(2)
N00U	H00U	0.879		C02R	H02R	0.95
N00U	N2	1.36(1)		N00H	C01U	1.49(1)
N00U	C01L	1.32(1)		N00H	C023	1.46(1)
C3	C00Y	1.45(1)		N00H	C026	1.50(1)
C3	C019	1.40(1)		C01E	H01R	0.98
N2	C013	1.37(1)		C01E	H01U	0.98
N2	C01V	1.44(1)		C01E	H01V	0.98
N00X	H00X	0.88		C01E	C023	1.54(1)
C00Z	H00Z	0.95		C01U	H01W	0.99
C00Z	C01C	1.37(1)		C01U	H01	0.99
C011	C01R	1.45(1)		C01U	C027	1.59(1)
C012	H012	0.95		C023	H02X	0.991
C012	C01Z	1.37(1)		C023	H02Y	0.989
C013	C016	1.44(1)		C025	H02Z	0.98
C014	C01T	1.41(1)		C025	H	0.981
C014	C021	1.40(1)		C025	HA	0.979
C015	C01N	1.46(1)		C025	C026	1.52(1)
C016	C01M	1.41(1)		C026	H02	0.99
C018	C01B	1.41(1)		C026	HB	0.99
C018	C01Q	1.38(1)		C027	H1	0.981
C019	H019	0.95		C027	HC	0.98
C019	C01P	1.40(1)		C027	HD	0.98
C01A	H01A	0.949		N00N	H00N	1.001
C01A	C01O	1.40(1)		N00N	C010	1.49(1)
C01A	C022	1.37(1)		N00N	C017	1.50(1)
C01B	C024	1.41(1)		N00N	C02F	1.50(1)
C01C	H01C	0.95		C010	H01B	0.989
C01C	C01K	1.39(1)		C010	H01E	0.991
C01D	H01D	0.95		C010	C01G	1.53(1)
C01D	C01P	1.40(1)		C017	H01G	0.99
C01F	H01F	0.95		C017	H01J	0.989
C01F	C01Q	1.39(1)		C017	C02K	1.49(1)
C01F	C02A	1.33(1)		C01G	H01L	0.98
C01H	H01H	0.95		C01G	H01N	0.98
C01H	C01K	1.38(1)		C01G	H01O	0.979
C01H	C01R	1.40(1)		C02C	H02C	0.98

C01I	H01I	0.951		C02C	H02F	0.98
C01I	C020	1.38(1)		C02C	H02K	0.98
C01I	C022	1.40(1)		C02C	C02F	1.53(1)
C01J	C01N	1.39(1)		C02F	H02S	0.989
C01J	C01Y	1.42(1)		C02F	H02T	0.989
C01K	H01K	0.95		C02K	H02U	0.98
C01L	C021	1.45(1)		C02K	H02V	0.98
C01M	H01M	0.949		C02K	H02W	0.98

**Table S7**. Bond Angles for **UO_2ExPh**								
Atom	Atom	Atom	Angle		Atom	Atom	Atom	Angle
O1	U001	O006	88.8(2)		C016	C01M	H01M	119.7
O1	U001	O00A	69.8(2)		C016	C01M	C01X	120.6(8)
O1	U001	O00B	95.4(2)		H01M	C01M	C01X	119.7
O1	U001	O00C	69.9(2)		C015	C01N	C01J	122.0(7)
O1	U001	O00F	153.8(2)		C015	C01N	C028	119.3(7)
O1	U001	N3	135.6(2)		C01J	C01N	C028	118.7(7)
O006	U001	O00A	83.0(2)		N00L	C01O	C01A	118.8(7)
O006	U001	O00B	175.8(2)		N00L	C01O	C01S	120.3(7)
O006	U001	O00C	90.7(2)		C01A	C01O	C01S	120.6(7)
O006	U001	O00F	90.3(2)		C019	C01P	C01D	119.6(9)
O006	U001	N3	76.1(2)		C019	C01P	H01P	120
O00A	U001	O00B	98.9(2)		C01D	C01P	H01P	120
O00A	U001	O00C	139.3(2)		C018	C01Q	C01F	120.8(8)
O00A	U001	O00F	84.0(2)		C018	C01Q	H01Q	119.7
O00A	U001	N3	145.5(2)		C01F	C01Q	H01Q	119.5
O00B	U001	O00C	90.3(2)		C1	C01R	C011	120.0(7)
O00B	U001	O00F	86.1(2)		C1	C01R	C01H	118.7(7)
O00B	U001	N3	100.4(2)		C011	C01R	C01H	121.3(7)
O00C	U001	O00F	136.3(2)		C01O	C01S	H01S	119.7
O00C	U001	N3	68.9(2)		C01O	C01S	C020	120.6(8)
O00F	U001	N3	69.1(2)		H01S	C01S	C020	119.7
O004	U002	O1	88.6(2)		C014	C01T	H01T	120
O004	U002	O2	87.8(2)		C014	C01T	C02I	121(1)
O004	U002	O008	175.7(2)		H01T	C01T	C02I	120
O004	U002	O00A	92.3(2)		N2	C01V	C02D	119.8(8)
O004	U002	O00E	84.6(2)		N2	C01V	C02J	119.8(8)
O004	U002	N4	81.3(2)		C02D	C01V	C02J	120.4(9)
O1	U002	O2	69.3(2)		N00P	C01W	C02H	121.1(8)
O1	U002	O008	95.5(2)		N00P	C01W	C02O	119.2(8)
O1	U002	O00A	69.0(2)		C02H	C01W	C02O	119.5(9)
O1	U002	O00E	151.4(2)		C01M	C01X	H01X	119.9
O1	U002	N4	137.4(2)		C01M	C01X	C01Z	120.1(9)
O2	U002	O008	94.6(2)		H01X	C01X	C01Z	120
O2	U002	O00A	138.2(2)		C01J	C01Y	H01Y	119.2

O2	U002	O00E	82.7(2)		C01J	C01Y	C029	121.4(8)
O2	U002	N4	150.3(2)		H01Y	C01Y	C029	119.4
O008	U002	O00A	88.2(2)		C012	C01Z	C01X	119.1(9)
O008	U002	O00E	92.3(2)		C012	C01Z	H01Z	120.5
O008	U002	N4	94.9(2)		C01X	C01Z	H01Z	120
O00A	U002	O00E	139.0(2)		C01I	C020	C01S	121.0(8)
O00A	U002	N4	70.2(2)		C01I	C020	H020	119.5
O00E	U002	N4	68.9(2)		C01S	C020	H020	119.5
O1	U003	O2	68.6(2)		C014	C021	C01L	119.8(8)
O1	U003	O009	88.8(2)		C014	C021	C02E	118.8(8)
O1	U003	O00C	68.8(2)		C01L	C021	C02E	121.1(8)
O1	U003	O00D	94.2(2)		C01A	C022	C01I	121.3(8)
O1	U003	O00G	153.6(2)		C01A	C022	H022	119.3
O1	U003	N00M	135.6(2)		C01I	C022	H022	119.3
O2	U003	O009	90.9(2)		C01B	C024	H024	119.9
O2	U003	O00C	137.4(2)		C01B	C024	C02A	120.2(8)
O2	U003	O00D	89.8(2)		H024	C024	C02A	119.8
O2	U003	O00G	137.2(2)		C01N	C028	H028	118.5
O2	U003	N00M	68.6(2)		C01N	C028	C02G	123.1(8)
O009	U003	O00C	85.8(2)		H028	C028	C02G	118.4
O009	U003	O00D	177.0(2)		C01Y	C029	H029	119.8
O009	U003	O00G	85.9(2)		C01Y	C029	C02G	120.2(9)
O009	U003	N00M	80.5(2)		H029	C029	C02G	119.9
O00C	U003	O00D	95.7(2)		C01F	C02A	C024	120.3(9)
O00C	U003	O00G	85.0(2)		C01F	C02A	H02A	120
O00C	U003	N00M	151.0(2)		C024	C02A	H02A	120
O00D	U003	O00G	91.6(2)		H02B	C02B	C02D	120
O00D	U003	N00M	97.0(2)		H02B	C02B	C02M	120
O00G	U003	N00M	68.7(2)		C02D	C02B	C02M	119(1)
U001	O1	U002	114.2(2)		C01V	C02D	C02B	120.1(9)
U001	O1	U003	114.7(2)		C01V	C02D	H02D	120
U002	O1	U003	112.4(2)		C02B	C02D	H02D	120
U002	O2	U003	100.3(2)		C021	C02E	H02E	118.7
U002	O2	C2	131.5(4)		C021	C02E	C02N	122.5(9)
U003	O2	C2	124.6(4)		H02E	C02E	C02N	119
U001	O00A	U002	99.7(2)		C028	C02G	C029	118.5(9)
U001	O00A	C00R	131.2(5)		C028	C02G	H02G	120.8
U002	O00A	C00R	125.5(4)		C029	C02G	H02G	120.7
U001	O00C	U003	101.2(2)		C01W	C02H	H02H	120
U001	O00C	C1	129.9(4)		C01W	C02H	C02Q	120.2(9)
U003	O00C	C1	127.4(4)		H02H	C02H	C02Q	120
U002	O00E	C018	126.7(5)		C01T	C02I	H02I	120
U001	O00F	C01J	118.1(4)		C01T	C02I	C02N	121(1)
U003	O00G	C014	126.7(5)		H02I	C02I	C02N	119
U001	N3	C011	129.5(5)		C01V	C02J	H02J	121

U001	N3	C015	119.5(5)		C01V	C02J	C02L	119(1)
C011	N3	C015	105.2(6)		H02J	C02J	C02L	121
U002	N4	C00S	122.3(5)		C02J	C02L	H02L	119
U002	N4	C00Y	127.5(5)		C02J	C02L	C02M	121(1)
C00S	N4	C00Y	105.2(6)		H02L	C02L	C02M	119
H00K	N00K	N00L	127.9		C02B	C02M	C02L	120(1)
H00K	N00K	C015	127.9		C02B	C02M	H02M	120
N00L	N00K	C015	104.2(6)		C02L	C02M	H02M	120
N00K	N00L	C011	109.7(6)		C02E	C02N	C02I	119(1)
N00K	N00L	C01O	118.8(6)		C02E	C02N	H02N	121
C011	N00L	C01O	130.4(6)		C02I	C02N	H02N	121
U003	N00M	C013	128.8(5)		C01W	C02O	H02O	122
U003	N00M	C01L	121.7(5)		C01W	C02O	C02P	117(1)
C013	N00M	C01L	105.7(7)		H02O	C02O	C02P	122
O2	C2	C012	120.6(7)		C02O	C02P	H02P	118
O2	C2	C016	120.1(7)		C02O	C02P	C02R	123(1)
C012	C2	C016	119.3(7)		H02P	C02P	C02R	119
N00X	N00P	C00Y	110.3(7)		C02H	C02Q	H02Q	120
N00X	N00P	C01W	118.3(7)		C02H	C02Q	C02R	121(1)
C00Y	N00P	C01W	130.5(7)		H02Q	C02Q	C02R	120
H4	C4	C00R	119.6		C02P	C02R	C02Q	119(1)
H4	C4	C01D	119.6		C02P	C02R	H02R	120
C00R	C4	C01D	120.8(8)		C02Q	C02R	H02R	120
O00A	C00R	C4	121.3(7)		C01U	N00H	C023	113.5(6)
O00A	C00R	C3	120.4(7)		C01U	N00H	C026	108.5(7)
C4	C00R	C3	118.2(7)		C023	N00H	C026	112.1(7)
N4	C00S	N00X	111.7(7)		H01R	C01E	H01U	109.4
N4	C00S	C01B	125.7(7)		H01R	C01E	H01V	109.4
N00X	C00S	C01B	122.5(7)		H01R	C01E	C023	109.5
O00C	C1	C00Z	120.6(6)		H01U	C01E	H01V	109.4
O00C	C1	C01R	121.0(6)		H01U	C01E	C023	109.5
C00Z	C1	C01R	118.3(7)		H01V	C01E	C023	109.5
H00U	N00U	N2	127.6		N00H	C01U	H01W	109.3
H00U	N00U	C01L	127.4		N00H	C01U	H01	109.4
N2	N00U	C01L	105.0(7)		N00H	C01U	C027	111.1(8)
C00R	C3	C00Y	120.0(7)		H01W	C01U	H01	108
C00R	C3	C019	120.7(7)		H01W	C01U	C027	109.4
C00Y	C3	C019	119.2(7)		H01	C01U	C027	109.5
N00U	N2	C013	108.6(7)		N00H	C023	C01E	114.7(7)
N00U	N2	C01V	119.6(7)		N00H	C023	H02X	108.6
C013	N2	C01V	131.5(7)		N00H	C023	H02Y	108.7
N00P	N00X	C00S	103.9(6)		C01E	C023	H02X	108.5
N00P	N00X	H00X	128.1		C01E	C023	H02Y	108.6
C00S	N00X	H00X	128.1		H02X	C023	H02Y	107.6
N4	C00Y	N00P	109.0(7)		H02Z	C025	H	109.4

N4	C00Y	C3	123.9(7)		H02Z	C025	HA	109.6
N00P	C00Y	C3	126.9(7)		H02Z	C025	C026	109.5
C1	C00Z	H00Z	119.1		H	C025	HA	109.5
C1	C00Z	C01C	121.6(7)		H	C025	C026	109.4
H00Z	C00Z	C01C	119.3		HA	C025	C026	109.5
N3	C011	N00L	108.1(6)		N00H	C026	C025	115.5(8)
N3	C011	C01R	124.1(7)		N00H	C026	H02	108.4
N00L	C011	C01R	127.8(7)		N00H	C026	HB	108.3
C2	C012	H012	119.1		C025	C026	H02	108.4
C2	C012	C01Z	121.9(8)		C025	C026	HB	108.4
H012	C012	C01Z	119.1		H02	C026	HB	108
N00M	C013	N2	109.0(7)		C01U	C027	H1	109.4
N00M	C013	C016	125.4(8)		C01U	C027	HC	109.5
N2	C013	C016	125.4(8)		C01U	C027	HD	109.6
O00G	C014	C01T	118.8(8)		H1	C027	HC	109.4
O00G	C014	C021	122.7(8)		H1	C027	HD	109.4
C01T	C014	C021	118.4(8)		HC	C027	HD	109.5
N3	C015	N00K	112.7(7)		H00N	N00N	C010	106.3
N3	C015	C01N	124.5(7)		H00N	N00N	C017	106.2
N00K	C015	C01N	122.4(7)		H00N	N00N	C02F	106.2
C2	C016	C013	119.2(7)		C010	N00N	C017	109.8(7)
C2	C016	C01M	118.9(8)		C010	N00N	C02F	113.3(7)
C013	C016	C01M	121.8(8)		C017	N00N	C02F	114.4(7)
O00E	C018	C01B	121.7(7)		N00N	C010	H01B	108.5
O00E	C018	C01Q	120.5(7)		N00N	C010	H01E	108.5
C01B	C018	C01Q	117.8(7)		N00N	C010	C01G	115.0(7)
C3	C019	H019	120.4		H01B	C010	H01E	107.5
C3	C019	C01P	119.3(8)		H01B	C010	C01G	108.6
H019	C019	C01P	120.4		H01E	C010	C01G	108.5
H01A	C01A	C01O	120.9		N00N	C017	H01G	108.5
H01A	C01A	C022	120.8		N00N	C017	H01J	108.7
C01O	C01A	C022	118.3(7)		N00N	C017	C02K	114.7(8)
C00S	C01B	C018	120.8(7)		H01G	C017	H01J	107.6
C00S	C01B	C024	119.8(7)		H01G	C017	C02K	108.5
C018	C01B	C024	119.4(7)		H01J	C017	C02K	108.6
C00Z	C01C	H01C	119.7		C010	C01G	H01L	109.4
C00Z	C01C	C01K	120.6(8)		C010	C01G	H01N	109.4
H01C	C01C	C01K	119.8		C010	C01G	H01O	109.5
C4	C01D	H01D	119.4		H01L	C01G	H01N	109.4
C4	C01D	C01P	121.2(9)		H01L	C01G	H01O	109.5
H01D	C01D	C01P	119		H01N	C01G	H01O	109.6
H01F	C01F	C01Q	119.4		H02C	C02C	H02F	109
H01F	C01F	C02A	119.3		H02C	C02C	H02K	109
C01Q	C01F	C02A	121.4(9)		H02C	C02C	C02F	110
H01H	C01H	C01K	118.8		H02F	C02C	H02K	109

H01H	C01H	C01R	118.8		H02F	C02C	C02F	110
C01K	C01H	C01R	122.4(8)		H02K	C02C	C02F	110
H01I	C01I	C020	121		N00N	C02F	C02C	112.7(8)
H01I	C01I	C022	120.9		N00N	C02F	H02S	109
C020	C01I	C022	118.2(8)		N00N	C02F	H02T	109.1
O00F	C01J	C01N	123.0(7)		C02C	C02F	H02S	109
O00F	C01J	C01Y	118.9(7)		C02C	C02F	H02T	109
C01N	C01J	C01Y	118.1(7)		H02S	C02F	H02T	107.9
C01C	C01K	C01H	118.4(8)		C017	C02K	H02U	109.4
C01C	C01K	H01K	120.8		C017	C02K	H02V	109.4
C01H	C01K	H01K	120.8		C017	C02K	H02W	109.5
N00M	C01L	N00U	111.8(7)		H02U	C02K	H02V	109
N00M	C01L	C021	126.4(7)		H02U	C02K	H02W	110
N00U	C01L	C021	121.8(8)		H02V	C02K	H02W	110

References

1 A. C. Sedgwick, K. C. Yan, D. N. Mangel, Y. Shang, A. Steinbrueck, H. H. Han, J. T. Brewster II, X. L. Hu, D. W. Snelson, V. M. Lynch, H. Tian, X. P. He, J. L. Sessler, *J. Am. Chem. Soc.*, 2021, **143**, 1278.
2 J. L. Kiplinger, D. E. Morris, B. L. Scott, C. J. Burns, *Organometallics*, 2002, **21**, 5978.
3 H. Kitagawa, T. Shibata, J. Matsuo, T. Mukaiyama, *Bull. Chem. Soc. Jpn.*, 2002, **75**, 339.
4 Bruker Instrument Service, Version 2010.1.0.0, Bruker AXS Inc., Madison, WI, USA, 2010.
5 SAINT Plus, Data Reduction Software, Version 7.68A, Bruker AXS Inc., Madison, WI, USA, 2009.
6 G. M. Sheldrick, *Acta. Cryst.*, 2008, **A64**, 112.
7 G. M. Sheldrick, SADABS, University of Göttingen, Germany, 2005.
8 G. M. Sheldrick, *Acta. Cryst. Section A*, 2015, **A71**, 3.
9 APEX2, Data Refinement Software, Version 2010.12, Bruker AXS Inc., Madison, WI, USA, 2010.
10 Olex2 1.2 (compiled 2014.06.27 svn.r2953 for OlexSys, GUI svn.r4855).
11 O. V. Dolomanov, L. J. Bourhis, R. J. Gildea, J. A. K. Howard, H. Puschmann, *J. Appl. Cryst.*, 2009, **42**, 339.
12 T. Muntener, D. Joss, D. Haussinger, S. Hiller, *Chem. Rev.*, 2022, **122**, 9422.
13 H. W. Orton, T. Huber, G. Otting, ***Magn. Reson.*** 2020, **1**, 1.
14 D. J. Wales, J. P. K. Doye, *J. Phys. Chem. A*, 1997, **101**, 5111.
